# Prediabetes Induced by Fructose-Enriched Diet Influences Cardiac Lipidome and Proteome and Leads to Deterioration of Cardiac Function prior to the Development of Excessive Oxidative Stress and Cell Damage

**DOI:** 10.1155/2019/3218275

**Published:** 2019-12-09

**Authors:** Gergő Szűcs, Andrea Sója, Mária Péter, Márta Sárközy, Bella Bruszel, Andrea Siska, Imre Földesi, Zoltán Szabó, Tamás Janáky, László Vígh, Gábor Balogh, Tamás Csont

**Affiliations:** ^1^Metabolic Diseases and Cell Signaling Group, Department of Biochemistry, Faculty of Medicine, University of Szeged, Szeged H-6720, Hungary; ^2^Interdisciplinary Centre of Excellence, University of Szeged, Szeged H-6720, Hungary; ^3^Institute of Biochemistry, Biological Research Center of the Hungarian Academy of Sciences, Szeged H-6726, Hungary; ^4^Institute of Medical Chemistry, Faculty of Medicine, University of Szeged, Szeged H-6720, Hungary; ^5^Department of Laboratory Medicine, Faculty of Medicine, University of Szeged, Szeged H-6720, Hungary

## Abstract

Prediabetes is a condition affecting more than 35% of the population. In some forms, excessive carbohydrate intake (primarily refined sugar) plays a prominent role. Prediabetes is a symptomless, mostly unrecognized disease which increases cardiovascular risk. In our work, we examined the effect of a fructose-enriched diet on cardiac function and lipidome as well as proteome of cardiac muscle. Male Wistar rats were divided into two groups. The control group received a normal diet while the fructose-fed group received 60% fructose-supplemented chow for 24 weeks. Fasting blood glucose measurement and oral glucose tolerance test (OGTT) showed slightly but significantly elevated values due to fructose feeding indicating development of a prediabetic condition. Both echocardiography and isolated working heart perfusion performed at the end of the feeding protocol demonstrated diastolic cardiac dysfunction in the fructose-fed group. Mass spectrometry-based, high-performance lipidomic and proteomic analyses were executed from cardiac tissue. The lipidomic analysis revealed complex rearrangement of the whole lipidome with special emphasis on defects in cardiolipin remodeling. The proteomic analysis showed significant changes in 75 cardiac proteins due to fructose feeding including mitochondria-, apoptosis-, and oxidative stress-related proteins. Nevertheless, just very weak or no signs of apoptosis induction and oxidative stress were detected in the hearts of fructose-fed rats. Our results suggest that fructose feeding induces marked alterations in the cardiac lipidome, especially in cardiolipin remodeling, which leads to mitochondrial dysfunction and impaired cardiac function. However, at the same time, several adaptive responses are induced at the proteome level in order to maintain a homeostatic balance. These findings demonstrate that even very early stages of prediabetes can impair cardiac function and can result in significant changes in the lipidome and proteome of the heart prior to the development of excessive oxidative stress and cell damage.

## 1. Introduction

Diabetes mellitus is a heterogeneous chronic metabolic disorder characterized by hyperglycemia [1]. The number of people suffering from diabetes increased from 108 million in 1980 to 422 million by 2014, and global prevalence almost doubled since 1980, from 4.7% to 8.5% [2]. According to the International Diabetes Federation, the number of people with diabetes may rise to 629 million by 2045 [3]. Prediabetes—in which glucose levels do not meet the criteria for diabetes but are too high to be considered normal—usually precedes diabetes mellitus and may remain symptomless for several years [4]. Prediabetes affects more than 35% of the population, and it is known that even nondiabetic levels of hyperglycemia and impaired glucose tolerance may be associated with an elevated risk of cardiovascular disease [5]. It has been recently shown that a mild diastolic dysfunction occurs even in prediabetic rats [6].

Type 2 diabetes is associated with myocardial lipotoxicity [7], which can cause impaired mitochondrial function [8]. Impaired mitochondrial function enhances oxidative stress, activates apoptosis, and thus contributes to cardiac dysfunction [7, 9, 10]. Although the role of lipotoxicity, oxidative stress, and apoptosis in diabetes has been well studied, the role of these mechanisms in prediabetes has not yet been well described. Saccharose and high-fructose corn syrup (isoglucose) are often used as sweeteners in the food and drink industry, and the consumption of these fructose-rich foods or beverages has an adverse effect on both animals [11] and humans [12]. A high-fructose diet is often used as a model of prediabetes or impaired glucose tolerance. After absorption, fructose is rapidly and uncontrollably absorbed in the liver, where its metabolism increases de novo lipogenesis (DNL). Induction of DNL has the capacity to alter the circulating nonesterified fatty acid (FA) profile, which, in turn, might affect cardiac lipid composition [13].

Proper cardiac lipid composition is strongly correlated with cardiac function and largely relies on proper cardiolipin (CL) content and species profile [14]. CL is the hallmark phospholipid (PL) of mitochondria that plays a role in many mitochondrial processes, including respiration and energy conversion. The heart is full of mitochondria, and CL accounts for about 10-15 mol% of all membrane lipids. Changes in the CL pool due to either oxidation or pathological remodeling cause mitochondrial dysfunctions and trigger retrograde signaling pathways that are associated with a large number of cardiac diseases including diabetes [15]. It is widely accepted that the symmetric tetra-linoleoyl (18:2) CL species, which constitutes up to 80% of mammalian cardiac CL, is required for mitochondria to work optimally in metabolically active tissues [16]. After its initial biosynthesis, premature CL undergoes intensive remodeling processes to produce maturated CL (Supplementary Lipid [Supplementary-material supplementary-material-1]) [14, 15, 17–19]. In the first step of maturation, the removal of a single acyl chain is executed by a calcium-independent phospholipase A_2_ to produce monolysocardiolipin (MLCL). Reacylation can be carried out by CoA-dependent acyltransferases or a CoA-independent reversible PL-lysoPL (LPL) transacylase called tafazzin. Tafazzin itself lacks acyl chain preference; still, it is believed to be the major enzyme involved in CL remodeling to produce homo-acylated CL [16]. Mutations in tafazzin cause abnormal molecular species of CL and the clinical phenotype of Barth syndrome, a rare and often fatal x-linked genetic disorder characterized by dilated cardiomyopathy, skeletal myopathy, and neutropenia [20].

Although CL is known to be relatively resistant to dietary manipulations, by “appropriate” interventions, the linoleoyl chain can be replaced [21]. Fructose feeding might represent such an intervention due to its highly lipogenic nature. Therefore, our study is aimed at examining the effects of fructose-enriched diet on the interplay of cardiac function, cardiac lipidome and proteome, and oxidative stress and apoptosis in a rat prediabetes model. To achieve this goal, several methods and techniques were applied including conventional blood tests, detailed serum analysis, enzymatic assays, protein expression analyses, transthoracic echocardiography, and high-performance mass spectrometry- (MS-) based proteomics and lipidomics.

## 2. Materials and Methods

This investigation conformed to the National Institutes of Health *Guide for the Care and Use of Laboratory Animals* (NIH Publication No. 85-23, Revised 1996) and was approved by the Animal Research Ethics Committee of University of Szeged.

### 2.1. Experimental Design

**Figure 1:**
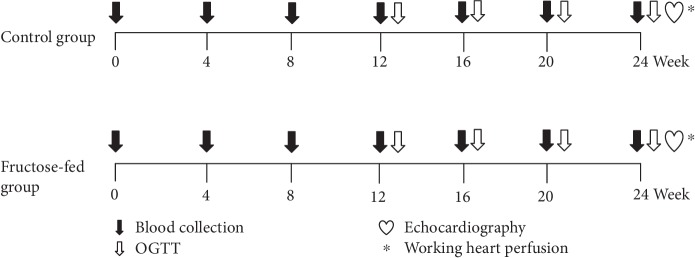
Experimental protocol. Male Wistar rats were divided into control (*n* = 8) and fructose-fed (*n* = 8) groups receiving either a standard chow or a chow supplemented with 60% fructose, respectively, for 24 weeks. Fasting blood glucose measurement or oral glucose tolerance test (OGTT) was performed every four weeks to monitor the development of prediabetic condition. At week 24, transthoracic echocardiography was performed to monitor cardiac function and morphology. Then, the hearts of the animals were isolated and mounted on a working heart perfusion system to measure hemodynamic and pressure parameters. After the perfusions, hearts were frozen for measurement of biochemical parameters.

Male Wistar rats (310–450 g, *n* = 16 in the entire study) were kept under controlled temperature with 12/12 h light/dark cycles. Animals were divided into two groups and were fed with the following diets for 24 weeks: the control group (*n* = 8) was fed with a standard laboratory chow, while the fructose-fed group (*n* = 8) received a chow containing 60% fructose. Fasting blood glucose was measured every 4 weeks, while at weeks 12, 16, 20, and 24 oral glucose tolerance tests (OGTT) were performed. At week 20 and week 24, blood samples were taken to measure serum parameters. At the end of the feeding protocol, cardiac function was assessed by both in vivo echocardiography and ex vivo working heart perfusions (Figure 1). Following the perfusions, myocardial tissue was harvested for biochemical analysis.

### 2.2. Transthoracic Echocardiography

Cardiac morphology and function were assessed by transthoracic echocardiography at week 24 as described previously [22–24]. Briefly, rats were anesthetized with sodium pentobarbital (Euthasol, 40 mg/kg body weight i.p.). Then, the chest was shaved, and the rat was placed in a supine position onto a heating pad. Two-dimensional, M-mode, and Doppler echocardiographic examinations were performed by the criteria of the American Society of Echocardiography with a Vivid IQ ultrasound system (General Electric Medical Systems) using a phased array 5.0–11 MHz transducer (GE 12S-RS probe). Data of three consecutive heart cycles were analyzed (EchoPac Dimension software; General Electric Medical Systems) by an experienced investigator in a blinded manner. Systolic and diastolic wall thickness parameters were obtained from parasternal short-axis view at the level of the papillary muscles (anterior and inferior walls) and long-axis view at the level of the mitral valve (septal and posterior walls). The left ventricular diameters were measured by means of M-mode echocardiography from long-axis views between the endocardial borders. Fractional shortening (FS) was used as a measure of cardiac contractility (FS = (LVEDD − LVESD)/LVEDD × 100). Functional parameters including left ventricular end-diastolic volume (LVEDV) and left ventricular end-systolic volume (LVESV) were calculated on four-chamber view images delineating the endocardial borders in diastole and systole. The stroke volume was calculated as the difference of LVEDV and LVESV. The ejection fraction (EF) was calculated according to the formula (LVEDV − LVESV)/LVEDV∗100. Diastolic function was assessed using pulse-wave Doppler across the mitral valve from the apical four-chamber view. Early (E) and atrial (A) flow velocities provide an indication of diastolic function. Heart rate was also calculated using pulse-wave Doppler images during the measurement of transvalvular flow velocity profiles according to the length of 3 consecutive heart cycles measured between the start points of the E waves. The mean values of three measurements were calculated and used for statistical evaluation.

### 2.3. Working Heart Perfusion

Immediately after the echocardiography, cardiac performance was assessed in isolated working rat hearts, as described earlier [25–27]. Anesthetized rats were given 500 U·kg^−1^ heparin intravenously. Hearts were then isolated, and the aorta was cannulated and initially perfused in Langendorff mode (at a constant pressure of 73 mmHg, 37°C) with Krebs-Henseleit buffer containing NaCl 118 mM, NaHCO_3_ 25 mM, KCl 4.3 mM, CaCl_2_ 2.4 mM, KH_2_PO_4_ 1.2 mM, MgSO_4_ 1.2 mM, and glucose 11 mM, gassed with 95% O_2_ and 5% CO_2_ [22, 28]. Then, the perfusion system was switched to working mode according to Neely with recirculating buffer [28, 29]. Hydrostatic preload and afterload were kept constant at 13 mmHg and 73 mmHg, respectively, throughout the experiments. Hearts were subjected to 10 min equilibration period before measurement (*n* = 7–8). Cardiac functional parameters including heart rate, coronary flow, aortic flow, cardiac output, left ventricular developed pressure (LVDP) and its first derivatives (dp/dt max and dp/dt min), and left ventricular end-diastolic pressure (LVEDP) were measured. At the end of the perfusion, the hearts were weighed, and the left and right ventricles were separated. The right and left ventricles were snap frozen in liquid nitrogen and stored at −80°C until they were used for biochemical assays.

### 2.4. Measurement of Malondialdehyde Levels

In order to measure the level of systemic and cardiac lipid peroxidation, serum malondialdehyde and left ventricular tissue malondialdehyde were assayed spectrophotometrically at 535 nm as described previously [27, 30]. Results are expressed as nmol/mL serum and nmol/mg protein.

### 2.5. mRNA Expression Profiling by qRT-PCR

Quantitative RT-PCR was performed with gene-specific primers to monitor mRNA expression as described previously [24]. To assess de novo lipid synthesis, expression of sterol regulatory element-binding transcription factor 1 (Srebf1), stearoyl-CoA desaturase 1 (Scd1), stearoyl-CoA desaturase 2 (SCD2), fatty acid synthase (Fasn), acetyl-CoA carboxylase 1 (Acaca), carbohydrate-responsive element-binding protein (Mlxipl), elongation of very-long-chain fatty acids protein 6 (ELOVL6), fatty acid desaturase 1 (Fads1), and fatty acid desaturase 2 (Fads2) were measured from liver samples. To assess cardiac hypertrophy, expression of myosin heavy chain *α* isoform (MYH6) and myosin heavy chain *β* isoform (MYH7) was measured. RNA was isolated using Qiagen RNeasy Fibrous Tissue Mini Kit (Qiagen, #74704) from the liver and heart tissues. Briefly, 4 *μ*g and 2.2 *μ*g of total RNA from liver and heart samples, respectively, were reverse transcribed using iScript™ Advanced cDNA Synthesis kit (Bio-Rad, 1725038), and specific primers and SsoAdvanced™ Universal SYBR® Green Supermix (Bio-Rad) were used according to the manufacturer's instructions. Hypoxanthine phosphoribosyltransferase 1 (Hprt1) was used as control for normalization.

### 2.6. Lipidomics

Approximately 20 mg of the powdered left ventricle was directly extracted by adding 1 mL of methanol containing 0.001% butylated hydroxytoluene as an antioxidant and 60 *μ*g di20:0 phosphatidylcholine as extraction standard. After a 5 min sonication in a water bath sonicator, the mixture was shaken for 5 min and centrifuged at 10000 × g for 5 min. The supernatant was transferred into a new Eppendorf tube and stored at −20°C until mass spectrometry (MS) analysis.

The solvents used for extraction and MS analyses were of Optima LCMS grade from Thermo Fisher Scientific (Bremen, Germany) and liquid chromatographic grade from Merck (Darmstadt, Germany). Lipid standards were purchased from Avanti Polar Lipids (Alabaster, AL). All other chemicals were from Sigma-Aldrich (Steinheim, Germany) and were of the best available grade.

Mass spectrometric analyses were performed on an LTQ-Orbitrap Elite instrument (Thermo Fisher Scientific, Bremen, Germany) equipped with a robotic nanoflow ion source (TriVersa NanoMate; Advion BioSciences; Ithaca, NY, USA) as described in [31]. Further details of MS measurements and lipid species annotation are given in the Supplementary Methods.

### 2.7. Proteomics

Approximately 30 mg of powdered left ventricle tissue samples was homogenized in lysate buffer (containing 2% SDS and 0.1 M DTT in 0.1 Tris solution). The homogenized samples were incubated at 98°C for 5 min. Proteins were precipitated by the addition of methanol/chloroform mixture (4 : 1) and were resuspended in 8 M urea. The total protein contents were determined using BCA (Thermo) protocol. 20 *μ*g protein was digested by trypsin (Thermo) using RapiGest (Waters) detergent to enhance the digestion. In-gel fractionation was performed for pooled sample. In-gel fractionated samples were also digested by trypsin.

1D gel samples and pooled samples were measured in DDA (data-dependent acquisition) using a 90 min gradient on a Waters nanoAcquity-Thermo Q Exactive Plus LC-MS system to build a spectrum library of detectable peptides. The individual samples were measured in DIA (Data Independent Acquisition) mode for protein quantification with the same LC gradient using the spectrum library. The acquired data were analyzed with Encyclopedia [32] and statistically evaluated using Perseus [33] software. Enrichment of significantly changing proteins according to subcellular localization was carried out by Gene Ontology analysis (https://www.ebi.ac.uk/QuickGO/). Pathway assignment analysis of significantly altered proteins was performed with Reactome (https://www.reactome.org) after assignment to human genes, for higher annotation coverage. Further details of proteomic analysis are given in the Supplementary Methods.

### 2.8. Measurement of Serum and Pancreatic Insulin Levels

Serum and pancreatic insulin levels were measured by an enzyme immunoassay (Mercodia, Ultrasensitive Rat Insulin ELISA) in order to verify the development of hyperinsulinemia and decreased pancreatic insulin content as a consequence of beta cell damage in impaired glucose tolerance. Insulin ELISA was carried out according to the instructions of the manufacturer from either sera or homogenized pancreatic tissue samples of fructose-fed and control rats. Sera were centrifuged (2000 g for 10 min at 4°C) and kept at -20°C until further investigation. Pancreata were removed, trimmed free of adipose tissue, and weighed. Pancreata were homogenized in 6 mL cold acidified ethanol (0.7 M HCl : ethanol (1 : 3 *v*/*v*)) with an Ultra Turrax homogenizer and were kept at 4°C for 24 h. Then, pancreas homogenates were centrifuged (900 g for 15 min at 4°C), and the supernatants were stored at 4°C. The pellet was extracted again with 3 mL acidified ethanol for 24 h at 4°C. The supernatant obtained after centrifugation was pooled with the previous one and kept at -20°C until assayed [34, 35].

### 2.9. HOMA-IR Index

To estimate insulin resistance in fructose-fed or control rats, the widely used HOMA-IR index was calculated [34, 36, 37] by multiplying fasting serum insulin (*μ*U/mL) with fasting serum glucose (mmol/L) then dividing by the constant 22.5, i.e., HOMA‐IR = (fasting serum insulin concentration × fasting serum glucose concentration)/22.5.

### 2.10. Measurement of Serum Lipid Levels

Serum cholesterol, triglyceride, LDL, and HDL levels were measured at week 24 using a test kit supplied by Diagnosticum Zrt. (Budapest, Hungary) as described previously [27].

### 2.11. 3-NT ELISA

A double-antibody sandwich ELISA kit specific for 3-nitrotyrosine measurement was purchased from Genasiabio (Shanghai, China). Left ventricles were homogenized (Heilscher UP100H Ultrasonic Processor) in Phosphate Buffer Saline (PBS) (pH 7.2–7.4) and then centrifuged at 3000 rpm for 20 min at 4°C. Nitrotyrosine was measured according to the manufacturer's instructions and protocols, and optical densities (OD) were determined at 450 nm. Results were expressed as nmol/mg protein.

### 2.12. Measurement of Serum Laboratory Parameters

Urea and creatinine levels in serum were quantified by kinetic UV method using urease and glutamate dehydrogenase enzymes and Jaffe method, respectively. The reagents and the platform analyzers were from Roche Diagnostics. Serum sodium, potassium, and chloride levels were determined by indirect potentiometry using ion-selective electrodes at week 24. All reagents and instruments were from Roche Diagnostics. Alanine aminotransferase (ALAT), aspartate aminotransferase (ASAT), creatine kinase (CK), and lactate dehydrogenase (LDH) enzyme activities were measured with Roche UV assays standardized according to the recommendations of IFCC (International Federation of Clinical Chemistry). Creatine kinase MB enzyme activities were determined using an immunological UV assay of Roche.

### 2.13. Western Blot

To investigate changes of apoptotic proteins, the standard western blot technique was used in case of Bax, Bcl-2, Bcl-xL, caspase-7, and caspase-3 with actin or tubulin loading background. Left ventricular samples (*n* = 8) were homogenized with an ultrasonicator (UP100H Hielscher, Teltow, Germany) in Radio-Immunoprecipitation Assay (RIPA) buffer (50 mM Tris-HCl (pH 8.0)), 150 mM NaCl, 0.5% sodium deoxycholate, 5 mM ethylenediamine tetra-acetic acid (EDTA), 0.1% sodium dodecyl sulfate, 1% NP-40 (Cell Signaling, Carlsbad, CA, USA) supplemented with phenylmethanesulfonyl fluoride (PMSF). The crude homogenates were centrifuged at 15000 × g for 30 min at 4°C. After quantification of protein concentrations of the supernatants using the BCA Protein Assay Kit (Pierce, Rockford, IL, USA), 25 *μ*g of reduced and denaturized protein was loaded. Then, sodium dodecyl-sulfate polyacrylamide gel electrophoresis (SDS-PAGE) was performed (10% gel, 50 V, 4 h) followed by the transfer of proteins onto a nitrocellulose membrane (20% methanol, 35 V, 1.5 h). The efficacy of transfer was checked using Ponceau staining. The membranes were cut horizontally into parts corresponding to the molecular weights of Bax, Bcl-2, Bcl-xL, caspase-7, caspase-3, actin, and tubulin. Membranes were blocked for 1 h in 5% (*w*/*v*) bovine serum albumin (BSA) and were incubated with primary antibodies in the concentrations of 1 : 1000 against Bax (#2772), Bcl-2 (#3498), Bcl-xL (#2764), caspase-7 (#12827), caspase-3 (#14220), *α*-tubulin (#2144), and *β*-actin (#4970) overnight at 4°C in 5% BSA. Then, the membranes were incubated with IRDye® 800CW Goat Anti-Rabbit secondary antibody (Li-Cor) for 1 h at room temperature in 5% BSA. Fluorescent signals were detected by Odyssey CLx, and digital images were analyzed and evaluated by Quantity One Software.

### 2.14. Statistical Analysis

Proteomic data are presented as mean intensities ± CV, fold change, and *p* value. For proteomic data, the statistical significance was tested using unpaired Welch test. *p* < 0.05 and a fold change > 1.5 were accepted as a statistically significant difference. Lipidomic data are presented as mean ± SEM; statistical significance was determined according to Storey and Tibshirani [38] and was accepted for *p* < 0.05 corresponding to a false discovery rate < 0.05. PCA analyses were performed using MetaboAnalyst [39]. All other parameters are presented as mean ± SEM, and significance between groups was determined with two sample *t*-test or Mann-Whitney Rank Sum Test.

## 3. Results and Discussion

### 3.1. Prediabetes and Characterization of the Animal Model

**Figure 2:**
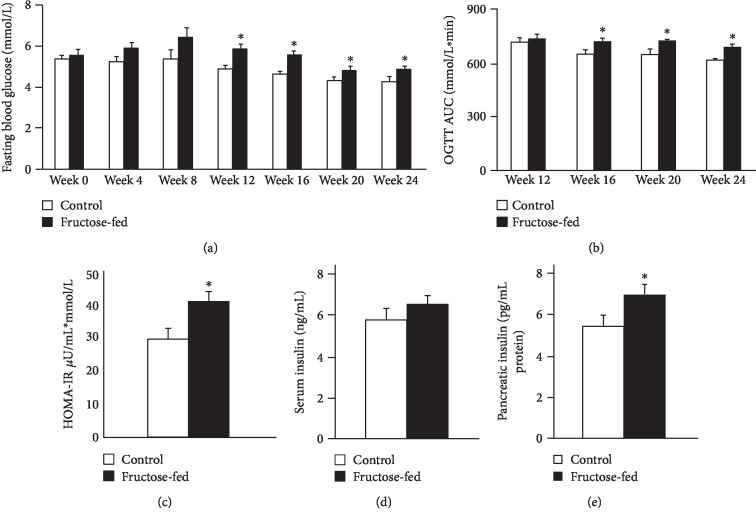
Prediabetic condition: (a) fasting blood glucose levels, (b) area under the curve (AUC) values for OGTT, (c) HOMA-IR index at week 20, (d) serum insulin level at week 20, and (e) pancreatic insulin level at week 24. Values are mean ± SEM (*n* = 7‐8), ^∗^*p* < 0.05.

In the present study, male Wistar rats were fed with 60% fructose-containing chow for 24 weeks to create a model of prediabetes. We have chosen this model in order to examine the effect of a moderate metabolic condition on the heart, rather than looking at the effects of severely disturbed glucose and lipid homeostasis seen for instance in genetically modified diabetes models (e.g., db/db or ob/ob mice) [40, 41]. In order to verify the development of the prediabetic state, fasting blood glucose was measured at every 4th week, and OGTT was performed at weeks 12, 16, 20, and 24. Fasting glucose levels were slightly but significantly higher in fructose-fed rats at weeks 12, 16, 20, and 24 (Figure 2(a)). OGTT area under the curve values were also significantly increased in fructose-fed rats at weeks 16, 20, and 24 (Figure 2(b)). These results demonstrate the development of prediabetes with impaired glucose tolerance in the present model. HOMA-IR, a widely used indicator of insulin resistance, was significantly higher in the fructose-fed rats at week 20, although no significant difference was detected in serum insulin levels (Figure 2(c)). Pancreatic insulin level was significantly higher in the fructose-fed group compared to controls (Figure 2(e)). These data demonstrate the appearance of a mild insulin resistance in our present model.

**Figure 3:**
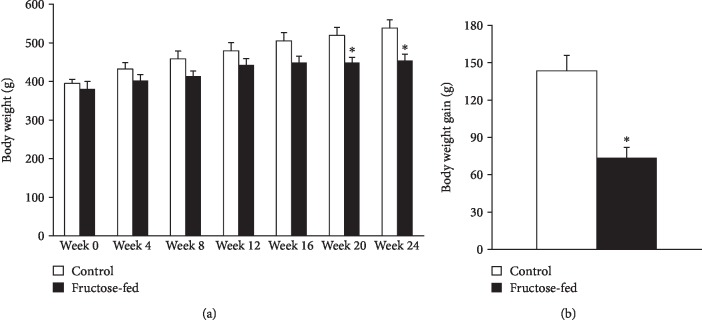
Body weight and weight gain of control and fructose-fed rats. Values are mean ± SEM (*n* = 8), ^∗^*p* < 0.05.

Although body weight increased in both groups during the course of the study, by the end of 24-week feeding, the weight of the fructose-fed rats was significantly smaller compared to that of the control rats (Figure 3(a)). Weight gain during the study was decreased in fructose-fed rats (Figure 3(b)). Although liver weight was not significantly different in fructose-fed rats, the liver weight to body weight ratio was increased (Table 1). Moreover, during the isolation of organs, we have observed macroscopical signs of fatty degeneration on the liver of fructose-fed animals. These findings may indicate fatty degeneration in the liver due to DNL initiated by fructose feeding. In fact, it has been demonstrated that fructose may activate DNL due to its rapid conversion to pyruvate bypassing the regulatory step of glycolysis catalyzed by the phosphofructokinase-1 enzyme [42]. Compared to the effects of fat-supplemented diet which leads to fat deposits both in the liver and adipose tissue (liver as well as body weight gain), dietary fructose preferably increases lipid accumulation only in the liver [42]. Fructose feeding may affect the metabolism of skeletal muscle through metabolic stress. For instance, Gatineau et al. showed that older rats fed with fructose-containing diet lost significantly more lean body mass and maintained more adipose tissue than control rats [43]. In sucrose-fed rats, significantly lower diet-induced muscle protein synthesis was observed compared to starch-fed rats [43]. Additionally, excessive fructose consumption was shown in the liver to increase production of substances such as methylglyoxal, which leads to oxidative stress in the muscle [44]. Activated DNL leads to endoplasmic reticulum stress [45] and production of hepatokines, such as fetuin-A [46], known to adversely affect muscle energy metabolism and insulin sensitivity [47]. These findings might explain decreased body weight gain in fructose-fed rats in our present study. Despite the macroscopic signs of fatty degeneration in the liver, neither serum lipid parameters (triglycerides, total cholesterol, and LDL and HDL cholesterol) nor liver enzymes (ALAT, ASAT) were increased in fructose-fed rats (Table 2) indicating an early stage of hepatic consequences.

**Figure 4:**
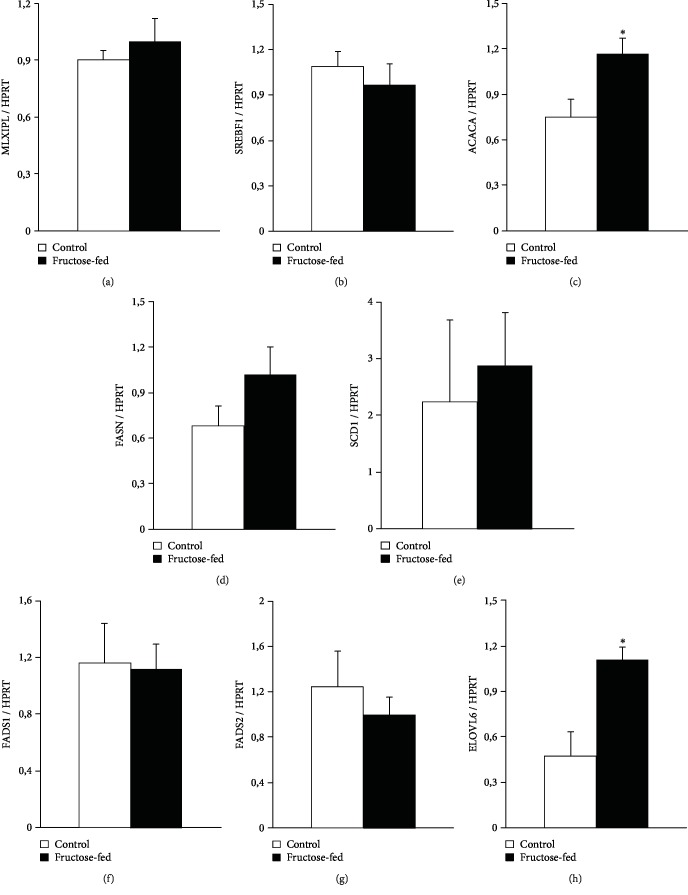
qRT-PCR results at week 24. Liver (a) Mlxipl expression, (b) Srebf1 expression, (c) Acaca expression, (d) Fasn expression, (e) SCD1 expression, (f) Fads1 expression, (g) Fads2 expression, and (h) ELOVL6 expression. Values are mean ± SEM (*n* = 6‐7), ^∗^*p* < 0.05.

To further characterize metabolic changes in the liver of fructose-fed rats, we performed qRT-PCR. We examined Srebf1 and Mlxipl transcription factors which regulate fatty acid metabolism related genes. No difference was found between control and fructose-fed group. We also examined Acaca and Fasn. Acaca catalyzes the carboxylation of acetyl-CoA to malonyl-CoA, the rate-limiting step of fatty acid synthesis. Fasn catalyzes the remaining steps of palmitic acid synthesis. Fasn expression showed a tendency of increase, while Acaca expression significantly increased in fructose-fed rats (Figure 4(c)). These findings are consistent with previous results and clearly indicate increased de novo lipid synthesis in fructose-fed rats [48, 49]. ELOVL6 enzyme, which catalyzes the first and rate-limiting reaction of long-chain fatty acid elongation cycle, was also significantly increased in fructose-fed rats. ELOVL6 enzyme is also known to play an important role in nonalcoholic fatty liver disease and steatohepatitis [50, 51] (Figure 4(h)).

### 3.2. Heart Function and Morphology

To characterize prediabetes-induced cardiac changes in fructose-fed rats, transthoracic echocardiography was performed at week 24 to investigate cardiac function. Although the weight of the animals was significantly lower in the fructose-fed group, the heart weight and heart weight to body weight ratio were not changed significantly (Table 1).

Echocardiographic parameters of morphology and function are shown in Table 3. To exclude the potential effect of variations in cardiac mass, the morphological data were also given after normalization to heart weight (Table 3). Wall thicknesses and ventricular diameters were not changed significantly due to fructose feeding (except for anterior wall thickness) (Table 3). Although there was no difference in heart rate, ejection fraction, and fractional shortening, the E/A ratio was significantly smaller in fructose-fed rats indicating the impairment of diastolic filling (Table 3). These findings may suggest a very early manifestation of a mild hypertrophy and diastolic dysfunction with preserved systolic function in prediabetic rats.

**Figure 5:**
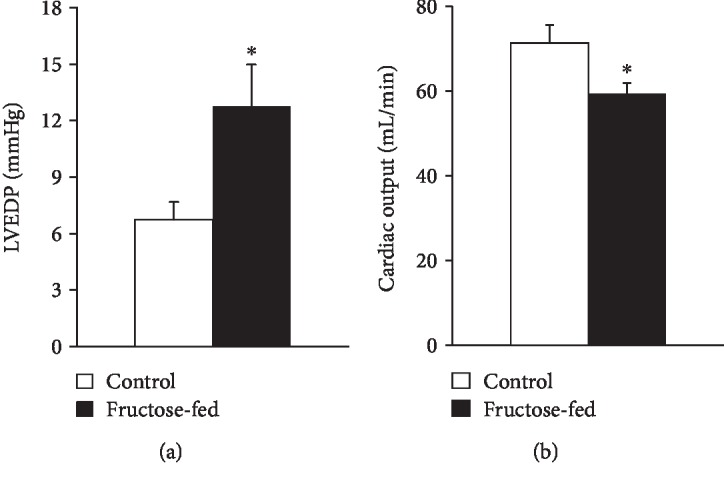
Cardiac function in isolated perfused hearts: (a) left ventricular end-diastolic pressure (LVEDP) and (b) cardiac output. Values are mean ± SEM (*n* = 7‐8), ^∗^*p* < 0.05.

Following echocardiography, the hearts were isolated to assess cardiac performance on a working heart perfusion system. Left ventricular end-diastolic pressure significantly increased, while cardiac output significantly decreased in fructose-fed rats (Figure 5). However, HR, max and min dp/dt, LVDP, and aortic systolic and diastolic pressures were not changed between groups during working heart perfusion (Table 4). These results demonstrate the appearance of a mild diastolic dysfunction in prediabetic rats. It is well known that left ventricular hypertrophy is more common in diabetic patients and that 40-75% of patients with type 1 or type 2 diabetes have diastolic dysfunction [52, 53]. However, here we show that the deterioration of diastolic function occurs much earlier than the development of overt diabetes. These results are consistent with recent reports showing that the development of diastolic dysfunction can precede complete diabetes [6].

**Figure 6:**
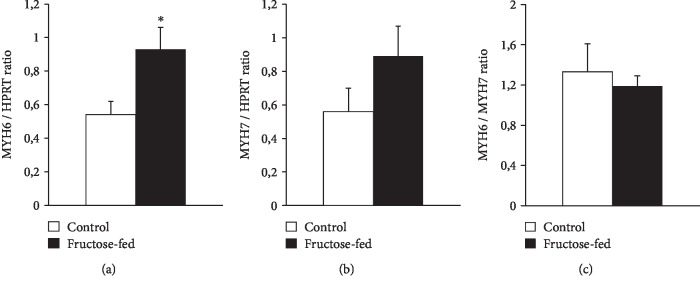
qRT-PCR results at week 24. Heart (a) MYH6 expression, (b) MYH7 expression, and (c) MYH6/MYH7 ratio. Values are means ± SEM (n = 8), ^∗^*p* < 0.05.

In order to assess cardiac hypertrophy at the molecular level, mRNA expression of myosin heavy chain *α* isoform (MYH6) and myosin heavy chain *β* isoform (MYH7) was measured. We have found that cardiac MYH6 mRNA level, consistent with myosin 6 protein level measured by proteomics (Table 5), was increased in fructose-fed rats (Figure 6). However, MYH6/MYH7 ratio did not differ significantly. According to the literature [54–56], these data do not support cardiac hypertrophy in our fructose-fed rats.

It is known that clinical laboratory markers of myocardial injury are increased in diabetic cardiomyopathy [57] and serum ion parameters, especially potassium, can affect heart function. Therefore, we measured serum ions (potassium, sodium, and chloride) and enzyme markers of myocardial injury (creatine kinase (CK), creatine kinase-MB (CK-MB), and lactate dehydrogenase (LDH)). Neither serum ion parameters nor markers of myocardial injury were changed significantly in fructose-fed rats compared to controls (Table 2).

### 3.3. Lipidomics

**Figure 7:**
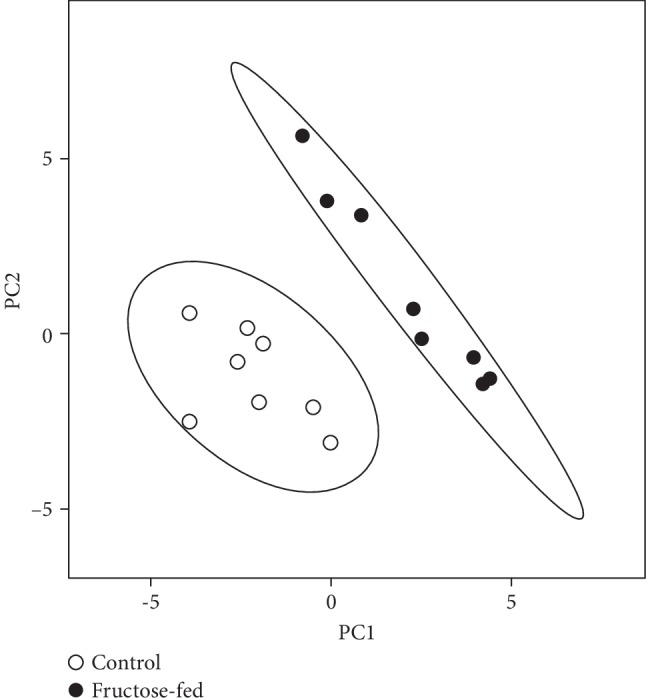
Principal component analysis (PCA) score plot. MS data expressed as mol% of membrane lipids were centered and normalized. Values for 8 independent experiments are shown for control and fructose-fed samples. Dashed lines display 95% confidence regions.

To characterize and elucidate the metabolic changes in the prediabetic heart induced by chronically applied fructose-rich diet, we performed high-performance, comprehensive shotgun MS-based lipidomic analyses from left ventricular whole membrane extracts. We have identified and quantified approximately 200 lipid molecular species encompassing 20 lipid classes (lipidomic data are summarized in Supplementary Lipid Table expressed either as lipid/protein or as mol% of membrane lipids or mol% of a given lipid class). Because the optimal physical state of the membrane is a prerequisite for proper functioning, in the following, we focus on membrane lipid compositional data. To obtain an overview, mol% of membrane lipid values was subjected to the nonsupervised multivariate statistics principal component analysis (PCA). The clear separation of the sample sets into two nonoverlapping clusters (Figure 7) indicates complex reshaping and metabolic rewiring of the whole lipidome due to fructose feeding. Examining these alterations in more detail and comparing the molecular species patterns for the control and fructose groups revealed 100 statistically significant differences (Supplementary Lipid Table).

**Figure 8:**
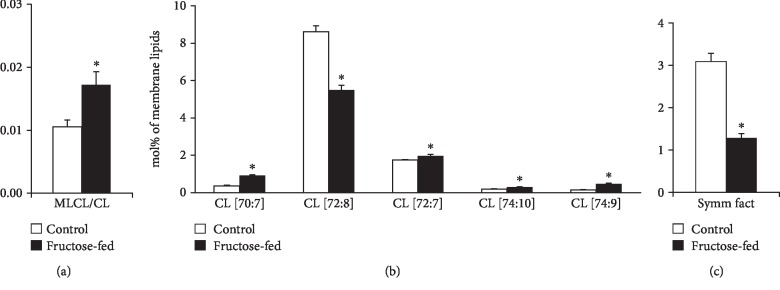
Defects in cardiolipin remodeling. (a) Monolysocardiolipin to cardiolipin ratio (MLCL/CL). (b) Changes in CL species due to fructose feeding. (c) CL symmetry factor calculated as the ratio of symmetric/asymmetric CL species. ESI-MS data are expressed as mol% of membrane lipids or calculated from the corresponding values and presented as means ± SEM (*n* = 8), ^∗^*p* < 0.05.

One of the most noteworthy changes can be connected to the CL remodeling system. It is known that under normal conditions, the levels of LPLs are kept low in general, and CL remodeling requires only trace amounts of MLCL [14]. Therefore, the significantly lowered level of matured CL in parallel with the significantly increased amount of MLCL (Supplementary Lipid Table), and consequently their markedly increased ratio in the membrane (MLCL/CL, Figure 8(a)), obviously report about an aberrant remodeling process in the fructose-fed group as compared to the controls. The ratio of MLCL/CL was found to be a more sensitive indicator than the level changes of CL and MLCL in Barth patients [58]. Furthermore, at the molecular species level, we detected pronounced loss of the most abundant homo-symmetric tetra18:2 species CL(72:8) (Figure 8(b)).

This was the most prominent change not only in the context of membrane composition but also when considering absolute values, i.e., the protein-normalized data displayed dramatic 45% decrease for CL(72:8) (from 28.5 to 15.7 nmol/protein mg; *p* < 0.05; Supplementary Lipid Table). This observation is in full agreement with other literature data obtained for either more severe diabetic and obesity models [59, 60] or recorded in a more similar early-stage fructose-induced type 2 diabetes study [61]. The loss in CL(72:8) was paralleled by elevations in practically all other asymmetric species independently on chain length and saturation for the fructose-fed animals as opposed to the normal chow diet (sum elevation from 2.82 to 4.35 mol% of membrane lipids, *p* < 0.05) (Figure 8(b)). This altogether resulted in a dramatic drop of the CL “symmetry” factor in the fructose group calculated as the ratio of symmetric/asymmetric species (Figure 8(c)). The major contributions to the increase in asymmetry derived from species which contain one non-18:2 acyl chain, i.e., from the 16:1 FA-containing CL(70:7; 16:1_18:2_18:2_18:2) species, from the CL(74:9) species whose major component is the CL(18:2_18:2_18:2_20:3) isobar, and from the CL(72:7) species corresponding to CL(18:1_18:2_18:2_18:2). This is in correspondence with the result observed in a fructose-induced early type 2 diabetic rat model [61] but differs from more severe mouse models of diabetes and insulin resistance/obesity. In the latter cases, defective cardiac CL remodeling resulted in depletion of 16:1 and enrichment of the highly unsaturated docosahexaenoic acid (DHA, 22:6 n-3) [17, 60], thereby essentially increasing the propensity of CL to peroxidation. In our study, the double bond index (DBI) of CL, a measure of unsaturation, did not change significantly (Supplementary Lipid Table). It can be partially due to the prediabetic nature of the model but also due to the sizeable difference in cardiac CL species composition between mouse and rat. Mouse cardiac CL contains essentially more DHA [61–63]. Therefore, it is more prone to ROS attack and peroxidation than that of the rat CL. Nevertheless, we have to mention that regarding fold increases of the individual CL species in the fructose-fed animals compared to the controls, the highest, ca. 10-fold elevations, was registered for DHA-containing CL species (78:12, 18:0_18:2-20:4_22:6 (major)) and (78:13, 18:1_18:2_20:4_22:6), although their levels barely reached the 0.1 mol% of total CL value even in the fructose group (Supplementary Lipid Table). It is important to note here that we could not detect oxidized lipid species either in CL or in other highly unsaturated and generally oxidation-prone lipid classes, such as plasmalogen phosphatidylethanolamine and phosphatidylserine. However, we could detect “asymmetry” defects already in the MLCL species profile; the major MLCL(54:6, tri18:2) species was found to be significantly reduced whereas the not only 18:2-containing precursors were markedly elevated (Supplementary Lipid [Supplementary-material supplementary-material-1]).

**Figure 9:**
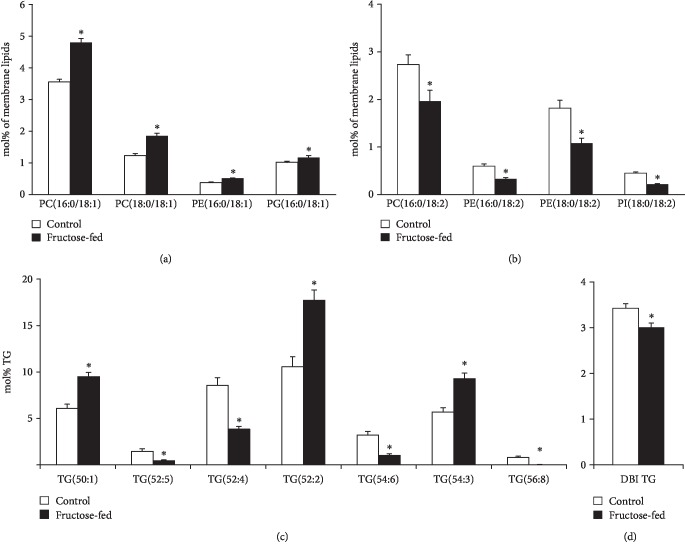
Early signs of de novo lipogenesis induction. (a) Changes in phospholipid species with 1 double bond. (b) Changes in phospholipid species with 2 double bonds. (c) Alterations in cardiac triglyceride (TG) species profile due to fructose feeding. (d) The double bond index calculated for TG. PC: phosphatidylcholine; PE: phosphatidylethanolamine; PG: phosphatidylglycerol; PI: phosphatidylinositol. ESI-MS data are expressed as mol% of membrane lipids or mol% of the specified lipid class and presented as means ± SEM (*n* = 8), ^∗^*p* < 0.05.

Another important feature of the lipidome alterations was the general increase in lipid species with sum double bond (db) = 1 that could be detected in all major membrane PL classes in the fructose group as opposed the control animals (sum of db = 1 9.3 vs. 6.9 mol% of membrane lipids, *p* < 0.05; Supplementary Lipid Table). The main contributors, which contain a saturated and a monounsaturated FA leg, were collected in Figure 9(a). PL species with db = 2 predominantly contained a saturated FA in *sn1* and a linoleoyl (18:2) group in *sn2* position of the glycerol backbone; these can serve as potential acyl donors for the formation of tetra18:2 CL for the tafazzin-catalyzed transacylation. In parallel with the elevation of species with db = 1, we detected significant depletion in species with db = 2 in fructose-fed animals (sum of db = 2 7.1 vs. 9.7 mol% of membrane lipids, *p* < 0.05; Supplementary Lipid Table); selected species are demonstrated in Figure 9(b).

Changes in PL molecular species with highly unsaturated acyl chains (db ≥ 4) showed fairly complex picture with several significant alterations including both elevations and decreases (Supplementary Lipid Table). It was reported that the loss of tafazzin enzymatic activity in a Barth syndrome mouse model also resulted in complex alterations of polyunsaturated PL species [64]. Therefore, it is conceivable to suggest that the intricate imbalance in polyunsaturated PL species alters the biophysical and signaling properties of the cardiac membrane.

Since we could not detect the elevation in serum triglyceride levels due to fructose feeding, it is not surprising that neither the total cardiac TG content changed significantly (Supplementary Lipid Table). However, the prominent species profile change of the TG pool is worth mentioning. The robust relative increase in species containing saturated and monounsaturated FAs, such as TG(50:1, 52:2, and 54:3), in parallel with significant reductions in more unsaturated species, e.g., TG(52:4, 54:6, and 56:8) (Figure 9(c)) altogether led to the decrease of the double bond index (DBI), i.e., increase in saturation for cardiac TG (Figure 9(d)). Cardiac TG saturation together with the monoene increase and 18:2 decrease in membrane PLs may indicate the upregulation of DNL leading to a shift in FA profile towards the augmentation of monounsaturated 18:1 (and 16:1) FAs.

**Figure 10:**
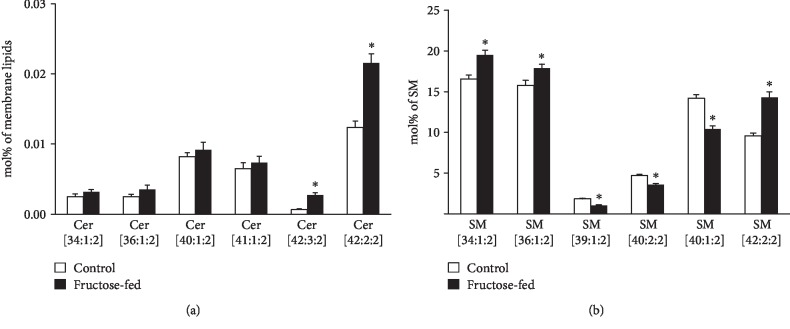
Modulation of the sphingolipid pool. (a) Alterations in ceramide (Cer) species. (b) Changes in the sphingomyelin (SM) species profile. ESI-MS data are expressed as mol% of membrane lipids or mol% of the specified lipid class and presented as means ± SEM (*n* = 8), ^∗^*p* < 0.05.

A further interesting aspect of the complex lipidome remodeling was the reshaping of the analyzed sphingolipid (SL) pool, ceramide (Cer) and sphingomyelin (SM). Cer has a central role in SL metabolism as well as it is known as a lipid mediator of the eukaryotic stress response. Its role is mostly associated with growth inhibition; the most studied being its function as a proapoptotic molecule [65]. Serum Cers have emerged as potential biomarkers of insulin resistance, diabetes, and heart disease, but also, muscle, liver, or adipose tissue Cers were shown to be associated with insulin resistance [66]. In our study, we measured small but significant elevation in total cardiac Cer at membrane lipid compositional level (approximately 30%; *p* < 0.05, Supplementary Lipid Table), which could be attributed almost exclusively to the increases in very-long-chain Cer-24 species Cer(42:2:2, d18:1/24:1 and 42:3:2, d18:2/24:1) (Figure 10(a)). It was reported that the nature of the acyl chain in Cers influences their contribution to the disease. Long-chain Cer-16 and Cer-18 often showed stronger associations with disease pathologies than very-long-chain Cer-24 [66]. Marchesini et al. reported that in confluent MCF-7 cells cell cycle arrest but not apoptosis was mediated by C24-Cer species [67]. It seems, therefore, that the alterations in Cer in our model might contribute to the observed cardiac dysfunction through changing the membrane biophysical properties rather than inducing sizeable apoptotic signaling. SM is the major structural mammalian SL which accumulates in liquid-ordered microdomains. Its total level showed only an increasing tendency in the membrane (*p* = 0.058), but its species compositions changed completely (Figure 10(b) and Supplementary Lipid Table). This may point to microdomain reorganization and hence, again, to the modulation of the membrane physical state and signaling properties due to fructose-rich diet.

Schlame et al. proposed that the acyl specificity of tafazzin arises from the physical properties of the lipid environment and is born out of a transacylation equilibrium in which the tissue-specific availability of FAs and the specific packing conditions of lipids are manifested [18]. In line with this proposal, our lipidomic data fully support the disturbance in tafazzin action in the prediabetic state as the very first event leading to defective CL structural uniformity and molecular symmetry. Nevertheless, several other options may contribute to the observed changes. These include the induction of phospholipase A_2_, which should increase the level of LPLs. It is known to happen in the diabetic state [68], and the registered increases in the relative levels of cardiac MLCL and LPE (Supplementary Lipid Table) in our prediabetic model might also reflect such an upregulation. In addition, the upregulation of the acyl-CoA:lyso-CL-acyltransferase that lacks preference for the linoleoyl group [69] as well as the downregulation of MLCL acyltransferase that specifically catalyzes the synthesis of tetra18:2 CL [70] also might play a role. However, these possibilities were reported for harsher conditions of later stage diabetes or well-developed oxidative stress induced by hyperthyroidism.

### 3.4. Proteomics

The complex changes in the heart detected by lipidomics at the metabolite level in the prediabetic state induced by chronic fructose feeding can be further mapped and complemented by alterations that occur at the protein level. Therefore, we performed comprehensive LC-MS-based proteomic analysis from left ventricular extracts.

Altogether, 1406 proteins were identified with at least two validate peptides from 1D-GE bands of pooled left ventricular samples. Using a spectral library built from those identifications, 802 proteins could be repeatedly quantified in individual samples. Seventy-five different proteins were significantly changed (*p* ≤ 0.05 and a minimum of 1.5-fold change) in fructose-fed rats compared to control animals. Out of these proteins, 49 were upregulated and 26 were downregulated. Gene ontology analysis based on subcellular localization revealed enrichments of proteins with significant changes in different cell compartments including mitochondria (*n* = 27), cytoplasm (*n* = 32), nucleus (*n* = 10), extracellular space (*n* = 8), lysosome (*n* = 3), and Golgi apparatus (*n* = 3) (Proteomic Table in the Supplementary Material). The relation of these proteins to different biochemical pathways was further analyzed in Reactome (https://www.reactome.org). The pathway analysis showed that most of the significantly altered proteins are related to metabolism (30 in pathways plus 9 interacting with some pathway proteins). Several metabolic pathways are affected, including the citric acid (TCA) cycle and respiratory electron transport (*n* = 10 + 3), metabolism of proteins (*n* = 9 + 11), lipids (*n* = 5 + 3), amino acids (*n* = 8), and carbohydrates (*n* = 6 + 1) (for a complete list, see the Supplementary Proteomic Table). Significantly altered proteins related to mitochondria, extracellular matrix, histones, oxidative stress, or apoptosis will be discussed in the following chapters.

### 3.5. Mitochondria

The proteomic analysis revealed changes in several proteins associated to various mitochondrial metabolic pathways due to fructose feeding (Table 6). These altered proteins can be coupled to the pyruvate dehydrogenase complex, electron transport chain, transport processes, and various metabolic pathways, such as the beta oxidation, tricarboxylic acid cycle, or amino acid metabolism. Pyruvate dehydrogenase complex plays a central role in the utilization of glucose as an energy source. Pyruvate dehydrogenase complex-related genes significantly decreased measured by proteomic analysis. In contrast, some beta oxidation-related proteins were increased. The first common point in the breakdown of glucose and fatty acids, citrate synthase, was also increased measured by proteomic analysis. These findings may suggest a shift to fatty acid utilization in cardiac tissue. We have also found both increased and decreased proteins related to the electron transport chain and amino acid metabolism.

### 3.6. Extracellular Matrix

We have found three extracellular matrix-related proteins to be significantly increased and another one to be decreased in the hearts of fructose-fed rats as assessed by proteomic analysis (Table 7). The increased proteins (prolargin, biglycan, and cathepsin D) may play a role in coping mechanism of the heart to prevent severe impairment. Prolargin was shown to be increased in a porcine model of ischemia/reperfusion injury [71]. Our group had previously shown that biglycan protects cardiomyocytes against hypoxia/reoxygenation injury [72, 73] and increases the expression of several proteins related to cardioprotection [74]. It has been also reported that myocardial cathepsin D upregulation induced by myocardial infarction protects against cardiac remodeling in mice [75]. Interestingly, galectin-1, known to have a protective role in cardiac homeostasis and postinfarction remodeling, is decreased in fructose-fed rats [76]. These results support the activation of adaptive mechanisms in the hearts of prediabetic rats.

### 3.7. Histones

Interestingly, expression of two histone proteins (core histone macro-H2A.1 and histone H1.5) was significantly increased in the hearts of fructose-fed rats. Histones are involved in packing the DNA in the nucleus, and mis-regulated histone expression is thought to lead to aberrant gene transcription by altering the chromatin structure [77].

### 3.8. Oxidative Stress and Apoptosis

Oxidative stress has a major role in the development of diabetic cardiomyopathy [7], and oxidative stress has been linked to the development of cardiac dysfunction [27, 78]. Moreover, elevated hydrogen peroxide production, elevated nitrotyrosine formation, and decreased cardiac function were observed in a prediabetes model induced by high-fat chow combined with a single low-dose STZ [6]. In our mild prediabetes model induced by fructose-enriched diet, there was no significant increase in the levels of the peroxidation product malondialdehyde in the serum or left ventricular tissue or in the cardiac level of the nitrooxidative marker 3-nitrotyrosine as compared to control values (Table 8).

However, we have detected by proteomics the increase of some oxidative stress-related enzymes from cardiac tissue, which may suggest an initial stage of oxidative stress that seems to be controlled by adaptive responses. We have found that alpha-aminoadipic semialdehyde dehydrogenase was increased in the hearts of fructose-fed rats (Table 5). Alpha-aminoadipic semialdehyde dehydrogenase protects cells from oxidative stress by metabolizing a number of lipid peroxidation-derived aldehydes [79, 80]. We have found that peroxiredoxin-6 was also increased in the fructose-fed group (Table 5). This enzyme catalyzes the reduction of hydrogen peroxide, short chain organic, fatty acid, and phospholipid hydroperoxides. It also has phospholipase activity and can therefore either reduce the oxidized sn2 fatty acyl group of phospholipids (peroxidase activity) or hydrolyze the sn2 ester bond of phospholipids (phospholipase activity). It plays a role in phospholipid homeostasis and in cell protection against oxidative stress by detoxifying peroxides [81]. Mitochondrial superoxide dismutase decreased in fructose-fed rats, and this result is consistent with the findings of Lappalainen et al. showing that SOD2 decreased in the kidney of STZ-induced diabetic rats [82].

**Figure 11:**
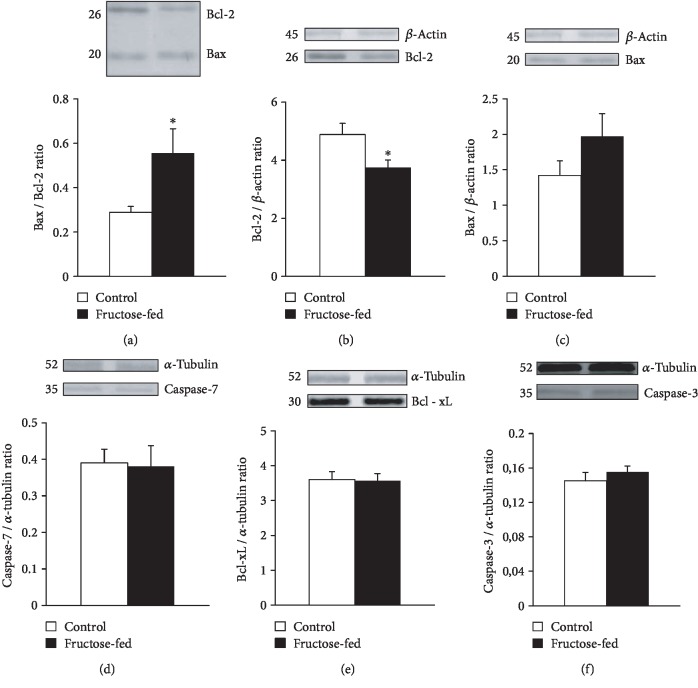
Western blot results at week 24: (a) Bax/Bcl-2 ratio, (b) Bcl-2/*β*-actin ratio, (c) Bax/*β*-actin ratio, (d) caspase-7/*α*-tubulin ratio, (e) Bcl-xL/*α*-tubulin ratio, and (f) caspase-3/*α*-tubulin ratio. Values are means ± SEM (*n* = 8), ^∗^*p* < 0.05.

Since increased apoptosis often contributes to cardiac dysfunction, in our present study, we also aimed to explore the effect of prediabetes on apoptosis in the heart. We assessed the expression of pro- and antiapoptotic proteins by western blot. Prediabetes did not affect the expression of proapoptotic caspase-7 and Bax in the left ventricles, while the antiapoptotic Bcl-2 was downregulated, and thereby, the Bax/Bcl-2 ratio was significantly increased in the fructose-fed group (Figure 11).

Similar to our results, a modest decrease in Bcl-2 has been shown recently in another model of prediabetes induced by a combination of high-fat diet and low-dose STZ injection [6]. In our present study, we also found in the proteomic results an increase in the antiapoptotic mitochondrial 3-ketoacyl-CoA thiolase in the hearts of fructose-fed rats (Table 5). We think that this alteration is part of the coping mechanism which protects the cardiomyocytes against apoptosis. Indeed, it is known that 3-ketoacyl-CoA thiolase abolishes BNIP3-mediated apoptosis and mitochondrial damage [83]. It was shown that 3-ketoacyl-CoA thiolase increased in the heart of STZ-induced diabetic mice [84]. The -3.4-fold decrease in the disulfide-isomerase protein level may also contribute to the suppression of apoptosis (Table 5) [85]. The upregulation of proapoptotic proteins and the downregulation of antiapoptotic proteins have already been described in a diabetic model in rodents [86, 87]. Our data suggest early dysregulation of pro- and antiapoptotic proteins in prediabetes; however, they do not show high induction of apoptosis. Furthermore, it is generally accepted that in type 1 and type 2 diabetes, the low levels of certain heat stress proteins (e.g., Hsp70 and Hsp27) and their impaired response to stress may contribute to the etiology of the disease [88]. It is important to note that in the prediabetic state, we could not detect any disruption of Hsps. Instead, the Hsp60 and alphaB-Crystallin levels were markedly elevated by 3.3- and 5.6-fold, respectively (Table 5).

## 4. Conclusion

**Figure 12:**
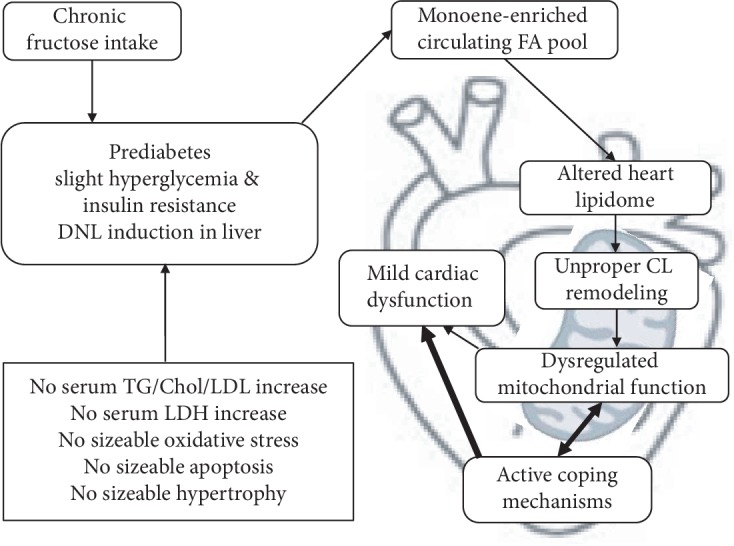
Summary of our findings.

This is the first comprehensive analysis of the effect of prediabetes on the lipidome and proteome of the heart and its relationship to impaired diastolic function in a nongenetic rodent model. In our present study, chronically applied fructose intake led to the development of a prediabetic condition characterized by slight hyperglycemia, glucose intolerance, and insulin resistance (Figure 12).

This prediabetic state likely caused slight DNL induction in the liver. DNL induction has the capacity to alter the circulating accessible fatty acid (FA) pool for lipid biosynthesis in other organs. Consequently, the cardiac lipidome has been altered. The change was found to be comprehensive, deep, and characteristic with main features of monoenoic FA enrichment, decrease in linoleic acid (18:2 FA), complex changes in highly polyunsaturated lipids, and reprofiling of sphingolipid species compositions. Recent large-scale findings highlighted that the lower risk of type 2 diabetes was strongly associated with higher 18:2 FA biomarker levels [89, 90]. Linoleic acid was able to alleviate the STZ-induced diabetic phenotype in mice by normalizing FA metabolism and desaturation and correcting glucose and insulin levels [91].

It is conceivable that the observed lipidomic changes ultimately altered the biophysical properties of membrane lipids, which, together with restricted substrate availability, led to unproper CL remodeling in cardiac mitochondria. Dysregulation of CL remodeling possibly contributed to the impairment of several mitochondrial processes, as it was assessed by proteomic analysis, and finally could result in mild cardiac dysfunction. As mentioned previously, Barth syndrome shares biochemical features, like increased CL molecular species heterogeneity and increased MLCL/CL ratio, with ischemia, hypothyroidism, heart failure, and aging [19, 92, 93]. Moreover, drastic CL remodeling was observed at early stages in type 1 and type 2 diabetic hearts [19]. Evidences from prospective cohort studies and randomized trials have demonstrated that high n-6 polyunsaturated FA (predominantly linoleic acid) intake plays an important role in the dietary prevention of cardiovascular diseases [94]. Altogether, it is well-established that the loss and defective remodeling of CL alone can provoke cardiac dysfunction but it is not sufficient to induce diabetes [95]. However, the deprivation of 18:2 caused by the fructose-induced overproduction of nonessential FAs can be an important contributor to the development of the disease. Moreover, as a consequence of the mitochondrial dysfunction, a vicious cycle can be initiated by ROS-induced damage to mitochondrial components [96] at the transition from the prediabetic to the diabetic stage.

Our data show that at this very early stage of prediabetes there was no sizeable oxidative stress or apoptosis in the heart. Instead, several active coping mechanisms were activated against the harmful consequences of fructose feeding including the upregulation of enzymes responsible for the removal of lipid peroxidation products and upregulation of mitochondrial Hsp60.

Taken together, our study evidences that at the prediabetic stage there are no clinically accessible signs to declare a disease because the generally investigated serum parameters do not report about lipotoxicity, cell damage, or substantial hyperglycemia. Nevertheless, the results presented here clearly demonstrate that the risk for progression of diabetes and cardiovascular disease is silently present in the guise of a complex cardiac lipid metabolic imbalance and altered proteomic pattern. Prediabetes might represent a transient, reversible, “decision-making” state in that process. The nature of our nongenetic model implies that improper food intake must persist chronically; the longer you apply the higher the risk. Therefore, early intervention is important to prevent the transition from prediabetes to more severe disease stages.

## Figures and Tables

**Table 1:** Isolated organ weights and isolated organ weight to body weight ratios at week 24 in both control and fructose-fed rats. Values are mean ± SEM (*n* = 8), ^∗^*p* < 0.05.

	Control	Fructose-fed	*p* value
Heart weight (mg)	1726 ± 79	1527 ± 71	0.083
Liver weight (mg)	13292 ± 538	12262 ± 467	0.170
Pancreas weight (mg)	1093 ± 111	1101 ± 467	0.951
Heart (mg)/body weight (g)	3.22 ± 0.14	3.36 ± 0.09	0.422
Liver (mg)/body weight (g)	22.7 ± 0.2	27.0 ± 0.4^∗^	≤0.001
Pancreas (mg)/body weight (g)	2.04 ± 0.19	2.43 ± 0.11	0.095

**Table 2:** Parameters measured in serum collected at week 24 in both control and fructose-fed rats. Values are mean ± SEM (*n* = 8), ^∗^*p* < 0.05.

	Control	Fructose-fed	*p* value
Serum triglyceride (mmol/L)	0.96 ± 0.12	0.94 ± 0.20	0.938
Serum cholesterol (mmol/L)	1.78 ± 0.14	1.61 ± 0.11	0.359
LDL (mmol/L)	0.45 ± 0.05	0.43 ± 0.08	0.830
HDL (mmol/L)	0.86 ± 0.08	0.76 ± 0.07	0.335
ALAT (U/L)	38.63 ± 3.35	35.00 ± 4.08	0.500
ASAT (U/L)	77.88 ± 4.05	73.29 ± 5.74	0.517
CK (U/L)	263 ± 46	245 ± 45	0.776
CKMB (U/L)	352 ± 75	256 ± 33	0.264
LDH (U/L)	334.86 ± 63.77	272.50 ± 37.36	0.437
Cl (mmol/L)	102.50 ± 0.78	102.63 ± 0.82	0.914
K (mmol/L)	6.33 ± 0.36	5.95 ± 0.57	0.588
Na (mmol/L)	141.63 ± 0.60	141.38 ± 0.78	0.802

**Table 3:** Left ventricular morphological and functional parameters examined by echocardiography at week 24 in both control and fructose-fed rats. Values are mean ± SEM (*n* = 8), ^∗^*p* < 0.05.

Parameter (unit)	View/mode	Control	Fructose-fed	*p* value
Left ventricle morphology				
Anterior wall thickness in systole (mm)	Short axis/MM	3.59 ± 0.14	3.47 ± 0.12	0.506
Anterior wall thickness in diastole (mm)	Short axis/MM	2.35 ± 0.04	2.00 ± 0.10^∗^	0.012
Inferior wall thickness in systole (mm)	Short axis/MM	3.38 ± 0.18	3.41 ± 0.07	0.871
Inferior wall thickness in diastole (mm)	Short axis/MM	2.20 ± 0.11	2.01 ± 0.08	0.167
Posterior wall thickness in systole (mm)	Long axis/MM	3.33 ± 0.30	3.13 ± 0.19	0.596
Posterior wall thickness in diastole (mm)	Long axis/MM	2.22 ± 0.14	1.96 ± 0.11	0.155
Septal wall thickness in systole (mm)	Long axis/MM	3.88 ± 0.18	3.55 ± 0.20	0.239
Septal wall thickness in diastole (mm)	Long axis/MM	2.33 ± 0.10	2.05 ± 0.17	0.169
Left ventricular end-diastolic diameter (mm)	Long axis/MM	6.85 ± 0.24	6.85 ± 0.21	0.996
Left ventricular end-systolic diameter (mm)	Long axis/MM	3.07 ± 0.16	3.37 ± 0.20	0.270
Left ventricular end-diastolic volume (*μ*L)	4CH	100.3 ± 20.6	90.3 ± 11.7	0.681
Left ventricular end-systolic volume (*μ*L)	4CH	39.23 ± 9.04	36.23 ± 6.03	0.787
Left ventricular morphology/heart weight				
Anterior wall thickness in systole (mm/g)	Short axis/MM	1.96 ± 0.11	2.28 ± 0.06^∗^	0.021
Anterior wall thickness in diastole (mm/g)	Short axis/MM	1.27 ± 0.07	1.32 ± 0.06	0.596
Inferior wall thickness in systole (mm/g)	Short axis/MM	1.96 ± 0.06	2.20 ± 0.11	0.079
Inferior wall thickness in diastole (mm/g)	Short axis/MM	1.38 ± 0.10	1.34 ± 0.10	0.795
Posterior wall thickness in systole (mm/g)	Long axis/MM	1.96 ± 0.20	2.05 ± 0.09	0.665
Posterior wall thickness in diastole (mm/g)	Long axis/MM	1.21 ± 0.12	1.29 ± 0.06	0.559
Septal wall thickness in systole (mm/g)	Long axis/MM	2.28 ± 0.14	2.34 ± 0.11	0.735
Septal wall thickness in diastole (mm/g)	Long axis/MM	1.36 ± 0.06	1.73 ± 0.37	0.348
Left ventricular end-diastolic diameter (mm/g)	Long axis/MM	3.86 ± 0.23	4.56 ± 0.27	0.067
Left ventricular end-systolic diameter (mm/g)	Long axis/MM	1.81 ± 0.10	2.26 ± 0.20	0.082
Left ventricle function				
E/A	4CH	1.21 ± 0.07	1.03 ± 0.02^∗^	0.015
Ejection fraction (%)	4CH	60.96 ± 3.38	62.83 ± 2.32	0.666
Fractional shortening (%)	Short axis/MM	49.57 ± 3.82	54.00 ± 3.60	0.414
MV E velocity (m/s)	4CH	0.81 ± 0.05	0.73 ± 0.06	0.358
MV A velocity (m/s)	4CH	0.71 ± 0.07	0.82 ± 0.06	0.260
Heart rate (1/min)	4CH	346.0 ± 12.9	349.6 ± 6.8	0.819

**Table 4:** Parameters measured by working heart perfusion at week 24 in both control and fructose-fed rats. Values are mean ± SEM (*n* = 7‐8).

	Control	Fructose-fed	*p* value
Aortic flow (mL)	46.6 ± 3.7	37.4 ± 2.4	0.065
Coronary flow (mL)	24.6 ± 1.1	21.9 ± 1.2	0.121
Max dp/dt (mmHg/s)	5975 ± 330	6063 ± 212	0.832
Min dp/dt (mmHg/s)	−3577 ± 222	−4090 ± 237	0.138
Aortic diastolic pressure (mmHg)	45.7 ± 1.7	42.1 ± 1.3	0.120
Aortic systolic pressure (mmHg)	110.3 ± 2.6	116.2 ± 1.5	0.121
LVDP (mmHg)	137.5 ± 6.0	139.5 ± 4.3	0.803
Heart rate (1/min)	279 ± 14	263 ± 24	0.554

**Table 5:** Alteration of selected cardiac proteins in fructose-fed rats by proteomic analysis. Values are expressed as fold change and *p* value.

Protein names	Gene names	Fold change	*p* value
Alpha B crystallin	Cryab	5.56	0.047
3-ketoacyl-CoA thiolase (mitochondrial)	Acaa2	4.56	0.005
Alpha-aminoadipic semialdehyde dehydrogenase	Aldh7a1	4.34	≤0.001
60 kDa heat shock protein (mitochondrial)	Hspd1	3.37	0.010
Myosin 6	MYH6	3.06	0.003
Peroxiredoxin-6	Prdx6	2.87	0.001
Superoxide dismutase [Mn] (mitochondrial)	Sod2	-1.82	0.033
Protein disulfide-isomerase	P4hb	-3.40	0.001

**Table 6:** Alteration of selected mitochondrial proteins in fructose-fed rats by proteomic analysis. Values are expressed as fold change and *p* value.

Protein names	Gene names	Fold change	*p* value
Pyruvate dehydrogenase complex			
Dihydrolipoyllysine-residue acetyltransferase component of pyruvate dehydrogenase complex (mitochondrial)	Dlat	-3.47	0.022
Dihydrolipoamide acetyltransferase component of pyruvate dehydrogenase complex	Dbt	-9.53	0.039
Electron transport chain			
ETF-ubiquinone oxidoreductase (mitochondrial)	Etfdh	3.13	0.004
NADH dehydrogenase [ubiquinone] 1 alpha subcomplex subunit 2	Ndufa2	2.06	0.047
NADH-ubiquinone oxidoreductase chain 4	Mt-Nd4	1.59	0.007
Cytochrome c, testis-specific	Cyct	1.56	0.011
Cytochrome b-c1 complex subunit Rieske (mitochondrial)	Uqcrfs1	-1.95	0.001
NADH dehydrogenase [ubiquinone] 1 alpha subcomplex subunit 10 (mitochondrial)	Ndufa10	-3.15	0.038
Amino acid metabolism			
3-Hydroxyisobutyryl-CoA hydrolase (mitochondrial)	Hibch	5.44	0.042
Isovaleryl-CoA dehydrogenase (mitochondrial)	Ivd	-1.90	0.004
Methylcrotonoyl-CoA carboxylase beta chain (mitochondrial)	Mccc2	-2.18	0.046
Transport function			
ADP/ATP translocase 1	Slc25a4	2.47	0.005
Voltage-dependent anion-selective channel protein 3	Vdac3	2.17	0.018
MICOS complex subunit Mic60	Immt	1.58	0.003
Beta oxidation			
Enoyl CoA hydratase domain-containing 2	Echdc2	1.50	0.018
Electron transfer flavoprotein subunit alpha (mitochondrial)	Etfa	-1.94	0.001
Other			
Malic enzyme	Me3	4.27	0.022
Citrate synthase	Cs	3.14	≤0.001
Enoyl-[acyl-carrier-protein] reductase (mitochondrial)	Mecr	-1.61	0.029
Prohibitin-2	Phb2	-1.65	0.001

**Table 7:** Alteration of selected extracellular matrix proteins in fructose-fed rats by proteomic analysis. Values are expressed as fold change and *p* value.

Protein names	Gene names	Fold change	*p* value
Extracellular matrix			
Prolargin	Prelp	5.23	0.001
Biglycan	Bgn	1.91	0.018
Cathepsin D	Ctsd	1.52	0.001
Galectin-1	Lgals1	-3.23	0.005

**Table 8:** Oxidative stress markers measured in serum and heart tissue in both control and fructose-fed rats. Values are mean ± SEM (*n* = 8).

	Control	Fructose-fed	*p* value
Serum malondialdehyde (nmol/mg protein)	4.77 ± 0.43	4.03 ± 0.49	0.274
Cardiac malondialdehyde (nmol/mg protein)	1.27 ± 0.16	1.47 ± 0.22	0.482
3-Nitrotyrosine (nmol/mg)	190 ± 6.0	221 ± 20	0.164

## Data Availability

All data used to support the findings of this study are included within the article or the supplementary information file.

## Supplementary Materials

Supplementary Materials Supplementary methods for more detailed descriptions of the methodologies of lipidomics and proteomics. Supplementary Figure 1: cardiolipin (CL) maturation. Supplementary Figure 2: alterations in monolysocardiolipin (MLCL) species composition due to fructose-rich diet. Then, the Supplementary Tables are given to provide detailed data related to lipidomic and proteomic analyses. The Supplementary Tables include the following: lipid compositional data (mol% of membrane lipids), species composition of individual lipid classes (mol% of individual classes), lipid/protein values (nmol/mg), double bonds (db) and double bond index (DBI), protein quantification, and Reactome pathway analysis.
