# Supplementary material for: Prediabetes Induced by Fructose-Enriched Diet Influences Cardiac Lipidome and Proteome and Leads to Deterioration of Cardiac Function prior to the Development of Excessive Oxidative Stress and Cell Damage
Source: Oxid Med Cell Longev. 2019 Dec 9;2019:3218275. doi: 10.1155/2019/3218275 (PMC6925817; doi:10.1155/2019/3218275)
Supplement: Supplementary Materials — Supplementary methods for more detailed descriptions of the methodologies of lipidomics and proteomics. Supplementary Figure 1: cardiolipin (CL) maturation. Supplementary Figure 2: alterations in monolysocardiolipin (MLCL) species composition due to fructose-rich diet. Then, the Supplementary Tables are given to provide detailed data related to lipidomic and proteomic analyses. The Supplementary Tables include the following: lipid compositional data (mol% of membrane lipids), species composition of individual lipid classes (mol% of individual classes), lipid/protein values (nmol/mg), double bonds (db) and double bond index (DBI), protein quantification, and Reactome pathway analysis. [file 3218275.f1.pdf]

Oxidative Medicine and Cellular Longevity –

Special Issue: Cardiolipin and Mitochondria-Targeted Antioxidants in Oxidative Stress, Disease, and Aging

Prediabetes induced by fructose-enriched diet influences cardiac lipidome and proteome and leads to deterioration of cardiac function prior to the development of excessive oxidative stress and cell damage

[1,2]Gergő Szűcs*†, [1,2]Andrea Sója†, [3]Mária Péter†, [1,2]Márta Sárközy, [2,4]Bella Bruszel, [5]Andrea Siska, [5]Imre Földesi, [2,4]Zoltán Szabó, [2,4]Tamás Janáky, [3]László Vígh, [3]Gábor Balogh[#], [1,2]Tamás Csont[#,*]

[1]Metabolic Diseases and Cell Signaling Group, Department of Biochemistry, Faculty of Medicine, University of Szeged, Szeged H-6720, Hungary

[2]Interdisciplinary Centre of Excellence, University of Szeged, Szeged H-6720, Hungary

[3]Institute of Biochemistry, Biological Research Center of the Hungarian Academy of Sciences, Szeged H-6726, Hungary

[4]Institute of Medical Chemistry, Faculty of Medicine, University of Szeged, Szeged H-6720, Hungary.

[5]Department of Laboratory Medicine, Faculty of Medicine, University of Szeged, Szeged H-6720, Hungary

Working Title: Cardiolipin in prediabetic cardiac dysfunction.

*Correspondence

Gergő Szűcs;

szucs.gergo@med.u-szeged.hu

Tamás Csont;

Csont.tamas@med.u-szeged.hu

†Equal contributors

[#]Equal senior contributors

Keywords: fructose diet, diastolic dysfunction, cardiolipin, lipidome, proteome, oxidative stress, apoptosis, adaptation

Supplementary Methods

Lipidomics

Lipidomic analyses were performed on an LTQ-Orbitrap Elite instrument (Thermo Fisher Scientific) equipped with a robotic nanoflow ion source TriVersa NanoMate (Advion BioSciences) using chips with the diameter of 5.5-µm spraying nozzles. The ion source was controlled by Chipsoft 8.3.1 software. The ionization voltages were +1.3 kV and −1.9 kV in positive and negative mode, respectively, and the back-pressure was set at 1 psi in both modes. The temperature of the ion transfer capillary was 330 °C. Acquisitions were performed at the mass resolution $R_{m/z\ 400}$ = 240,000.

Phosphatidylcholine (PC, diacyl and PC-P, alkenyl-acyl), lysophosphatidylcholine (LPC), sphingomyelin (SM), giacylglycerol (DG) and triacylglycerol (TG) were detected and quantified using the positive ion mode, while phosphatidylethanolamine (PE, diacyl and PE-Pl, alkenyl-acyl), phosphatidylinositol (PI), phosphatidylserine (PS), phosphatidic acid (PA), phosphatidylglycerol (PG), cardiolipin (CL), the corresponding lyso derivatives LPE, LPI, LPS, LPG and monolysocardiolipin (MLCL) as well as ceramide (Cer) and ganglioside GM3 were detected and quantified using the negative ion mode.

For quantification, tenfold dilution was prepared from the lipid extracts, from which 8 µl was further diluted with 142 µl infusion solvent mixture (chloroform:methanol:iso-propanol 1:2:1, by vol.; extract concentration upon injection ca. 100 ng ww/µl) containing an internal standard mix (71 pmol PC d31-16:0/18:1, 5 pmol SM d18:1/17:0, 5 pmol DG 15:0-d7-15:0, 19 pmol TG 15:0-18:1-d7-15:0, and 25 pmol PE d31-16:0/18:1, 11 pmol PI d31-16:0/18:1, 19 pmol PS d31-16:0/18:1, 2.5 pmol PG d31-16:0/18:1, 1 pmol PA d31-16:0/18:1, 1.5 pmol CL 56:0, 2 pmol Cer d18:1/17:0, and 5 pmol GM3 d3-d18:1/18:0. Next, the mixture was halved, and 5% dimethylformamide (additive for the negative ion mode) or 3 mM ammonium chloride (additive for the positive ion mode) were added to the split sample halves. The mass tolerance was 3 ppm.

For fragmentation analyses, HCD collision energy values were determined for each lipid class. Data-dependent MS/MS fragmentation experiments were performed based on mass lists from survey scans. Data files generated by LipidXplorer queries were further processed by in-house Excel macros.

Lipid classes and species were annotated according to the lipid classification systems [38], [39]. For glycerolipids, in sum formulas, e.g., PC(34:2), the total numbers of carbons followed by double bonds for all chains are indicated. The Lipid class(FA1_FA2) format specifies the structures of the fatty acyl (FA) side chains, e.g., PC(16:0_18:2), whereas the Lipid class(sn1/sn2) format specifies the side chain regiochemistry, too, e.g., PC(16:0/18:2). For sphingolipids, the sum formula, e.g., Cer(42:2:2), specifies first the total number of carbons in the long chain base and FA moiety then the sum of double bonds in the long chain base and the FA moiety followed by the sum of hydroxyl groups in the long chain base and the FA moiety. In the detailed formula, first the number of hydroxyl groups in the long chain base

(e.g., “d” for the two hydroxyls of sphingosine), the number of carbon atoms and then the number of double bonds are indicated followed by the N-acyl chain composition, such as Cer(d18:1/24:1).

The double bond index (DBI) was calculated for the major membrane phospholipids and TG as Σ(mol % of unsaturated fatty acids in the given lipid class x number of double bonds of each unsaturated fatty acid)/100.

Proteomics

Protein extraction

Approximately 30 mg of powered left ventricular tissue samples were homogenized in lysate buffer (contained 2 % SDS and 0.1 M DTT in 0.1 Tris solution). The homogenized samples were incubated at 98 °C for 5 min. Proteins were precipitated by addition of methanol/chloroform mixture (4:1) and were resuspended in 8 M urea. The total protein contents were determined using BCA (Thermo) protocol.

SDS-PAGE

SDS-PAGE was performed to pre-fractionate proteins for building a more comprehensive protein library of the heart proteome. For gel separation 20 μg protein from a pooled sample of all individual powered left ventricular tissue samples subjected to SDS-PAGE analysis on a 12 % SDS polyacrylamide gel (8*8 cm) using one-dimensional separation. Electrophoresis was performed on 80 V for 30 minutes to introduce the sample into the 5 % stacking gel. After that, the electrophoresis was carried out on 100 V for 2 hours. The protein bands were stained using Coomassie BrilliantBlue G-250 solution. After fixation (with 30 % ethanol, 10 % acetic acid) the gel was cut to 19 pieces, and the proteins were ’in-gel’ digested by trypsin.

’In gel’ digestion

The excised gel bands were cut into small pieces and destained in 0.1 M NH_4HCO_3 (pH = 8.0), dehydrated with acetonitrile and dried using Speed-Vac. Alkylation and reduction of proteins were performed using 10 mM DTT and 55 mM IAA solutions. After dehydration, the gel pieces were rehydrated with 12.5 ng/µl trypsin (Promega, Madison, WI, US) solution (in 25 mM NH_4HCO_3 pH = 8.0) and incubated overnight at 37 °C. Peptides were extracted with 5 % aqueous formic acid, and twice with 60 % acetonitrile in 1 % formic acid (20 min each). Samples were dried with vacuum centrifugation before LC/MS analysis.

’In solution’ digestion

Proteins were digested using the following protocol: all individual powdered left ventricular tissue samples containing 20 μg protein were diluted to 10 μl with 0.1 M NH_4HCO_3 (pH = 8.0) buffer; 12 μl 0.1 % RapiGest SF (Waters) and 2 μl 55 mM DTT solution was added and kept at 60 °C for 30 minutes

to unfold and reduce proteins. A volume of 2 μl 200 mM IAA solution was added to alkylate the proteins which were kept for an additional 30 min in the dark at room temperature. The samples were digested with trypsin (Promega, the enzyme/protein ratio: 0.4/1) for 3h at 37 °C. The digestion was stopped by addition of 1 μl of concentrated hydrochloric acid.

A pooled sample was created by mixing equal amounts of all digested samples to extend the protein library built from the gel separated pooled sample.

LC-MS analysis

The separation of the digested samples was carried out on a nanoAcquity UPLC, (Waters, Milford, MA, US) using Waters ACQUITY UPLC M-Class Peptide C18 (130 Å, 1,78 µm, 75 µm*250mm) column with 90 min gradient. Eluents were water (A), and acetonitrile (B) containing 0.1 V/V % formic acid and the separation of the peptide mixture was performed at 45 °C with 0.35 μl/min flow rate using an optimized nonlinear LC gradient (3 %-40 % B). The LC was coupled to a high-resolution Q Exactive Plus quadrupole-orbitrap hybrid mass spectrometer (Thermo Scientific, Waltham, Massachusetts, US). In measurements to build the protein library, the mass spectrometer was operated in DDA (Data Dependent Acquisition) mode.

The ,,fast" method from Kelstrup[1] was used for DDA acquisition. The mass spectrometer was operated in the data-dependent mode to automatically switch between full scan MS and MS/MS acquisition. The full scan was performed between 300 – 1750 m/z with 70000 resolution at 200 m/z. The MS/MS scans were carried out with 17500 resolution after the accumulation of ions to a 1×10^6 target value based on predictive AGC from the previous full scan. The 12 most intense multiply charged ions were fragmented in the octopole collision cell by normalized HCD collision energy (25 %). The spray voltage was set to 1.8 kV, and the capillary was heated to 275 °C. The MS/MS ion selection threshold was set to 1×10^5 counts.

The quantitative measurements of digested individual samples were performed in DIA (Data Independent Acquisition) mode. The survey scan for DIA method operated with 35000 resolution. The full scan was performed between 380 to 1020 m/z. The AGC target was set to $5*10^6$ or 120 ms maximum injection time. In the 400-1000 m/z region 22 m/z wide overlapping windows were acquired at 17500 resolution (AGC target: $3*10^6$ or 100 ms injection time, normalized collision energy: 30 for charge 2).

Data analysis

The DDA spectra were processed with SearchGUI Version 1.6.1.6. analysis software[2] using four of the built-in search engines (Andromeda, Comet, OMSSA, X!Tandem). The minimal peptide length was set to 6. Search criteria included carbamidomethylation of cysteine as a fixed modification and

oxidation of methionine and acetyl (protein N-terminus) as variable modifications. The mass tolerance for the precursor was 10 ppm and for the fragment ions was 0.15 Da. The DDA files were searched against rat reference proteome from the UniProt database. Trypsin was defined as enzyme with a maximum of 2 missed cleavages allowed. Protein identifications were validated in Peptideshaker[3] 3.65 using 1 % FDR limits both on peptide and protein level. The quantitative analysis was performed in Encyclopedia[4] 0.81. The statistical evaluations were carried out using Perseus software on text reports exported from Encyclopedia software. MSstats 3.73[5] was used to determine the minimal acceptable fold change for our sample size (n=8) at a statistical power of 80%. This minimal fold change was found to be 1.5, and this limit, together with a maximum of p=0.05 of Welch's t-test were used as a criterion for significant changes.

Subcellular localization of signifiacantly changing proteins was assigned based on Gene Onthology (www.ebi.ac.uk/QuickGO/).

Supplementary proteomics tables contain measured peptide intensities and assignments, quantification data of proteins with $p \leq 0.05$ in t-test, and pathway assignment of significantly changing proteins from Reactome (www.reactome.org) after assignment to human genes, for higher annotation coverage.

Supplementary references

1. Kelstrup CD, Young C, Lavallee R, et al. Optimized fast and sensitive acquisition methods for shotgun proteomics on a quadrupole orbitrap mass spectrometer. J Proteome Res. 2012;11:3487-3497.
2. Barsnes H, Vaudel M. SearchGUI: A Highly Adaptable Common Interface for Proteomics Search and de Novo Engines. J Proteome Res. 2018;17:2552-2555. doi:10.1021/acs.jproteome.8b00175.
3. Vaudel M, Burkhart JM, Zahedi RP, et al. PeptideShaker enables reanalysis of MS-derived proteomics data sets. Nat Biotechnol. 2015;33:22-24. doi:10.1038/nbt.3109.
4. Searle BC, Pino LK, Egertson JD, et al. Chromatogram libraries improve peptide detection and quantification by data independent acquisition mass spectrometry. Nat Commun. 2018;9:5128. doi:10.1038/s41467-018-07454-w.
5. Choi M, Chang CY, Clough T, et al. MSstats: an R package for statistical analysis of quantitative mass spectrometry-based proteomic experiments. Bioinformatics. 2014;30:2524-2526.

Supplementary Figures

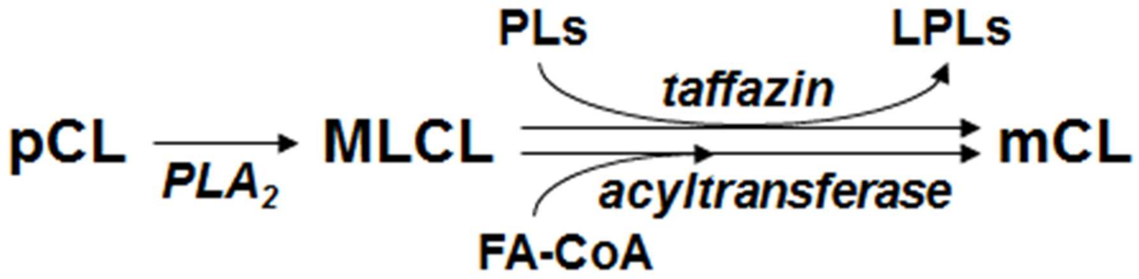

Supplementary Figure 1: Cardiolipin (CL) maturation. After its initial biosynthesis, premature CL (pCL) undergoes intensive remodeling processes to produce matured CL (mCL). First, the removal of a single acyl chain is executed by a calcium-independent phospholipase A_2 (PLA_2) to produce monolysocardiolipin (MLCL). Reacylation is catalyzed either by CoA-dependent acyltransferases or the CoA-independent, reversible phospholipid (PL)-lysophospholipid (LPL) transacylase taffazin.

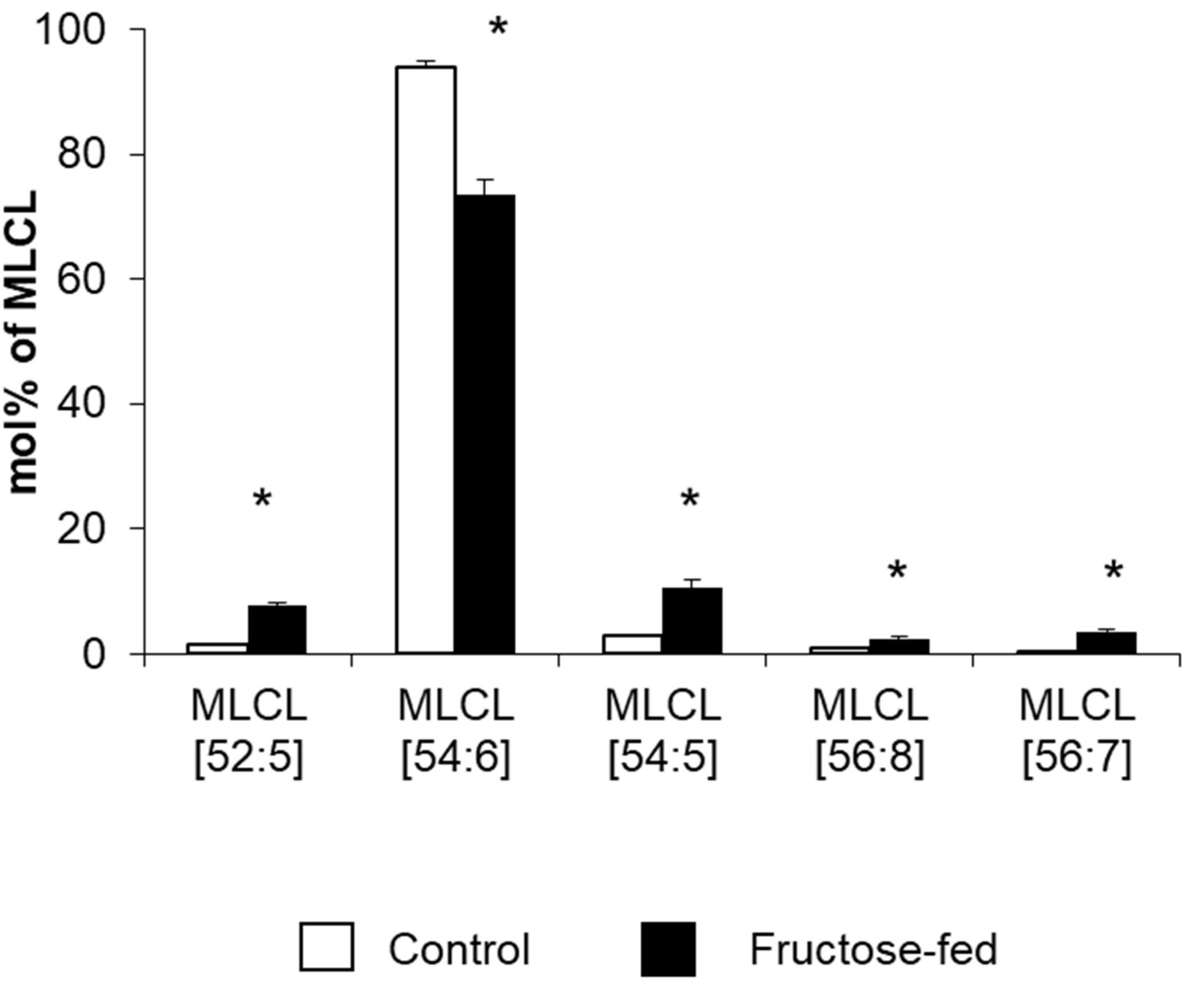

Supplementary Figure 2: Alterations in monolysocardiolipin (MLCL) species composition due to fructose-rich diet. ESI-MS data are expressed as mol% of MLCL or calculated from the corresponding values and presented as means ± SEM (n=8), *$p<0.05$.

Supplementary Tables

Lipid compositional data (mol% of membrane lipids)

Lipid compositional data

Data are expressed as mol% of membrane lipids and given as mean ± SEM, n = 8
****p<0.05 corresponding to false discovery rate <0.05; FC, fold change***

	Control		*Fructose*				
Species name	avg	*SEM*	avg	*SEM*	*p*		FC
PC(32:1)	0.132	*0.012*	0.202	*0.014*	1.7E-03	*	*1.53*
PC(32:0)	0.947	*0.037*	0.921	*0.042*	6.5E-01		*0.97*
PC(34:3)	0.064	*0.007*	0.062	*0.008*	8.2E-01		*0.96*
PC(34:2)	2.731	*0.208*	1.960	*0.231*	2.6E-02	*	*0.72*
PC(34:1)	3.556	*0.082*	4.803	*0.121*	6.4E-07	*	*1.35*
PC(34:0)	0.100	*0.006*	0.082	*0.005*	2.8E-02	*	*0.82*
PC(36:5)	0.164	*0.015*	0.194	*0.005*	7.3E-02		*1.18*
PC(36:4)	8.764	*0.173*	8.739	*0.256*	9.4E-01		*1.00*
PC(36:3)	1.111	*0.068*	1.354	*0.201*	2.7E-01		*1.22*
PC(36:2)	3.412	*0.246*	3.045	*0.398*	4.5E-01		*0.89*
PC(36:1)	1.233	*0.061*	1.859	*0.077*	1.8E-05	*	*1.51*
PC(38:6)	1.786	*0.146*	1.342	*0.051*	1.2E-02	*	*0.75*
PC(38:5)	1.977	*0.077*	2.618	*0.067*	2.0E-05	*	*1.32*
PC(38:4)	15.081	*0.464*	14.759	*0.547*	6.6E-01		*0.98*
PC(40:8)	0.127	*0.013*	0.091	*0.004*	2.2E-02	*	*0.72*
PC(40:7)	0.156	*0.005*	0.170	*0.006*	8.7E-02		*1.09*
PC(40:6)	0.560	*0.030*	0.548	*0.043*	8.3E-01		*0.98*
PC(40:5)	0.479	*0.028*	0.571	*0.054*	1.5E-01		*1.19*
PC(40:4)	0.190	*0.009*	0.181	*0.012*	6.0E-01		*0.96*
**PC**	**42.569**	***0.382***	**43.501**	***0.414***	1.2E-01		*1.02*
PC-P(34:1)	0.006	*0.002*	0.007	*0.002*	6.6E-01		*1.17*
PC-P(34:0)	0.010	*0.001*	0.016	*0.001*	7.8E-03	*	*1.59*
PC-P(36:4)	0.379	*0.100*	0.378	*0.066*	9.9E-01		*1.00*
PC-P(38:5)	0.071	*0.019*	0.079	*0.015*	7.4E-01		*1.11*
PC-P(38:4)	0.033	*0.007*	0.030	*0.005*	7.0E-01		*0.90*
**PC-P**	**0.500**	***0.127***	**0.510**	***0.087***	9.5E-01		*1.02*
LPC(16:0)	0.058	*0.003*	0.065	*0.004*	2.1E-01		*1.12*
LPC(18:2)	0.001	*0.000*	0.001	*0.000*	4.3E-01		*0.69*
LPC(18:1)	0.005	*0.001*	0.011	*0.001*	1.5E-03	*	*2.12*
LPC(18:0)	0.096	*0.005*	0.097	*0.008*	8.9E-01		*1.01*
LPC(20:4)	0.013	*0.003*	0.011	*0.002*	5.8E-01		*0.82*
**LPC**	**0.173**	***0.009***	**0.184**	***0.013***	4.7E-01		*1.07*
LPE(16:0)	0.004	*0.000*	0.007	*0.002*	9.4E-02		*1.88*
LPE(18:1)	0.032	*0.001*	0.035	*0.002*	1.7E-01		*1.10*
LPE(18:0)	0.027	*0.001*	0.033	*0.001*	2.0E-02	*	*1.19*
**LPE**	**0.063**	***0.002***	**0.075**	***0.004***	1.4E-02	*	*1.19*
PE(34:2)	0.598	*0.046*	0.331	*0.027*	2.1E-04	*	*0.55*
PE(34:1)	0.379	*0.021*	0.518	*0.014*	1.1E-04	*	*1.36*
PE(36:4)	1.430	*0.074*	1.735	*0.076*	1.2E-02	*	*1.21*
PE(36:3)	0.621	*0.055*	0.424	*0.060*	2.9E-02	*	*0.68*

PE(36:2)	1.816	*0.165*	1.076	*0.111*	2.3E-03	*	*0.59*
PE(36:1)	0.207	*0.012*	0.250	*0.012*	2.2E-02	*	*1.21*
PE(38:6)	1.831	*0.074*	1.425	*0.139*	2.2E-02	*	*0.78*
PE(38:5)	1.550	*0.065*	2.602	*0.049*	3.7E-09	*	*1.68*
PE(38:4)	8.254	*0.364*	8.801	*0.198*	2.1E-01		*1.07*
PE(38:3)	0.020	*0.004*	0.080	*0.013*	7.1E-04	*	*4.04*
PE(40:7)	0.489	*0.016*	0.592	*0.022*	2.2E-03	*	*1.21*
PE(40:6)	4.225	*0.234*	4.115	*0.330*	7.9E-01		*0.97*
PE(40:5)	2.407	*0.231*	3.138	*0.296*	7.2E-02		*1.30*
PE(40:4)	0.429	*0.014*	0.347	*0.012*	4.4E-04	*	*0.81*
**PE**	**24.256**	***0.350***	**25.435**	***0.616***	1.2E-01		*1.05*
PE-P(34:2)	0.146	*0.006*	0.095	*0.005*	8.8E-06	*	*0.65*
PE-P(34:1)	0.076	*0.004*	0.113	*0.008*	8.1E-04	*	*1.48*
PE-P(36:4)	3.144	*0.146*	3.365	*0.129*	2.8E-01		*1.07*
PE-P(36:2)	0.066	*0.003*	0.053	*0.003*	7.6E-03	*	*0.80*
PE-P(38:6)	1.141	*0.068*	0.727	*0.034*	9.2E-05	*	*0.64*
PE-P(38:5)	2.257	*0.170*	2.464	*0.163*	3.9E-01		*1.09*
PE-P(38:4)	1.928	*0.112*	1.456	*0.068*	2.8E-03	*	*0.76*
PE-P(40:7)	0.371	*0.017*	0.322	*0.015*	4.4E-02	*	*0.87*
PE-P(40:6)	0.398	*0.023*	0.293	*0.011*	9.2E-04	*	*0.74*
PE-P(40:5)	0.194	*0.010*	0.170	*0.005*	4.9E-02	*	*0.87*
PE-P(40:4)	0.116	*0.003*	0.096	*0.002*	2.4E-05	*	*0.82*
**PE-P**	**9.839**	***0.417***	**9.153**	***0.370***	2.4E-01		*0.93*
LPI(18:1)	0.017	*0.001*	0.018	*0.001*	3.5E-01		*1.09*
LPI(18:0)	0.004	*0.000*	0.004	*0.000*	9.5E-01		*0.99*
LPI(20:4)	0.004	*0.000*	0.004	*0.001*	7.2E-01		*0.93*
**LPI**	**0.025**	***0.001***	**0.026**	***0.002***	5.4E-01		*1.04*
PI(34:2)	0.042	*0.004*	0.026	*0.002*	1.8E-03	*	*0.62*
PI(34:1)	0.004	*0.003*	0.030	*0.007*	5.4E-03	*	*7.70*
PI(36:4)	0.117	*0.004*	0.128	*0.004*	8.0E-02		*1.10*
PI(36:3)	0.079	*0.005*	0.067	*0.008*	2.8E-01		*0.86*
PI(36:2)	0.452	*0.022*	0.218	*0.016*	5.5E-07	*	*0.48*
PI(36:1)	0.032	*0.002*	0.045	*0.004*	1.0E-02	*	*1.40*
PI(38:6)	0.019	*0.001*	0.011	*0.001*	1.0E-05	*	*0.58*
PI(38:5)	0.177	*0.005*	0.263	*0.013*	2.5E-05	*	*1.49*
PI(38:4)	3.452	*0.036*	3.411	*0.079*	6.4E-01		*0.99*
PI(40:6)	0.035	*0.002*	0.024	*0.002*	5.9E-04	*	*0.69*
PI(40:5)	0.039	*0.004*	0.032	*0.002*	1.3E-01		*0.83*
PI(40:4)	0.021	*0.002*	0.017	*0.001*	2.4E-02	*	*0.78*
**PI**	**4.469**	***0.038***	**4.274**	***0.063***	1.9E-02	*	*0.96*
LPS(18:1)	0.012	*0.001*	0.013	*0.001*	3.3E-01		*1.10*
**LPS**	**0.012**	***0.001***	**0.013**	***0.001***	3.3E-01		*1.10*
PS(34:1)	0.005	*0.001*	0.011	*0.001*	9.9E-05	*	*2.15*
PS(36:2)	0.125	*0.009*	0.096	*0.007*	1.9E-02	*	*0.77*
PS(36:1)	0.168	*0.004*	0.194	*0.008*	1.1E-02	*	*1.16*
PS(38:6)	0.006	*0.001*	0.004	*0.001*	7.8E-02		*0.64*
PS(38:5)	0.008	*0.001*	0.013	*0.001*	5.6E-04	*	*1.67*
PS(38:4)	0.377	*0.010*	0.345	*0.007*	1.7E-02	*	*0.92*
PS(38:3)	0.042	*0.001*	0.059	*0.004*	1.2E-03	*	*1.41*

PS(38:2)	0.003	*0.000*	0.005	*0.000*	2.2E-03	*	*1.63*
PS(40:7)	0.004	*0.001*	0.009	*0.001*	1.7E-03	*	*2.16*
PS(40:6)	1.007	*0.055*	0.955	*0.041*	4.6E-01		*0.95*
PS(40:5)	0.677	*0.054*	0.896	*0.045*	7.5E-03	*	*1.32*
PS(40:4)	0.296	*0.006*	0.245	*0.006*	5.1E-05	*	*0.83*
PS(40:3)	0.022	*0.007*	0.031	*0.004*	2.9E-01		*1.42*
**PS**	**2.739**	***0.036***	**2.863**	***0.049***	6.0E-02		*1.05*
LPG(18:2)	0.001	*0.000*	0.000	*0.000*	1.5E-01		*0.42*
LPG(18:1)	0.001	*0.000*	0.002	*0.000*	3.5E-03	*	*1.92*
**LPG**	**0.002**	***0.000***	**0.002**	***0.000***	4.3E-01		*1.20*
PG(32:1)	0.003	*0.001*	0.006	*0.001*	1.0E-03	*	*2.22*
PG(34:2)	0.172	*0.011*	0.105	*0.004*	7.2E-05	*	*0.61*
PG(34:1)	1.018	*0.036*	1.174	*0.058*	3.8E-02	*	*1.15*
PG(36:4)	0.040	*0.002*	0.029	*0.002*	4.6E-04	*	*0.73*
PG(36:3)	0.052	*0.003*	0.052	*0.002*	1.0E+00		*1.00*
PG(36:2)	0.142	*0.008*	0.109	*0.006*	7.8E-03	*	*0.77*
PG(36:1)	0.047	*0.004*	0.047	*0.002*	9.5E-01		*1.01*
PG(38:6)	0.013	*0.001*	0.005	*0.001*	8.4E-04	*	*0.37*
PG(38:5)	0.010	*0.001*	0.015	*0.002*	9.8E-03	*	*1.52*
PG(38:4)	0.023	*0.001*	0.023	*0.001*	9.9E-01		*1.00*
PG(40:8)	0.007	*0.001*	0.001	*0.000*	4.0E-04	*	*0.10*
PG(40:7)	0.011	*0.001*	0.009	*0.001*	1.6E-01		*0.79*
PG(40:6)	0.011	*0.001*	0.017	*0.002*	4.8E-03	*	*1.57*
**PG**	**1.548**	***0.047***	**1.591**	***0.059***	5.8E-01		*1.03*
PA(34:2)	0.011	*0.001*	0.010	*0.001*	5.7E-01		*0.93*
PA(34:1)	0.037	*0.002*	0.046	*0.003*	3.0E-02	*	*1.26*
PA(36:4)	0.006	*0.001*	0.004	*0.001*	3.3E-01		*0.78*
PA(36:3)	0.003	*0.001*	0.005	*0.001*	2.6E-01		*1.35*
PA(36:2)	0.013	*0.002*	0.015	*0.002*	5.1E-01		*1.15*
PA(36:1)	0.001	*0.000*	0.001	*0.000*	5.6E-01		*1.27*
PA(38:5)	0.003	*0.001*	0.003	*0.001*	7.0E-01		*1.20*
**PA**	**0.073**	***0.007***	**0.085**	***0.006***	2.4E-01		*1.16*
CL(68:6)	0.024	*0.002*	0.064	*0.003*	3.4E-08	*	*2.62*
CL(68:5)	0.005	*0.001*	0.017	*0.002*	1.9E-04	*	*3.63*
CL(70:7)	0.358	*0.046*	0.908	*0.040*	3.4E-07	*	*2.54*
CL(70:6)	0.026	*0.002*	0.118	*0.026*	3.1E-03	*	*4.60*
CL(70:5)	0.013	*0.002*	0.027	*0.004*	8.8E-03	*	*2.13*
CL(70:4)	0.008	*0.001*	0.015	*0.002*	1.1E-02	*	*1.84*
CL(72:9)	0.067	*0.003*	0.062	*0.003*	2.8E-01		*0.93*
CL(72:8)	8.615	*0.306*	5.481	*0.268*	2.1E-06	*	*0.64*
CL(72:7)	1.751	*0.021*	1.961	*0.084*	3.0E-02	*	*1.12*
CL(72:5)	0.001	*0.000*	0.003	*0.000*	5.3E-03	*	*2.58*
CL(74:11)	0.003	*0.001*	0.018	*0.003*	5.8E-04	*	*5.17*
CL(74:10)	0.193	*0.013*	0.289	*0.032*	1.4E-02	*	*1.50*
CL(74:9)	0.149	*0.013*	0.474	*0.027*	3.5E-08	*	*3.19*
CL(74:8)	0.037	*0.003*	0.043	*0.010*	5.7E-01		*1.17*
CL(76:12)	0.080	*0.010*	0.118	*0.019*	9.3E-02		*1.47*
CL(76:11)	0.082	*0.009*	0.160	*0.025*	1.1E-02	*	*1.96*
CL(76:10)	0.025	*0.002*	0.049	*0.005*	1.3E-03	*	*1.95*
CL(78:13)	0.001	*0.000*	0.012	*0.003*	2.2E-03	*	*10.73*
CL(78:12)	0.001	*0.000*	0.008	*0.002*	6.6E-04	*	*10.57*

**CL**	**11.437**	***0.244***	**9.827**	***0.164***	8.3E-05	*	*0.86*
MLCL(52:5)	0.002	*0.000*	0.013	*0.002*	1.2E-05	*	*7.66*
MLCL(52:4)	0.000	*0.000*	0.001	*0.000*	2.0E-02	*	*11.21*
MLCL(54:6)	0.112	*0.009*	0.120	*0.009*	5.3E-01		*1.07*
MLCL(54:5)	0.004	*0.000*	0.019	*0.004*	8.2E-04	*	*5.30*
MLCL(54:4)	0.000	*0.000*	0.001	*0.000*	5.4E-02		*6.64*
MLCL(56:8)	0.001	*0.000*	0.004	*0.001*	2.8E-02	*	*4.27*
MLCL(56:7)	0.000	*0.000*	0.006	*0.001*	1.3E-04	*	*12.89*
MLCL(58:10)	0.000	*0.000*	0.002	*0.001*	5.6E-02		*5.16*
MLCL(58:9)	0.000	*0.000*	0.002	*0.001*	6.1E-02		*8.02*
**MLCL**	**0.119**	***0.010***	**0.167**	***0.018***	3.4E-02	*	*1.40*
SM(34:2:2)	0.004	*0.001*	0.003	*0.001*	5.7E-01		*0.83*
SM(34:1:2)	0.313	*0.013*	0.385	*0.011*	9.3E-04	*	*1.23*
SM(36:2:2)	0.031	*0.003*	0.030	*0.003*	7.8E-01		*0.96*
SM(36:1:2)	0.297	*0.013*	0.354	*0.014*	9.6E-03	*	*1.19*
SM(38:2:2)	0.057	*0.004*	0.045	*0.004*	5.5E-02		*0.80*
SM(38:1:2)	0.220	*0.009*	0.212	*0.007*	4.7E-01		*0.96*
SM(39:2:2)	0.003	*0.000*	0.001	*0.000*	3.1E-03	*	*0.29*
SM(39:1:2)	0.035	*0.002*	0.020	*0.002*	9.6E-05	*	*0.59*
SM(40:2:2)	0.088	*0.003*	0.071	*0.003*	2.7E-03	*	*0.80*
SM(40:1:2)	0.267	*0.008*	0.205	*0.007*	3.3E-05	*	*0.77*
SM(41:2:2)	0.038	*0.004*	0.032	*0.003*	2.3E-01		*0.85*
SM(41:1:2)	0.107	*0.006*	0.097	*0.004*	2.0E-01		*0.91*
SM(42:2:2)	0.181	*0.006*	0.283	*0.016*	2.6E-05	*	*1.57*
SM(41:2:3)	0.012	*0.004*	0.011	*0.003*	9.1E-01		*0.95*
SM(42:1:2)	0.232	*0.008*	0.225	*0.007*	5.1E-01		*0.97*
**SM**	**1.886**	***0.030***	**1.976**	***0.031***	5.8E-02		*1.05*
Cer(34:1:2)	0.002	*0.000*	0.003	*0.000*	2.0E-01		*1.27*
Cer(36:1:2)	0.003	*0.000*	0.004	*0.001*	1.8E-01		*1.40*
Cer(38:1:2)	0.002	*0.000*	0.003	*0.000*	9.6E-02		*1.50*
Cer(40:2:2)	0.001	*0.000*	0.001	*0.000*	8.6E-01		*1.06*
Cer(40:1:2)	0.008	*0.001*	0.009	*0.001*	4.5E-01		*1.12*
Cer(41:2:2)	0.001	*0.000*	0.001	*0.000*	1.7E-01		*1.58*
Cer(41:1:2)	0.007	*0.001*	0.007	*0.001*	5.0E-01		*1.13*
Cer(42:3:2)	0.001	*0.000*	0.003	*0.000*	4.6E-05	*	*4.21*
Cer(42:2:2)	0.012	*0.001*	0.022	*0.001*	6.0E-05	*	*1.74*
Cer(42:1:2)	0.021	*0.001*	0.024	*0.001*	8.9E-02		*1.15*
**Cer**	**0.057**	***0.003***	**0.077**	***0.005***	3.5E-03	*	*1.34*
GM3(34:1:2)	0.010	*0.001*	0.013	*0.002*	2.3E-01		*1.30*
GM3(36:1:2)	0.031	*0.004*	0.046	*0.001*	5.1E-03	*	*1.49*
GM3(38:1:2)	0.073	*0.003*	0.065	*0.002*	4.9E-02	*	*0.90*
GM3(40:1:2)	0.055	*0.002*	0.042	*0.003*	4.5E-03	*	*0.78*
GM3(42:2:2)	0.036	*0.006*	0.043	*0.005*	4.2E-01		*1.19*
GM3(42:1:2)	0.029	*0.005*	0.032	*0.002*	5.1E-01		*1.12*
**GM3**	**0.233**	***0.005***	**0.242**	***0.010***	4.2E-01		*1.04*
DG(34:2)	0.029	*0.009*	0.041	*0.014*	5.0E-01		*1.40*
DG(34:1)	0.114	*0.016*	0.154	*0.030*	2.6E-01		*1.35*
DG(36:4)	0.030	*0.004*	0.018	*0.004*	4.2E-02	*	*0.59*
DG(36:3)	0.031	*0.005*	0.032	*0.015*	9.4E-01		*1.04*

DG(36:2)	0.098	*0.032*	0.111	*0.032*	7.7E-01		*1.14*
DG(38:5)	0.010	*0.003*	0.013	*0.003*	5.4E-01		*1.26*
DG(38:4)	0.176	*0.018*	0.174	*0.015*	9.4E-01		*0.99*
**DG**	**0.488**	***0.062***	**0.543**	***0.108***	6.6E-01		*1.11*
TG(50:5)	0.003	*0.001*	0.000	*0.000*	1.7E-01		*0.13*
TG(50:4)	0.057	*0.015*	0.033	*0.008*	1.8E-01		*0.59*
TG(50:3)	0.166	*0.057*	0.096	*0.051*	3.8E-01		*0.58*
TG(50:2)	0.438	*0.120*	0.377	*0.128*	7.3E-01		*0.86*
TG(50:1)	0.251	*0.065*	0.334	*0.103*	5.1E-01		*1.33*
TG(50:0)	0.021	*0.001*	0.023	*0.001*	4.2E-01		*1.06*
TG(52:5)	0.066	*0.022*	0.019	*0.007*	6.2E-02		*0.28*
TG(52:4)	0.378	*0.117*	0.137	*0.042*	7.4E-02		*0.36*
TG(52:3)	0.755	*0.203*	0.551	*0.182*	4.7E-01		*0.73*
TG(52:2)	0.463	*0.139*	0.669	*0.229*	4.5E-01		*1.44*
TG(52:1)	0.059	*0.006*	0.058	*0.011*	9.3E-01		*0.98*
TG(52:0)	0.007	*0.001*	0.008	*0.001*	2.8E-01		*1.18*
TG(54:7)	0.012	*0.005*	0.001	*0.000*	5.0E-02	*	*0.08*
TG(54:6)	0.129	*0.041*	0.032	*0.009*	3.9E-02	*	*0.25*
TG(54:5)	0.391	*0.100*	0.233	*0.048*	1.8E-01		*0.60*
TG(54:4)	0.363	*0.096*	0.272	*0.092*	5.0E-01		*0.75*
TG(54:3)	0.246	*0.071*	0.349	*0.120*	4.7E-01		*1.42*
TG(54:2)	0.056	*0.006*	0.060	*0.015*	7.8E-01		*1.08*
TG(56:8)	0.026	*0.005*	0.001	*0.001*	9.0E-05	*	*0.05*
TG(56:7)	0.055	*0.007*	0.028	*0.006*	1.3E-02	*	*0.50*
TG(56:6)	0.075	*0.009*	0.058	*0.013*	2.6E-01		*0.77*
TG(56:5)	0.058	*0.006*	0.070	*0.012*	3.8E-01		*1.20*
TG(56:4)	0.014	*0.003*	0.013	*0.005*	8.4E-01		*0.92*
TG(56:3)	0.010	*0.003*	0.011	*0.004*	8.1E-01		*1.13*
TG(58:9)	0.011	*0.002*	0.001	*0.000*	7.6E-04	*	*0.11*
TG(58:8)	0.022	*0.004*	0.006	*0.002*	2.8E-03	*	*0.28*
TG(58:7)	0.016	*0.004*	0.013	*0.004*	6.2E-01		*0.83*
TG(58:6)	0.011	*0.003*	0.011	*0.004*	9.8E-01		*0.99*
TG(58:5)	0.002	*0.001*	0.002	*0.001*	7.9E-01		*1.20*
TG(58:4)	0.000	*0.000*	0.000	*0.000*	1.7E-01		*0.29*
**TG**	**4.160**	***0.982***	**3.469**	***1.053***	6.4E-01		*0.83*
**Class composition**							
PC	42.569	*0.382*	43.501	*0.414*	1.2E-01		*1.02*
PC-P	0.500	*0.127*	0.510	*0.087*	9.5E-01		*1.02*
LPC	0.173	*0.009*	0.184	*0.013*	4.7E-01		*1.07*
LPE	0.063	*0.002*	0.075	*0.004*	1.4E-02	*	*1.19*
PE	24.256	*0.350*	25.435	*0.616*	1.2E-01		*1.05*
PE-P	9.839	*0.417*	9.153	*0.370*	2.4E-01		*0.93*
LPI	0.025	*0.001*	0.026	*0.002*	5.4E-01		*1.04*
PI	4.469	*0.038*	4.274	*0.063*	1.9E-02	*	*0.96*
LPS	0.012	*0.001*	0.013	*0.001*	3.3E-01		*1.10*
PS	2.739	*0.036*	2.863	*0.049*	6.0E-02		*1.05*
LPG	0.002	*0.000*	0.002	*0.000*	4.3E-01		*1.20*
PG	1.548	*0.047*	1.591	*0.059*	5.8E-01		*1.03*
PA	0.073	*0.007*	0.085	*0.006*	2.4E-01		*1.16*
CL	11.437	*0.244*	9.827	*0.164*	8.3E-05	*	*0.86*
MLCL	0.119	*0.010*	0.167	*0.018*	3.4E-02	*	*1.40*
SM	1.886	*0.030*	1.976	*0.031*	5.8E-02		*1.05*
Cer	0.057	*0.003*	0.077	*0.005*	3.5E-03	*	*1.34*
GM3	0.233	*0.005*	0.242	*0.010*	4.2E-01		*1.04*
DG	0.488	*0.062*	0.543	*0.108*	6.6E-01		*1.11*
TG	4.160	*0.982*	3.469	*1.053*	6.4E-01		*0.83*

Species composition of individual lipid classes (mol% of individulal classes)

Species composition of individual lipid classes

Data are expressed as mol% of the given lipid class and presented as mean ± SEM, n = 8, *p<0.05

	Control		*Fructose*			
Species name	avg	*SEM*	avg	*SEM*	*p*	
PC(32:1)	0.310	*0.025*	0.465	*0.031*	1.6E-03	*
PC(32:0)	2.223	*0.074*	2.120	*0.102*	4.3E-01	
PC(34:3)	0.150	*0.015*	0.141	*0.019*	7.1E-01	
PC(34:2)	6.423	*0.500*	4.487	*0.506*	1.7E-02	*
PC(34:1)	8.357	*0.198*	11.049	*0.303*	3.2E-06	*
PC(34:0)	0.235	*0.012*	0.188	*0.011*	1.3E-02	*
PC(36:5)	0.385	*0.031*	0.447	*0.010*	8.1E-02	
PC(36:4)	20.580	*0.280*	20.114	*0.682*	5.4E-01	
PC(36:3)	2.611	*0.160*	3.097	*0.447*	3.2E-01	
PC(36:2)	8.032	*0.609*	6.966	*0.876*	3.3E-01	
PC(36:1)	2.898	*0.148*	4.273	*0.171*	2.9E-05	*
PC(38:6)	4.182	*0.310*	3.086	*0.116*	5.2E-03	*
PC(38:5)	4.644	*0.179*	6.019	*0.154*	4.3E-05	*
PC(38:4)	35.418	*0.986*	33.944	*1.270*	3.7E-01	
PC(40:8)	0.298	*0.029*	0.210	*0.009*	1.2E-02	*
PC(40:7)	0.367	*0.011*	0.391	*0.014*	2.0E-01	
PC(40:6)	1.314	*0.069*	1.264	*0.107*	7.0E-01	
PC(40:5)	1.127	*0.069*	1.319	*0.133*	2.2E-01	
PC(40:4)	0.446	*0.024*	0.419	*0.031*	4.9E-01	
PC	100.000	*0.000*	100.000	*0.000*		
PC-P(34:1)	1.295	*0.143*	1.301	*0.203*	9.8E-01	
PC-P(34:0)	4.249	*1.861*	3.980	*0.870*	9.0E-01	
PC-P(36:4)	74.512	*0.882*	73.649	*0.836*	4.9E-01	
PC-P(38:5)	12.698	*1.682*	15.036	*0.593*	2.1E-01	
PC-P(38:4)	7.246	*0.352*	6.035	*0.394*	3.8E-02	*
PC-P	100.000	*0.000*	100.000	*0.000*		
LPC(16:0)	33.535	*0.913*	35.132	*0.641*	1.7E-01	
LPC(18:2)	0.479	*0.083*	0.324	*0.157*	4.0E-01	
LPC(18:1)	3.180	*0.443*	6.384	*0.836*	4.4E-03	*
LPC(18:0)	55.206	*1.110*	52.418	*1.322*	1.3E-01	
LPC(20:4)	7.600	*1.783*	5.742	*1.064*	3.9E-01	
LPC	100.000	*0.000*	100.000	*0.000*		
LPE(16:0)	6.223	*0.762*	9.233	*1.859*	1.6E-01	
LPE(18:1)	50.502	*1.984*	47.111	*1.948*	2.4E-01	
LPE(18:0)	43.274	*1.611*	43.656	*1.602*	8.7E-01	
LPE	100.000	*0.000*	100.000	*0.000*		
PE(34:2)	2.454	*0.168*	1.316	*0.125*	8.8E-05	*
PE(34:1)	1.568	*0.095*	2.047	*0.087*	2.3E-03	*
PE(36:4)	5.882	*0.257*	6.824	*0.265*	2.3E-02	*
PE(36:3)	2.557	*0.220*	1.703	*0.265*	2.6E-02	*
PE(36:2)	7.454	*0.624*	4.297	*0.511*	1.6E-03	*

PE(36:1)	0.854	*0.051*	0.988	*0.061*	1.1E-01	
PE(38:6)	7.557	*0.303*	5.544	*0.427*	1.8E-03	*
PE(38:5)	6.418	*0.331*	10.267	*0.285*	4.3E-07	*
PE(38:4)	33.982	*1.230*	34.699	*0.902*	6.5E-01	
PE(38:3)	0.082	*0.019*	0.321	*0.057*	1.4E-03	*
PE(40:7)	2.019	*0.077*	2.327	*0.063*	7.8E-03	*
PE(40:6)	17.411	*0.915*	16.049	*0.943*	3.2E-01	
PE(40:5)	9.990	*1.016*	12.252	*0.952*	1.3E-01	
PE(40:4)	1.772	*0.065*	1.365	*0.027*	4.4E-05	*
PE	100.000	*0.000*	100.000	*0.000*		
PE-P(34:2)	1.512	*0.100*	1.041	*0.048*	8.3E-04	*
PE-P(34:1)	0.779	*0.039*	1.232	*0.073*	8.4E-05	*
PE-P(36:4)	31.923	*0.560*	36.788	*0.385*	4.9E-06	*
PE-P(36:2)	0.683	*0.055*	0.575	*0.024*	9.4E-02	
PE-P(38:6)	11.646	*0.607*	8.010	*0.429*	2.4E-04	*
PE-P(38:5)	22.807	*1.085*	26.748	*0.760*	1.0E-02	*
PE-P(38:4)	19.616	*0.829*	15.898	*0.268*	7.8E-04	*
PE-P(40:7)	3.807	*0.190*	3.545	*0.197*	3.6E-01	
PE-P(40:6)	4.056	*0.170*	3.234	*0.179*	5.0E-03	*
PE-P(40:5)	1.972	*0.045*	1.871	*0.083*	3.0E-01	
PE-P(40:4)	1.198	*0.053*	1.058	*0.050*	7.8E-02	
PE-P	100.000	*0.000*	100.000	*0.000*		
LPI(18:1)	67.413	*2.155*	70.379	*2.902*	4.3E-01	
LPI(18:0)	14.574	*1.420*	14.012	*1.083*	7.6E-01	
LPI(20:4)	18.013	*1.489*	15.609	*2.509*	4.2E-01	
LPI	100.000	*0.000*	100.000	*0.000*		
PI(34:2)	0.950	*0.084*	0.620	*0.045*	3.8E-03	*
PI(34:1)	0.087	*0.064*	0.703	*0.176*	5.4E-03	*
PI(36:4)	2.622	*0.103*	3.004	*0.088*	1.4E-02	*
PI(36:3)	1.760	*0.116*	1.585	*0.209*	4.8E-01	
PI(36:2)	10.099	*0.453*	5.119	*0.379*	7.3E-07	*
PI(36:1)	0.720	*0.045*	1.053	*0.088*	4.7E-03	*
PI(38:6)	0.419	*0.023*	0.253	*0.015*	3.0E-05	*
PI(38:5)	3.958	*0.118*	6.169	*0.348*	3.1E-05	*
PI(38:4)	77.254	*0.579*	79.781	*1.092*	6.0E-02	
PI(40:6)	0.787	*0.045*	0.568	*0.031*	1.2E-03	*
PI(40:5)	0.866	*0.078*	0.755	*0.038*	2.2E-01	
PI(40:4)	0.476	*0.037*	0.391	*0.011*	4.3E-02	*
PI	100.000	*0.000*	100.000	*0.000*		
LPS(18:1)	100.000	*0.000*	100.000	*0.000*		
LPS	100.000	*0.000*	100.000	*0.000*		
PS(34:1)	0.191	*0.019*	0.395	*0.034*	1.3E-04	*
PS(36:2)	4.594	*0.352*	3.370	*0.256*	1.4E-02	*
PS(36:1)	6.122	*0.156*	6.795	*0.328*	8.6E-02	
PS(38:6)	0.205	*0.027*	0.123	*0.024*	4.0E-02	*
PS(38:5)	0.279	*0.024*	0.446	*0.027*	4.4E-04	*
PS(38:4)	13.763	*0.336*	12.064	*0.265*	1.4E-03	*
PS(38:3)	1.519	*0.056*	2.051	*0.143*	3.8E-03	*
PS(38:2)	0.117	*0.017*	0.180	*0.009*	5.9E-03	*

PS(40:7)	0.158	*0.024*	0.328	*0.039*	2.3E-03	*
PS(40:6)	36.750	*1.931*	33.379	*1.372*	1.8E-01	
PS(40:5)	24.687	*1.911*	31.253	*1.325*	1.4E-02	*
PS(40:4)	10.809	*0.262*	8.547	*0.182*	5.5E-06	*
PS(40:3)	0.804	*0.268*	1.069	*0.134*	3.9E-01	
PS	100.000	*0.000*	100.000	*0.000*		
LPG(18:2)	41.679	*7.007*	16.420	*3.268*	5.6E-03	*
LPG(18:1)	58.321	*7.007*	83.580	*3.268*	5.6E-03	*
LPG	100.000	*0.000*	100.000	*0.000*		
PG(32:1)	0.178	*0.045*	0.380	*0.034*	3.0E-03	*
PG(34:2)	11.194	*0.822*	6.669	*0.407*	2.2E-04	*
PG(34:1)	65.727	*0.997*	73.609	*1.023*	7.6E-05	*
PG(36:4)	2.570	*0.111*	1.799	*0.056*	2.3E-05	*
PG(36:3)	3.346	*0.153*	3.300	*0.220*	8.7E-01	
PG(36:2)	9.135	*0.386*	6.971	*0.596*	8.7E-03	*
PG(36:1)	2.977	*0.200*	2.960	*0.129*	9.4E-01	
PG(38:6)	0.820	*0.105*	0.282	*0.060*	5.6E-04	*
PG(38:5)	0.638	*0.048*	0.917	*0.072*	6.2E-03	*
PG(38:4)	1.474	*0.060*	1.433	*0.060*	6.4E-01	
PG(40:8)	0.493	*0.108*	0.046	*0.013*	1.1E-03	*
PG(40:7)	0.739	*0.063*	0.558	*0.073*	8.2E-02	
PG(40:6)	0.708	*0.059*	1.075	*0.073*	1.6E-03	*
PG	100.000	*0.000*	100.000	*0.000*		
PA(34:2)	15.658	*1.662*	12.193	*1.121*	1.1E-01	
PA(34:1)	51.381	*2.474*	54.854	*2.071*	3.0E-01	
PA(36:4)	7.403	*1.053*	5.130	*0.716*	9.6E-02	
PA(36:3)	4.460	*0.484*	5.318	*0.502*	2.4E-01	
PA(36:2)	16.790	*1.680*	17.241	*0.900*	8.2E-01	
PA(36:1)	1.047	*0.287*	1.259	*0.291*	6.1E-01	
PA(38:5)	3.260	*1.026*	4.007	*1.130*	6.3E-01	
PA	100.000	*0.000*	100.000	*0.000*		
CL(68:6)	0.215	*0.024*	0.650	*0.033*	4.4E-08	*
CL(68:5)	0.040	*0.007*	0.171	*0.027*	3.2E-04	*
CL(70:7)	3.179	*0.445*	9.236	*0.353*	4.2E-08	*
CL(70:6)	0.226	*0.021*	1.232	*0.287*	3.6E-03	*
CL(70:5)	0.113	*0.019*	0.283	*0.049*	6.3E-03	*
CL(70:4)	0.072	*0.008*	0.153	*0.025*	8.2E-03	*
CL(72:9)	0.584	*0.024*	0.634	*0.044*	3.4E-01	
CL(72:8)	75.177	*1.086*	55.593	*1.928*	4.1E-07	*
CL(72:7)	15.359	*0.392*	19.986	*0.895*	3.2E-04	*
CL(72:5)	0.009	*0.002*	0.027	*0.004*	2.0E-03	*
CL(74:11)	0.031	*0.007*	0.184	*0.035*	7.5E-04	*
CL(74:10)	1.698	*0.132*	2.981	*0.367*	5.4E-03	*
CL(74:9)	1.311	*0.129*	4.833	*0.276*	1.5E-08	*
CL(74:8)	0.323	*0.023*	0.452	*0.115*	2.9E-01	
CL(76:12)	0.704	*0.088*	1.217	*0.207*	3.9E-02	*
CL(76:11)	0.720	*0.087*	1.651	*0.274*	6.0E-03	*
CL(76:10)	0.221	*0.023*	0.504	*0.062*	7.3E-04	*
CL(78:13)	0.010	*0.003*	0.124	*0.031*	2.6E-03	*
CL(78:12)	0.007	*0.002*	0.088	*0.019*	8.9E-04	*
CL	100.000	*0.000*	100.000	*0.000*		

MLCL(52:5)	1.333	*0.295*	7.694	*0.464*	1.5E-08	*
MLCL(52:4)	0.067	*0.029*	0.486	*0.147*	1.5E-02	*
MLCL(54:6)	94.082	*0.804*	73.409	*2.600*	2.5E-06	*
MLCL(54:5)	2.883	*0.225*	10.543	*1.301*	4.6E-05	*
MLCL(54:4)	0.092	*0.061*	0.302	*0.106*	1.1E-01	
MLCL(56:8)	0.779	*0.208*	2.298	*0.493*	1.3E-02	*
MLCL(56:7)	0.371	*0.097*	3.545	*0.350*	4.8E-07	*
MLCL(58:10)	0.225	*0.090*	0.804	*0.271*	6.2E-02	
MLCL(58:9)	0.167	*0.058*	0.919	*0.318*	3.5E-02	*
MLCL	100.000	*0.000*	100.000	*0.000*		
SM(34:2:2)	0.215	*0.054*	0.171	*0.030*	4.8E-01	
SM(34:1:2)	16.576	*0.472*	19.498	*0.599*	1.8E-03	*
SM(36:2:2)	1.641	*0.139*	1.497	*0.169*	5.2E-01	
SM(36:1:2)	15.764	*0.647*	17.895	*0.505*	2.1E-02	*
SM(38:2:2)	3.001	*0.193*	2.288	*0.176*	1.6E-02	*
SM(38:1:2)	11.695	*0.430*	10.725	*0.254*	7.2E-02	
SM(39:2:2)	0.136	*0.025*	0.039	*0.007*	2.4E-03	*
SM(39:1:2)	1.830	*0.093*	1.030	*0.089*	2.3E-05	*
SM(40:2:2)	4.685	*0.177*	3.586	*0.128*	1.8E-04	*
SM(40:1:2)	14.191	*0.429*	10.393	*0.392*	1.3E-05	*
SM(41:2:2)	2.034	*0.197*	1.649	*0.140*	1.3E-01	
SM(41:1:2)	5.672	*0.313*	4.929	*0.287*	1.0E-01	
SM(42:2:2)	9.595	*0.326*	14.317	*0.672*	1.9E-05	*
SM(41:2:3)	0.635	*0.193*	0.570	*0.162*	8.0E-01	
SM(42:1:2)	12.330	*0.423*	11.413	*0.471*	1.7E-01	
SM	100.000	*0.000*	100.000	*0.000*		
Cer(34:1:2)	4.307	*0.602*	4.121	*0.374*	8.0E-01	
Cer(36:1:2)	4.304	*0.484*	4.461	*0.697*	8.6E-01	
Cer(38:1:2)	2.941	*0.428*	3.303	*0.454*	5.7E-01	
Cer(40:2:2)	2.294	*0.321*	1.654	*0.481*	2.9E-01	
Cer(40:1:2)	14.259	*0.593*	11.783	*0.990*	5.0E-02	*
Cer(41:2:2)	1.543	*0.305*	1.759	*0.331*	6.4E-01	
Cer(41:1:2)	11.124	*0.832*	9.500	*0.971*	2.2E-01	
Cer(42:3:2)	1.137	*0.227*	3.518	*0.278*	1.1E-05	*
Cer(42:2:2)	21.803	*1.523*	28.367	*1.574*	9.6E-03	*
Cer(42:1:2)	36.287	*1.101*	31.534	*2.228*	7.6E-02	
Cer	100.000	*0.000*	100.000	*0.000*		
GM3(34:1:2)	4.409	*0.472*	5.439	*0.796*	2.8E-01	
GM3(36:1:2)	13.168	*1.989*	18.998	*0.607*	1.4E-02	*
GM3(38:1:2)	31.316	*1.250*	27.261	*1.110*	2.9E-02	*
GM3(40:1:2)	23.514	*1.241*	17.519	*0.789*	1.1E-03	*
GM3(42:2:2)	15.315	*2.445*	17.552	*1.777*	4.7E-01	
GM3(42:1:2)	12.278	*1.970*	13.232	*0.728*	6.6E-01	
GM3	100.000	*0.000*	100.000	*0.000*		
DG(34:2)	6.552	*1.860*	6.137	*1.222*	8.5E-01	
DG(34:1)	23.094	*0.818*	28.814	*1.121*	1.0E-03	*
DG(36:4)	7.098	*1.535*	3.312	*0.387*	3.1E-02	*
DG(36:3)	6.401	*0.750*	4.177	*1.338*	1.7E-01	
DG(36:2)	18.032	*2.765*	18.549	*1.495*	8.7E-01	

DG(38:5)	2.082	*0.520*	2.561	*0.464*	5.0E-01	
DG(38:4)	36.741	*1.672*	36.449	*3.341*	9.4E-01	
DG	100.000	*0.000*	100.000	*0.000*		
TG(50:5)	0.047	*0.022*	0.005	*0.004*	8.3E-02	
TG(50:4)	1.376	*0.140*	1.308	*0.462*	8.9E-01	
TG(50:3)	3.171	*0.942*	1.426	*0.707*	1.6E-01	
TG(50:2)	9.790	*0.889*	9.765	*1.019*	9.9E-01	
TG(50:1)	6.076	*0.465*	9.513	*0.432*	9.1E-05	*
TG(50:0)	0.724	*0.155*	1.091	*0.231*	2.1E-01	
TG(52:5)	1.459	*0.264*	0.431	*0.110*	2.9E-03	*
TG(52:4)	8.576	*0.821*	3.838	*0.290*	8.7E-05	*
TG(52:3)	17.029	*0.893*	14.696	*1.040*	1.1E-01	
TG(52:2)	10.565	*1.080*	17.699	*1.133*	4.5E-04	*
TG(52:1)	1.792	*0.301*	2.091	*0.219*	4.3E-01	
TG(52:0)	0.220	*0.047*	0.401	*0.104*	1.3E-01	
TG(54:7)	0.257	*0.070*	0.032	*0.008*	6.2E-03	*
TG(54:6)	3.219	*0.384*	1.020	*0.193*	1.6E-04	*
TG(54:5)	10.546	*1.572*	10.520	*3.840*	1.0E+00	
TG(54:4)	8.455	*0.379*	7.366	*0.446*	8.4E-02	
TG(54:3)	5.694	*0.472*	9.290	*0.602*	3.4E-04	*
TG(54:2)	1.702	*0.332*	1.945	*0.150*	5.2E-01	
TG(56:8)	0.795	*0.150*	0.026	*0.012*	1.5E-04	*
TG(56:7)	1.642	*0.253*	0.959	*0.164*	4.0E-02	*
TG(56:6)	2.270	*0.365*	2.067	*0.294*	6.7E-01	
TG(56:5)	1.879	*0.397*	2.839	*0.590*	2.0E-01	
TG(56:4)	0.402	*0.084*	0.323	*0.077*	5.0E-01	
TG(56:3)	0.398	*0.214*	0.274	*0.046*	5.8E-01	
TG(58:9)	0.348	*0.099*	0.055	*0.023*	1.2E-02	*
TG(58:8)	0.680	*0.145*	0.173	*0.040*	4.5E-03	*
TG(58:7)	0.473	*0.122*	0.429	*0.096*	7.8E-01	
TG(58:6)	0.341	*0.097*	0.350	*0.087*	9.5E-01	
TG(58:5)	0.048	*0.033*	0.063	*0.019*	7.0E-01	
TG(58:4)	0.023	*0.014*	0.004	*0.002*	2.0E-01	
TG	100.000	*0.000*	100.000	*0.000*		

Lipid/protein values (nmol/mg)

Lipid/prot values

Data are expressed as lipid/prot (nmol/mg) and given as mean ± SEM, n = 8

****p<0.05 corresponding to false discovery rate <0.05; FC, fold change***

	Control		*Fructose*			
Species name	avg	*SEM*	avg	*SEM*	*p*	
PC(32:1)	0.438	*0.047*	0.573	*0.053*	7.6E-02	
PC(32:0)	3.137	*0.210*	2.579	*0.151*	4.9E-02	*
PC(34:3)	0.209	*0.022*	0.176	*0.026*	3.5E-01	
PC(34:2)	8.960	*0.679*	5.622	*0.749*	5.3E-03	*
PC(34:1)	11.820	*0.817*	13.588	*0.885*	1.6E-01	
PC(34:0)	0.332	*0.025*	0.231	*0.022*	8.8E-03	*
PC(36:5)	0.544	*0.056*	0.553	*0.042*	8.9E-01	
PC(36:4)	29.240	*2.209*	24.736	*1.717*	1.3E-01	
PC(36:3)	3.672	*0.290*	3.894	*0.626*	7.5E-01	
PC(36:2)	11.191	*0.802*	8.764	*1.301*	1.3E-01	
PC(36:1)	4.121	*0.384*	5.292	*0.427*	6.1E-02	
PC(38:6)	5.956	*0.626*	3.837	*0.311*	9.0E-03	*
PC(38:5)	6.617	*0.605*	7.512	*0.652*	3.3E-01	
PC(38:4)	50.217	*3.821*	42.010	*3.426*	1.3E-01	
PC(40:8)	0.423	*0.051*	0.262	*0.022*	1.1E-02	*
PC(40:7)	0.523	*0.046*	0.485	*0.040*	5.3E-01	
PC(40:6)	1.881	*0.198*	1.560	*0.178*	2.5E-01	
PC(40:5)	1.599	*0.166*	1.629	*0.210*	9.1E-01	
PC(40:4)	0.631	*0.059*	0.515	*0.053*	1.6E-01	
**PC**	**141.512**	***9.217***	**123.818**	***8.694***	1.8E-01	
PC-P(34:1)	0.022	*0.008*	0.020	*0.005*	8.1E-01	
PC-P(34:0)	0.033	*0.005*	0.044	*0.005*	1.3E-01	
PC-P(36:4)	1.368	*0.461*	1.066	*0.197*	5.6E-01	
PC-P(38:5)	0.255	*0.084*	0.223	*0.044*	7.5E-01	
PC-P(38:4)	0.117	*0.032*	0.084	*0.015*	3.6E-01	
PC-P	**1.795**	***0.584***	**1.438**	***0.259***	5.8E-01	
LPC(16:0)	0.192	*0.015*	0.179	*0.011*	5.0E-01	
LPC(18:2)	0.003	*0.001*	0.002	*0.001*	2.9E-01	
LPC(18:1)	0.018	*0.003*	0.033	*0.005*	2.2E-02	*
LPC(18:0)	0.319	*0.028*	0.265	*0.008*	8.8E-02	
LPC(20:4)	0.042	*0.010*	0.031	*0.007*	3.8E-01	
**LPC**	**0.574**	***0.042***	**0.510**	***0.027***	2.2E-01	
LPE(16:0)	0.013	*0.002*	0.020	*0.004*	1.4E-01	
LPE(18:1)	0.105	*0.006*	0.098	*0.006*	4.4E-01	
LPE(18:0)	0.093	*0.011*	0.092	*0.007*	9.5E-01	
**LPE**	**0.211**	***0.017***	**0.210**	***0.012***	9.6E-01	
PE(34:2)	1.972	*0.170*	0.940	*0.093*	1.1E-04	*
PE(34:1)	1.257	*0.103*	1.461	*0.092*	1.6E-01	
PE(36:4)	4.731	*0.338*	4.862	*0.281*	7.7E-01	
PE(36:3)	2.038	*0.185*	1.218	*0.196*	8.9E-03	*
PE(36:2)	5.978	*0.558*	3.078	*0.394*	8.2E-04	*

PE(36:1)	0.685	*0.054*	0.709	*0.061*	7.7E-01	
PE(38:6)	6.164	*0.617*	4.020	*0.487*	1.6E-02	*
PE(38:5)	5.173	*0.456*	7.405	*0.544*	7.2E-03	*
PE(38:4)	27.399	*1.991*	24.857	*1.528*	3.3E-01	
PE(38:3)	0.066	*0.016*	0.232	*0.043*	2.7E-03	*
PE(40:7)	1.635	*0.143*	1.676	*0.129*	8.3E-01	
PE(40:6)	14.102	*1.342*	11.596	*1.206*	1.9E-01	
PE(40:5)	8.069	*1.100*	8.964	*1.166*	5.9E-01	
PE(40:4)	1.430	*0.119*	0.985	*0.076*	7.0E-03	*
**PE**	**80.697**	***5.481***	**72.002**	***4.897***	2.6E-01	
PE-P(34:2)	0.481	*0.022*	0.270	*0.024*	1.5E-05	*
PE-P(34:1)	0.252	*0.019*	0.322	*0.034*	9.4E-02	
PE-P(36:4)	10.427	*0.809*	9.549	*0.777*	4.5E-01	
PE-P(36:2)	0.218	*0.014*	0.151	*0.015*	5.3E-03	*
PE-P(38:6)	3.774	*0.302*	2.052	*0.161*	1.8E-04	*
PE-P(38:5)	7.493	*0.757*	7.046	*0.764*	6.8E-01	
PE-P(38:4)	6.402	*0.543*	4.168	*0.400*	5.1E-03	*
PE-P(40:7)	1.237	*0.109*	0.908	*0.072*	2.5E-02	*
PE-P(40:6)	1.322	*0.115*	0.833	*0.071*	2.8E-03	*
PE-P(40:5)	0.643	*0.050*	0.484	*0.040*	2.7E-02	*
PE-P(40:4)	0.388	*0.028*	0.272	*0.020*	4.5E-03	*
**PE-P**	**32.636**	***2.503***	**26.057**	***2.275***	7.2E-02	
LPI(18:1)	0.055	*0.002*	0.051	*0.004*	3.6E-01	
LPI(18:0)	0.012	*0.001*	0.010	*0.001*	3.3E-01	
LPI(20:4)	0.015	*0.002*	0.011	*0.002*	2.0E-01	
**LPI**	**0.082**	***0.005***	**0.073**	***0.005***	1.8E-01	
PI(34:2)	0.138	*0.010*	0.075	*0.007*	1.3E-04	*
PI(34:1)	0.011	*0.009*	0.086	*0.023*	9.6E-03	*
PI(36:4)	0.388	*0.026*	0.362	*0.024*	4.8E-01	
PI(36:3)	0.256	*0.011*	0.193	*0.028*	5.7E-02	
PI(36:2)	1.483	*0.075*	0.620	*0.064*	5.1E-07	*
PI(36:1)	0.106	*0.009*	0.126	*0.013*	2.2E-01	
PI(38:6)	0.062	*0.005*	0.030	*0.003*	1.2E-04	*
PI(38:5)	0.585	*0.036*	0.747	*0.064*	4.5E-02	*
PI(38:4)	11.499	*0.825*	9.650	*0.645*	9.9E-02	
PI(40:6)	0.119	*0.014*	0.069	*0.006*	4.8E-03	*
PI(40:5)	0.130	*0.017*	0.091	*0.008*	5.8E-02	
PI(40:4)	0.071	*0.008*	0.047	*0.003*	1.8E-02	*
**PI**	**14.848**	***0.974***	**12.097**	***0.791***	4.6E-02	*
LPS(18:1)	0.038	*0.001*	0.036	*0.003*	5.4E-01	
**LPS**	**0.038**	***0.001***	**0.036**	***0.003***	5.4E-01	
PS(34:1)	0.018	*0.003*	0.032	*0.003*	6.0E-03	*
PS(36:2)	0.410	*0.027*	0.272	*0.026*	2.5E-03	*
PS(36:1)	0.559	*0.043*	0.554	*0.050*	9.4E-01	
PS(38:6)	0.019	*0.003*	0.010	*0.002*	2.3E-02	*
PS(38:5)	0.026	*0.004*	0.036	*0.004*	6.8E-02	
PS(38:4)	1.260	*0.103*	0.975	*0.062*	3.3E-02	*
PS(38:3)	0.138	*0.010*	0.167	*0.018*	1.7E-01	
PS(38:2)	0.010	*0.001*	0.015	*0.001*	3.8E-02	*

PS(40:7)	0.015	*0.003*	0.027	*0.004*	3.0E-02	*
PS(40:6)	3.368	*0.310*	2.687	*0.177*	7.8E-02	
PS(40:5)	2.270	*0.275*	2.556	*0.228*	4.4E-01	
PS(40:4)	0.985	*0.074*	0.690	*0.042*	3.8E-03	*
PS(40:3)	0.070	*0.021*	0.087	*0.013*	5.0E-01	
**PS**	**9.148**	***0.716***	**8.108**	***0.537***	2.6E-01	
LPG(18:2)	0.002	*0.001*	0.001	*0.000*	1.1E-01	
LPG(18:1)	0.003	*0.000*	0.004	*0.000*	2.0E-02	*
**LPG**	**0.005**	***0.001***	**0.005**	***0.001***	9.2E-01	
PG(32:1)	0.009	*0.002*	0.017	*0.002*	1.1E-02	*
PG(34:2)	0.564	*0.035*	0.298	*0.022*	1.7E-05	*
PG(34:1)	3.376	*0.244*	3.294	*0.222*	8.1E-01	
PG(36:4)	0.131	*0.008*	0.081	*0.006*	1.4E-04	*
PG(36:3)	0.169	*0.009*	0.147	*0.011*	1.3E-01	
PG(36:2)	0.472	*0.041*	0.311	*0.030*	7.3E-03	*
PG(36:1)	0.155	*0.017*	0.132	*0.009*	2.5E-01	
PG(38:6)	0.042	*0.006*	0.013	*0.003*	2.8E-04	*
PG(38:5)	0.033	*0.004*	0.041	*0.004*	1.8E-01	
PG(38:4)	0.076	*0.007*	0.065	*0.005*	1.9E-01	
PG(40:8)	0.025	*0.005*	0.002	*0.001*	4.9E-04	*
PG(40:7)	0.039	*0.006*	0.025	*0.004*	7.2E-02	
PG(40:6)	0.037	*0.005*	0.048	*0.005*	1.4E-01	
**PG**	**5.129**	***0.339***	**4.472**	***0.280***	1.6E-01	
PA(34:2)	0.036	*0.003*	0.030	*0.004*	2.0E-01	
PA(34:1)	0.122	*0.011*	0.131	*0.012*	6.1E-01	
PA(36:4)	0.019	*0.004*	0.012	*0.002*	1.6E-01	
PA(36:3)	0.011	*0.002*	0.013	*0.002*	4.8E-01	
PA(36:2)	0.042	*0.006*	0.042	*0.005*	9.7E-01	
PA(36:1)	0.003	*0.001*	0.003	*0.001*	7.8E-01	
PA(38:5)	0.009	*0.003*	0.009	*0.002*	9.2E-01	
**PA**	**0.241**	***0.025***	**0.239**	***0.023***	9.5E-01	
CL(68:6)	0.080	*0.008*	0.180	*0.015*	3.7E-05	*
CL(68:5)	0.015	*0.002*	0.045	*0.005*	5.7E-05	*
CL(70:7)	1.189	*0.166*	2.590	*0.230*	2.1E-04	*
CL(70:6)	0.084	*0.006*	0.312	*0.062*	2.6E-03	*
CL(70:5)	0.043	*0.007*	0.075	*0.010*	1.8E-02	*
CL(70:4)	0.028	*0.004*	0.040	*0.005*	6.3E-02	
CL(72:9)	0.219	*0.011*	0.173	*0.011*	9.7E-03	*
CL(72:8)	28.462	*1.662*	15.713	*1.417*	4.3E-05	*
CL(72:7)	5.829	*0.409*	5.639	*0.525*	7.8E-01	
CL(72:5)	0.004	*0.001*	0.008	*0.001*	3.5E-02	*
CL(74:11)	0.011	*0.002*	0.048	*0.008*	6.3E-04	*
CL(74:10)	0.637	*0.052*	0.806	*0.093*	1.3E-01	
CL(74:9)	0.489	*0.045*	1.365	*0.148*	5.8E-05	*
CL(74:8)	0.122	*0.010*	0.114	*0.025*	7.8E-01	
CL(76:12)	0.264	*0.034*	0.325	*0.053*	3.4E-01	
CL(76:11)	0.271	*0.035*	0.449	*0.080*	6.1E-02	
CL(76:10)	0.084	*0.011*	0.138	*0.018*	2.4E-02	*
CL(78:13)	0.004	*0.001*	0.033	*0.008*	2.6E-03	*
CL(78:12)	0.003	*0.001*	0.023	*0.005*	1.1E-03	*
**CL**	**37.837**	***2.117***	**28.078**	***2.144***	5.9E-03	*

MLCL(52:5)	0.006	*0.001*	0.037	*0.005*	2.8E-05	*
MLCL(52:4)	0.000	*0.000*	0.002	*0.001*	1.2E-02	*
MLCL(54:6)	0.378	*0.049*	0.342	*0.037*	5.6E-01	
MLCL(54:5)	0.012	*0.002*	0.051	*0.009*	1.2E-03	*
MLCL(54:4)	0.000	*0.000*	0.002	*0.001*	5.7E-02	
MLCL(56:8)	0.004	*0.001*	0.012	*0.004*	5.9E-02	
MLCL(56:7)	0.002	*0.001*	0.017	*0.003*	5.0E-04	*
MLCL(58:10)	0.001	*0.001*	0.005	*0.002*	9.9E-02	
MLCL(58:9)	0.001	*0.000*	0.005	*0.002*	9.1E-02	
**MLCL**	**0.404**	***0.055***	**0.473**	***0.059***	4.1E-01	
SM(34:2:2)	0.014	*0.004*	0.010	*0.002*	3.3E-01	
SM(34:1:2)	1.052	*0.095*	1.093	*0.080*	7.5E-01	
SM(36:2:2)	0.104	*0.011*	0.089	*0.012*	3.9E-01	
SM(36:1:2)	0.991	*0.082*	1.016	*0.091*	8.4E-01	
SM(38:2:2)	0.187	*0.016*	0.132	*0.014*	2.1E-02	*
SM(38:1:2)	0.732	*0.054*	0.605	*0.046*	9.2E-02	
SM(39:2:2)	0.009	*0.002*	0.002	*0.000*	3.7E-03	*
SM(39:1:2)	0.115	*0.010*	0.058	*0.006*	2.5E-04	*
SM(40:2:2)	0.294	*0.023*	0.204	*0.017*	7.1E-03	*
SM(40:1:2)	0.890	*0.066*	0.577	*0.034*	8.8E-04	*
SM(41:2:2)	0.128	*0.017*	0.094	*0.011*	9.8E-02	
SM(41:1:2)	0.356	*0.033*	0.270	*0.012*	2.7E-02	*
SM(42:2:2)	0.606	*0.055*	0.817	*0.083*	5.3E-02	
SM(41:2:3)	0.044	*0.016*	0.034	*0.010*	6.2E-01	
SM(42:1:2)	0.777	*0.068*	0.631	*0.034*	7.7E-02	
**SM**	**6.301**	***0.481***	**5.632**	***0.407***	3.1E-01	
Cer(34:1:2)	0.008	*0.001*	0.009	*0.001*	7.4E-01	
Cer(36:1:2)	0.008	*0.001*	0.010	*0.002*	4.5E-01	
Cer(38:1:2)	0.005	*0.001*	0.007	*0.001*	2.2E-01	
Cer(40:2:2)	0.004	*0.001*	0.004	*0.001*	6.3E-01	
Cer(40:1:2)	0.027	*0.002*	0.026	*0.003*	8.2E-01	
Cer(41:2:2)	0.003	*0.001*	0.004	*0.001*	4.4E-01	
Cer(41:1:2)	0.022	*0.003*	0.020	*0.002*	7.2E-01	
Cer(42:3:2)	0.002	*0.000*	0.008	*0.001*	5.4E-04	*
Cer(42:2:2)	0.041	*0.004*	0.061	*0.005*	7.1E-03	*
Cer(42:1:2)	0.069	*0.005*	0.067	*0.005*	8.0E-01	
**Cer**	**0.190**	***0.014***	**0.216**	***0.017***	2.6E-01	
GM3(34:1:2)	0.033	*0.003*	0.035	*0.004*	6.7E-01	
GM3(36:1:2)	0.102	*0.016*	0.129	*0.009*	1.5E-01	
GM3(38:1:2)	0.242	*0.017*	0.186	*0.014*	2.4E-02	*
GM3(40:1:2)	0.182	*0.015*	0.119	*0.009*	2.8E-03	*
GM3(42:2:2)	0.115	*0.015*	0.122	*0.018*	7.5E-01	
GM3(42:1:2)	0.099	*0.021*	0.089	*0.005*	6.4E-01	
**GM3**	**0.772**	***0.048***	**0.681**	***0.042***	1.7E-01	
DG(34:2)	0.090	*0.024*	0.124	*0.045*	5.2E-01	
DG(34:1)	0.368	*0.049*	0.453	*0.106*	4.7E-01	
DG(36:4)	0.103	*0.018*	0.050	*0.011*	2.8E-02	*
DG(36:3)	0.098	*0.011*	0.097	*0.044*	9.9E-01	
DG(36:2)	0.315	*0.102*	0.332	*0.103*	9.1E-01	

DG(38:5)	0.033	*0.008*	0.037	*0.009*	7.5E-01	
DG(38:4)	0.575	*0.058*	0.502	*0.063*	4.0E-01	
**DG**	**1.581**	***0.186***	**1.596**	***0.365***	9.7E-01	
TG(50:5)	0.008	*0.004*	0.001	*0.001*	1.3E-01	
TG(50:4)	0.182	*0.041*	0.090	*0.020*	6.3E-02	
TG(50:3)	0.534	*0.166*	0.287	*0.148*	2.8E-01	
TG(50:2)	1.393	*0.324*	1.116	*0.382*	5.9E-01	
TG(50:1)	0.799	*0.169*	0.990	*0.319*	6.1E-01	
TG(50:0)	0.071	*0.005*	0.065	*0.007*	4.6E-01	
TG(52:5)	0.213	*0.064*	0.056	*0.021*	3.5E-02	*
TG(52:4)	1.208	*0.330*	0.406	*0.128*	4.0E-02	*
TG(52:3)	2.394	*0.550*	1.635	*0.553*	3.5E-01	
TG(52:2)	1.460	*0.363*	1.993	*0.705*	5.1E-01	
TG(52:1)	0.193	*0.017*	0.169	*0.038*	5.9E-01	
TG(52:0)	0.021	*0.001*	0.022	*0.003*	9.3E-01	
TG(54:7)	0.039	*0.015*	0.003	*0.001*	3.2E-02	*
TG(54:6)	0.411	*0.115*	0.098	*0.027*	1.9E-02	*
TG(54:5)	1.229	*0.265*	0.625	*0.117*	5.6E-02	
TG(54:4)	1.151	*0.260*	0.811	*0.285*	3.9E-01	
TG(54:3)	0.774	*0.184*	1.043	*0.375*	5.3E-01	
TG(54:2)	0.183	*0.019*	0.180	*0.050*	9.6E-01	
TG(56:8)	0.084	*0.013*	0.004	*0.002*	1.8E-05	*
TG(56:7)	0.178	*0.019*	0.083	*0.020*	3.9E-03	*
TG(56:6)	0.243	*0.021*	0.171	*0.041*	1.4E-01	
TG(56:5)	0.186	*0.011*	0.198	*0.039*	7.8E-01	
TG(56:4)	0.044	*0.008*	0.038	*0.014*	7.1E-01	
TG(56:3)	0.030	*0.009*	0.033	*0.014*	8.6E-01	
TG(58:9)	0.033	*0.006*	0.004	*0.001*	1.8E-04	*
TG(58:8)	0.070	*0.009*	0.019	*0.006*	5.5E-04	*
TG(58:7)	0.051	*0.010*	0.041	*0.012*	5.4E-01	
TG(58:6)	0.035	*0.008*	0.034	*0.011*	9.2E-01	
TG(58:5)	0.005	*0.003*	0.006	*0.002*	7.9E-01	
TG(58:4)	0.002	*0.001*	0.000	*0.000*	1.4E-01	
**TG**	**13.226**	***2.609***	**10.219**	***3.276***	4.8E-01	
Class						
PC	141.512	*9.217*	123.818	*8.694*	1.8E-01	
PC-P	1.795	*0.584*	1.438	*0.259*	5.8E-01	
LPC	0.574	*0.042*	0.510	*0.027*	2.2E-01	
LPE	0.211	*0.017*	0.210	*0.012*	9.6E-01	
PE	80.697	*5.481*	72.002	*4.897*	2.6E-01	
PE-P	32.636	*2.503*	26.057	*2.275*	7.2E-02	
LPI	0.082	*0.005*	0.073	*0.005*	1.8E-01	
PI	14.848	*0.974*	12.097	*0.791*	4.6E-02	*
LPS	0.038	*0.001*	0.036	*0.003*	5.4E-01	
PS	9.148	*0.716*	8.108	*0.537*	2.6E-01	
LPG	0.005	*0.001*	0.005	*0.001*	9.2E-01	
PG	5.129	*0.339*	4.472	*0.280*	1.6E-01	
PA	0.241	*0.025*	0.239	*0.023*	9.5E-01	
CL	37.837	*2.117*	28.078	*2.144*	5.9E-03	*
MLCL	0.404	*0.055*	0.473	*0.059*	4.1E-01	
SM	6.301	*0.481*	5.632	*0.407*	3.1E-01	
Cer	0.190	*0.014*	0.216	*0.017*	2.6E-01	
GM3	0.772	*0.048*	0.681	*0.042*	1.7E-01	
DG	1.581	*0.186*	1.596	*0.365*	9.7E-01	
TG	13.226	*2.609*	10.219	*3.276*	4.8E-01	

sumML/prot (nmol/mg)mg	332.421	*21.731*	284.143	*19.348*	1.2E-01	

Double bonds (db) and double bond index (DBI)

db data are expressed as mol% of membrane lipids and given as mean ± SEM, n = 8, *p<0.05

The double bond index (DBI) was calculated for the major membrane phospholipids and TG as Σ(mol % of unsaturated fatty acids in the given lipid class x number of double bonds of each unsaturated fatty acid)/100.

	Control		*Fructose*			
Species name	avg	*SEM*	avg	*SEM*	*p*	
PC [db 0]	2.459	*0.083*	2.308	*0.111*	2.9E-01	
PC [db 1]	11.565	*0.309*	15.788	*0.460*	2.4E-06	*
PC [db 2]	14.455	*1.086*	11.453	*1.377*	1.1E-01	
PC [db 3]	2.761	*0.169*	3.238	*0.465*	3.5E-01	
PC [db 4]	56.444	*1.094*	54.477	*1.854*	3.8E-01	
PC [db 5]	6.155	*0.220*	7.785	*0.245*	2.2E-04	*
PC [db 6]	5.496	*0.321*	4.350	*0.191*	8.4E-03	*
PC [db 7]	0.367	*0.011*	0.391	*0.014*	2.0E-01	
PC [db 8]	0.298	*0.029*	0.210	*0.009*	1.2E-02	*
PE [db 1]	2.423	*0.143*	3.036	*0.143*	9.0E-03	*
PE [db 2]	9.908	*0.787*	5.614	*0.627*	7.8E-04	*
PE [db 3]	2.639	*0.230*	2.024	*0.317*	1.4E-01	
PE [db 4]	41.636	*1.376*	42.888	*0.981*	4.7E-01	
PE [db 5]	16.408	*1.274*	22.519	*0.858*	1.4E-03	*
PE [db 6]	24.968	*1.070*	21.593	*1.344*	7.0E-02	
PE [db 7]	2.019	*0.077*	2.327	*0.063*	7.8E-03	*
PE-Pl [db 1]	0.779	*0.039*	1.232	*0.073*	8.4E-05	*
PE-Pl [db 2]	2.195	*0.143*	1.616	*0.065*	2.4E-03	*
PE-Pl [db 4]	52.737	*1.152*	53.744	*0.495*	4.4E-01	
PE-Pl [db 5]	24.780	*1.092*	28.619	*0.710*	1.1E-02	*
PE-Pl [db 6]	15.702	*0.678*	11.244	*0.591*	2.1E-04	*
PE-Pl [db 7]	3.807	*0.190*	3.545	*0.197*	3.6E-01	
PI [db 1]	0.807	*0.101*	1.756	*0.255*	3.9E-03	*
PI [db 2]	11.049	*0.522*	5.739	*0.410*	1.4E-06	*
PI [db 3]	1.760	*0.116*	1.585	*0.209*	4.8E-01	
PI [db 4]	80.353	*0.560*	83.176	*1.112*	4.0E-02	*
PI [db 5]	4.824	*0.144*	6.924	*0.318*	3.2E-05	*
PI [db 6]	1.206	*0.052*	0.821	*0.042*	4.7E-05	*
PS [db 1]	6.314	*0.159*	7.190	*0.350*	3.9E-02	*
PS [db 2]	4.711	*0.357*	3.550	*0.257*	2.0E-02	*
PS [db 3]	2.324	*0.307*	3.120	*0.180*	4.2E-02	*
PS [db 4]	24.573	*0.454*	20.610	*0.340*	6.4E-06	*
PS [db 5]	24.966	*1.929*	31.700	*1.337*	1.2E-02	*
PS [db 6]	36.955	*1.936*	33.503	*1.381*	1.7E-01	
PS [db 7]	0.158	*0.024*	0.328	*0.039*	2.3E-03	*
PG [db 1]	68.881	*1.112*	76.949	*0.919*	6.6E-05	*
PG [db 2]	20.330	*0.832*	13.640	*0.974*	1.3E-04	*
PG [db 3]	3.346	*0.153*	3.300	*0.220*	8.7E-01	

PG [db 4]	4.045	*0.132*	3.233	*0.097*	2.2E-04	*
PG [db 5]	0.638	*0.048*	0.917	*0.072*	6.2E-03	*
PG [db 6]	1.529	*0.110*	1.357	*0.109*	2.9E-01	
PG [db 7]	0.739	*0.063*	0.558	*0.073*	8.2E-02	
PG [db 8]	0.493	*0.108*	0.046	*0.013*	1.1E-03	*
PA [db 1]	52.428	*2.353*	56.112	*1.957*	2.5E-01	
PA [db 2]	32.448	*1.419*	29.434	*1.471*	1.6E-01	
PA [db 3]	4.460	*0.484*	5.318	*0.502*	2.4E-01	
PA [db 4]	7.403	*1.053*	5.130	*0.716*	9.6E-02	
PA [db 5]	3.260	*1.026*	4.007	*1.130*	6.3E-01	
CL [db 4]	0.072	*0.008*	0.153	*0.025*	8.2E-03	*
CL [db 5]	0.163	*0.020*	0.481	*0.076*	1.2E-03	*
CL [db 6]	0.441	*0.042*	1.883	*0.297*	2.8E-04	*
CL [db 7]	18.538	*0.698*	29.222	*0.655*	2.3E-08	*
CL [db 8]	75.500	*1.086*	56.044	*1.834*	2.8E-07	*
CL [db 9]	1.895	*0.135*	5.467	*0.281*	1.7E-08	*
CL [db 10]	1.919	*0.143*	3.486	*0.423*	3.5E-03	*
CL [db 11]	0.751	*0.091*	1.835	*0.305*	4.3E-03	*
CL [db 12]	0.711	*0.089*	1.304	*0.224*	2.8E-02	*
CL [db 13]	0.010	*0.003*	0.124	*0.031*	2.6E-03	*
TG [db 0]	0.945	*0.200*	1.492	*0.332*	1.8E-01	
TG [db 1]	7.868	*0.552*	11.604	*0.509*	2.0E-04	*
TG [db 2]	22.057	*1.743*	29.409	*1.999*	1.5E-02	*
TG [db 3]	26.292	*1.738*	25.686	*1.996*	8.2E-01	
TG [db 4]	18.833	*1.107*	12.840	*0.228*	1.1E-04	*
TG [db 5]	13.979	*1.847*	13.858	*4.315*	9.8E-01	
TG [db 6]	5.830	*0.673*	3.437	*0.526*	1.4E-02	*
TG [db 7]	2.373	*0.338*	1.420	*0.261*	4.3E-02	*
TG [db 8]	1.475	*0.289*	0.199	*0.048*	6.5E-04	*
TG [db 9]	0.348	*0.099*	0.055	*0.023*	1.2E-02	*
Class DBI						
PC [DBI]	3.432	*0.026*	3.358	*0.046*	1.8E-01	
PE [DBI]	4.427	*0.043*	4.503	*0.051*	2.7E-01	
PE-Pl [DBI]	4.609	*0.020*	4.548	*0.016*	3.2E-02	*
PI [DBI]	3.810	*0.015*	3.902	*0.015*	6.7E-04	*
PS [DBI]	4.687	*0.028*	4.679	*0.032*	8.6E-01	
PG [DBI]	1.572	*0.023*	1.441	*0.008*	7.7E-05	*
PA [DBI]	1.766	*0.067*	1.715	*0.056*	5.6E-01	
CL [DBI]	7.907	*0.008*	7.887	*0.017*	3.2E-01	
TG [DBI]	3.426	*0.103*	3.008	*0.093*	9.2E-03	*
sum DBI						
sum DBI with CL	4.217	*0.019*	4.124	*0.036*	3.7E-02	*
sum DBI wo CL	3.313	*0.028*	3.349	*0.042*	4.9E-01	
db sum for PLs (wo CL)						
db1	6.897	*0.168*	9.299	*0.224*	6.0E-07	*
db2	9.728	*0.683*	7.145	*0.791*	2.7E-02	*
db3	7.201	*0.224*	7.049	*0.414*	7.5E-01	

db4	40.930	*0.555*	41.398	*0.824*	6.5E-01	
db5	9.029	*0.439*	11.366	*0.417*	1.7E-03	*
db6	9.996	*0.398*	8.914	*0.551*	1.3E-01	

Protein quantification

Protein names	Gene names	Entry name	*mean control (intensity)*	*CV control (%)*	*mean fructose-fed (intensity)*	*CV fructose-fed (%)*	P-value	fold change
Alpha-1-inhibitor III	Mug1 LOC297568	D4A6E3_RAT	*76039*	*26*	*475882*	*89*	0.018	8.82
Sarcolemmal membrane-associated protein (Slmap protein)	Slmap	B5DF63_RAT	*19779*	*6*	*278041*	*149*	0.029	6.55
LIM domain-binding 3	Ldb3	A0A0G2JXR0_RAT	*194927*	*7*	*1134344*	*50*	≤0.001	5.90
Alpha-crystallin B chain (Alpha(B)-crystallin)	Cryab	CRYAB_RAT	*221980*	*11*	*1415791*	*146*	0.047	5.56
3-hydroxyisobutyryl-CoA hydrolase, mitochondrial (EC 3.1.2.4) (3-hydroxyisobutyryl-coenzyme A hydrolase) (HIB-CoA hydrolase) (HIBYL-CoA-H)	Hibch	HIBCH_RAT	*127444*	*110*	*220589*	*73*	0.042	5.44
Prolargin (Proline-arginine-rich end leucine-rich repeat protein)	Prelp	PRELP_RAT	*95044*	*15*	*480320*	*96*	0.001	5.23
Calsequestrin	Casq2 rCG_51991	F1M944_RAT	*652101*	*8*	*3989458*	*98*	0.001	4.95
Myosin light chain 3 (Myosin alkali light chain 1, ventricular) (MLClV) (Myosin light chain 1, slow-twitch muscle B/ventricular isoform) (MLC1SB) (Ventricular myosin light chain 1) (rVMLC1)	Myl3 Mlc1v	MYL3_RAT	*420037*	*12*	*2432804*	*117*	0.002	4.90
WD repeat-containing protein 1	Wdr1	WDR1_RAT	*25059*	*25*	*96155*	*63*	0.039	4.85
Myosin regulatory light chain 2, ventricular/cardiac muscle isoform	Myl2	D3Z9K3_RAT	*418863*	*20*	*1865458*	*69*	0.007	4.58
3-ketoacyl-CoA thiolase, mitochondrial (EC 2.3.1.16) (Acetyl-CoA acyltransferase) (Beta-ketothiolase) (Mitochondrial 3-oxoacyl-CoA thiolase)	Acaa2	THIM_RAT	*642848*	*5*	*5647964*	*178*	0.005	4.56
Core histone macro-H2A.1 (Histone macroH2A1) (mH2A1) (H2A.y) (H2A/y)	H2afy	H2AY_RAT	*115423*	*15*	*478494*	*48*	0.009	4.44
Histone H1.5	Hist1h1b	H15_RAT	*22825740*	*51*	*83180995*	*82*	0.032	4.41
Alpha-aminoadipic semialdehyde dehydrogenase (Alpha-AASA dehydrogenase) (EC 1.2.1.31) (Aldehyde dehydrogenase family 7 member A1) (EC 1.2.1.3) (Antiquitin-1) (Betaine aldehyde dehydrogenase) (EC 1.2.1.8) (Delta1-piperideine-6-carboxylate dehydrogenase) (P6c dehydrogenase)	Aldh7a1 Ald7a1	AL7A1_RAT	*106034*	*7*	*442182*	*5*	≤0.001	4.34
Malic enzyme	Me3	A0A0G2K4C6_RAT	*224396*	*47*	*647274*	*75*	0.022	4.27
Serine protease inhibitor	LOC299282 LOC500712 Serpina3n	A0A0G2KB85_RAT	*278769*	*30*	*818927*	*39*	0.001	3.42
60 kDa heat shock protein, mitochondrial (EC 5.6.1.7) (60 kDa chaperonin) (Chaperonin 60) (CPN60) (HSP-65) (Heat shock protein 60) (HSP-60) (Hsp60) (Mitochondrial matrix protein P1)	Hspd1 Hsp60	CH60_RAT	*85878*	*28*	*244447*	*54*	0.010	3.37
Citrate synthase	Cs rCG_42519	G3V936_RAT	*7464774*	*9*	*25193057*	*54*	≤0.001	3.14

Electron transfer flavoprotein-ubiquinone oxidoreductase, mitochondrial (ETF-QO) (ETF-ubiquinone oxidoreductase) (EC 1.5.5.1) (Electron-transferring-flavoprotein dehydrogenase) (ETF dehydrogenase)	Etfdh	ETFD_RAT	*1389566*	*5*	*6399194*	*108*	0.004	3.13
Platelet glycoprotein 4 (Adipocyte membrane protein) (Fatty acid translocase) (Fatty acid transport protein) (Glycoprotein IIIb) (GPIIIB) (PAS IV) (PAS-4) (Platelet glycoprotein IV) (GPIV) (CD antigen CD36)	Cd36 Fat	CD36_RAT	*172468*	*25*	*462608*	*50*	0.008	3.12
Myosin-6 (Myosin heavy chain 6) (Myosin heavy chain, cardiac muscle alpha isoform) (MyHC-alpha)	Myh6	MYH6_RAT	*61986755*	*20*	*184578704*	*55*	0.003	3.06
Peroxiredoxin-6 (EC 1.11.1.15) (1-Cys peroxiredoxin) (1-Cys PRX) (Acidic calcium-independent phospholipase A2) (aiPLA2) (EC 3.1.1.4) (Antioxidant protein 2) (Non-selenium glutathione peroxidase) (NSGPx) (Thiol-specific antioxidant protein)	Prdx6 Aipla2 Aop2 Tsa	PRDX6_RAT	*536119*	*15*	*1628926*	*58*	0.001	2.87
Myoglobin	Mb	MYG_RAT	*2656386*	*18*	*6507411*	*35*	0.013	2.80
Mannose-6-phosphate isomerase (EC 5.3.1.8) (Phosphohexomutase) (Phosphomannose isomerase) (PMI)	Mpi	MPI_RAT	*549146*	*8*	*2154904*	*76*	0.029	2.74
Platelet-activating factor acetylhydrolase IB subunit beta (EC 3.1.1.47) (PAF acetylhydrolase 30 kDa subunit) (PAF-AH 30 kDa subunit) (PAF-AH subunit beta) (PAFAH subunit beta) (Platelet-activating factor acetylhydrolase alpha 2 subunit) (PAF-AH alpha 2)	Pafah1b2 Pafahb	PA1B2_RAT	*2907760*	*12*	*7667887*	*24*	≤0.001	2.72
ADP/ATP translocase 1 (ADP,ATP carrier protein 1) (Adenine nucleotide translocator 1) (ANT 1) (Solute carrier family 25 member 4)	Slc25a4 Ant1	ADT1_RAT	*5408721*	*5*	*16358449*	*69*	0.005	2.47
Laminin subunit gamma 1	Lamc1	F1MAA7_RAT	*2630586*	*13*	*6156540*	*11*	≤0.001	2.44
Filamin-C (FLN-C) (ABP-280-like protein) (ABP-L) (Actin-binding-like protein) (Filamin-2) (Gamma-filamin)	Flnc Abpl Fln2	FLNC_RAT	*691495*	*43*	*1278490*	*40*	0.034	2.31
Voltage-dependent anion-selective channel protein 3	Vdac3	A0A0G2JSR0_RAT	*706855*	*12*	*1867734*	*84*	0.018	2.17
Radixin	Rdx	A0A0G2K095_RAT	*26410*	*13*	*58358*	*52*	0.014	2.08
Fermitin family member 2	Fermt2	A0A0G2JWC7_RAT	*342282*	*26*	*709270*	*49*	0.014	2.08
NADH dehydrogenase [ubiquinone] 1 alpha subcomplex subunit 2	Ndufa2 Ndufa2_predicted rCG_49583	D3ZS58_RAT	*26710711*	*28*	*46827617*	*35*	0.047	2.06
Cofilin 2 (Cofilin 2, muscle (Predicted), isoform CRA_b)	Cfl2 Cfl2_predicted rCG_62072	M0RC65_RAT	*241814*	*34*	*438288*	*24*	0.006	2.00
Hemopexin	Hpx	HEMO_RAT	*131796*	*22*	*238157*	*14*	0.003	1.95

Tropomyosin alpha-1 chain (Alpha-tropomyosin) (Tropomyosin-1)	Tpm1 Alpha-tm Tpma	TPM1_RAT	*24888098*	*22*	*46057208*	*27*	0.002	1.92
Biglycan (Bone/cartilage proteoglycan I) (PG-S1)	Bgn	PGS1_RAT	*1063640*	*23*	*1894712*	*31*	0.018	1.91
Transgelin-2	Tagln2	TAGL2_RAT	*2533078*	*14*	*4537500*	*20*	≤0.001	1.82
Tropomodulin-1 (Erythrocyte tropomodulin) (E-Tmod)	Tmod1 Tmod	TMOD1_RAT	*1183693*	*11*	*2243877*	*51*	0.002	1.78
Laminin subunit alpha 5 (Laminin, alpha 5, isoform CRA_a)	Lama5 rCG_38528	F1MAN8_RAT	*480972*	*30*	*800729*	*42*	0.048	1.77
Glycerol-3-phosphate dehydrogenase [NAD(+)], cytoplasmic (GPD-C) (GPDH-C) (EC 1.1.1.8)	Gpd1	GPDA_RAT	*20523*	*25*	*34783*	*30*	0.033	1.73
NADH dehydrogenase [ubiquinone] 1 alpha subcomplex subunit 1	Ndufa1 LOC108348144	A0A0U1RRQ1_RAT	*545074*	*19*	*977429*	*46*	0.029	1.71
Aspartate aminotransferase, cytoplasmic (cAspAT) (EC 2.6.1.1) (EC 2.6.1.3) (Cysteine aminotransferase, cytoplasmic) (Cysteine transaminase, cytoplasmic) (cCAT) (Glutamate oxaloacetate transaminase 1) (Transaminase A)	Got1	AATC_RAT	*2822165*	*14*	*5240623*	*64*	0.021	1.70
Troponin I, cardiac muscle (Cardiac troponin I)	Tnni3 Ctni Tni	TNNI3_RAT	*162841838*	*11*	*271569764*	*31*	0.007	1.61
NADH-ubiquinone oxidoreductase chain 4 (EC 7.1.1.2) (NADH dehydrogenase subunit 4)	Mtnd4 mt-Nd4 Nd4	NU4M_RAT	*976935*	*16*	*1580926*	*34*	0.007	1.59
MICOS complex subunit Mic60 (Mitochondrial inner membrane protein) (Mitofilin) (Fragment)	Immt Mic60	MIC60_RAT	*12232588*	*20*	*18907890*	*18*	0.003	1.58
Cytochrome c, testis-specific	Cyct	CYC2_RAT	*71695305*	*22*	*108712209*	*24*	0.011	1.56
Phosphoglycerate kinase 1 (EC 2.7.2.3)	Pgk1 Pgk-1	PGK1_RAT	*20860377*	*7*	*34148076*	*36*	0.011	1.54
Hypoxanthine-guanine phosphoribosyltransferase (HGPRT) (HGPRTase) (EC 2.4.2.8)	Hprt1 Hprt	HPRT_RAT	*2264099*	*15*	*3742948*	*56*	0.048	1.53
Cathepsin D (EC 3.4.23.5) [Cleaved into: Cathepsin D 12 kDa light chain; Cathepsin D 9 kDa light chain; Cathepsin D 34 kDa heavy chain; Cathepsin D 30 kDa heavy chain]	Ctsd	CATD_RAT	*10125331*	*13*	*15354134*	*23*	0.002	1.52
Enoyl CoA hydratase domain-containing 2 (Enoyl Coenzyme A hydratase domain containing 2 (Predicted), isoform CRA_a)	Echdc2 Echdc2_predicted rCG_50417	D3ZIL6_RAT	*3807294*	*10*	*6091062*	*44*	0.018	1.50
Fructose-bisphosphate aldolase A (EC 4.1.2.13) (Muscle-type aldolase)	Aldoa	ALDOA_RAT	*45535968*	*17*	*69169730*	*32*	0.038	1.48
Heterogeneous nuclear ribonucleoprotein A3 (hnRNP A3)	Hnrnpa3 Hnrpa3	ROA3_RAT	*2155257*	*19*	*3070443*	*11*	0.005	1.46
Electron transfer flavoprotein subunit beta (Beta-ETF)	Etfb	ETFB_RAT	*8263784*	*15*	*12139394*	*30*	0.029	1.46
Growth hormone-inducible transmembrane protein (Mitochondrial morphology and cristae structure 1) (MICS1)	Ghitm Mics1	GHITM_RAT	*3579322*	*17*	*5154891*	*27*	0.028	1.45

Prelamin-A/C [Cleaved into: Lamin-A/C]	Lmna Lmn1	LMNA_RAT	*7302903*	*8*	*10221275*	*11*	≤0.001	1.40
Carnitine O-acetyltransferase (Carnitine acetylase) (EC 2.3.1.7) (Carnitine acetyltransferase) (CAT) (CrAT)	Crat	CACP_RAT	*11413004*	*13*	*15796019*	*23*	0.009	1.37
Nicotinamide nucleotide transhydrogenase	Nnt	Q5BJZ3_RAT	*23966346*	*10*	*33184444*	*23*	0.007	1.36
Mitochondrial carnitine/acylcarnitine carrier protein (Carnitine/acylcarnitine translocase) (CAC) (Solute carrier family 25 member 20)	Slc25a20 Cact	MCAT_RAT	*27697292*	*22*	*36433037*	*15*	0.034	1.36
Histone H1.1 (Histone H1a)	Hist1h1a	H11_RAT	*13803990*	*14*	*18807812*	*26*	0.024	1.35
Acyl-CoA synthetase family member 2, mitochondrial (EC 6.2.1.-)	Acsf2	ACSF2_RAT	*5847132*	*12*	*7921102*	*21*	0.011	1.34
Desmoplakin	Dsp	F1LMV6_RAT	*12733679*	*8*	*17267733*	*20*	0.002	1.34
Glutathione S-transferase kappa 1 (EC 2.5.1.18) (GST 13-13) (GST class-kappa) (GSTK1-1) (rGSTK1) (Glutathione S-transferase subunit 13)	Gstk1	GSTK1_RAT	*3288213*	*13*	*4357727*	*16*	0.007	1.33
Bcl2-associated athanogene 3 (RCG40209, isoform CRA_b)	Bag3 rCG_40209	Q5U2U8_RAT	*2532563*	*14*	*3355204*	*18*	0.013	1.33
Hexokinase-1 (EC 2.7.1.1) (Brain form hexokinase) (Hexokinase type I) (HK I)	Hk1	HXK1_RAT	*8794800*	*15*	*11579240*	*17*	0.013	1.32
Myosin-6 (Myosin heavy chain 6) (Myosin heavy chain, cardiac muscle alpha isoform) (MyHC-alpha)	Myh6	MYH6_RAT	*103912915*	*22*	*132925633*	*11*	0.037	1.32
Mitochondrial ribosomal protein S36 (RCG44686, isoform CRA_a)	Mrps36 rCG_44686	M0R776_RAT	*28116950*	*15*	*36414128*	*9*	0.007	1.31
Heterogeneous nuclear ribonucleoprotein M (hnRNP M) (M4 protein)	Hnrnpm Hnrpm	HNRPM_RAT	*5120910*	*10*	*6792210*	*25*	0.018	1.31
Acyl carrier protein	Ndufab1 Ndufab1_predicted rCG_40178	D3ZF13_RAT	*22329527*	*16*	*29071100*	*21*	0.032	1.30
Caveolae-associated protein 1 (Polymerase I and transcript release factor (Predicted))	Cavin1 Ptrf Ptrf_predicted rCG_32735	G3V8L9_RAT	*9389237*	*11*	*12215483*	*18*	0.009	1.29
NADH dehydrogenase [ubiquinone] 1 alpha subcomplex subunit 9, mitochondrial (Complex I-39kD) (CI-39kD) (NADH-ubiquinone oxidoreductase 39 kDa subunit) (Sperm flagella protein 3)	Ndufa9	NDUA9_RAT	*30526703*	*14*	*39103911*	*11*	0.007	1.29
Caveolae-associated protein 2 (Cavin-2) (Phosphatidylserine-binding protein) (Serum deprivation-response protein)	Cavin2 Sdpr	CAVN2_RAT	*5850134*	*14*	*7496480*	*17*	0.024	1.29
Trifunctional enzyme subunit beta, mitochondrial (TP-beta) [Includes: 3-ketoacyl-CoA thiolase (EC 2.3.1.16) (Acetyl-CoA acyltransferase) (Beta-ketothiolase)]	Hadhb	ECHB_RAT	*244590289*	*10*	*315231267*	*16*	0.004	1.29
Adenylate kinase 2, mitochondrial (AK 2) (EC 2.7.4.3) (ATP-AMP transphosphorylase 2) (ATP:AMP phosphotransferase)	Ak2 AK2 rCG_31186	A0A0G2JSG6_RAT	*10137754*	*15*	*12939680*	*15*	0.019	1.28

(Adenylate monophosphate kinase)								
Acyl-coenzyme A thioesterase 2, mitochondrial (Acyl-CoA thioesterase 2) (EC 3.1.2.2) (ARTISt/p43) (Acyl coenzyme A thioester hydrolase) (MTE-I) (Very-long-chain acyl-CoA thioesterase)	Acot2 Mte1	ACOT2_RAT	*136213380*	*13*	*176769180*	*23*	0.030	1.28
60S ribosomal protein L7	Rpl7	RL7_RAT	*15573548*	*17*	*19640371*	*13*	0.027	1.28
Malate dehydrogenase, cytoplasmic (EC 1.1.1.37) (Cytosolic malate dehydrogenase)	Mdh1 Mdh	MDHC_RAT	*228804074*	*10*	*292827896*	*14*	0.004	1.28
14-3-3 protein zeta/delta (Mitochondrial import stimulation factor S1 subunit) (Protein kinase C inhibitor protein 1) (KCIP-1)	Ywhaz Msfs1	1433Z_RAT	*16977000*	*7*	*21775844*	*15*	0.002	1.28
Elongation factor Tu, mitochondrial	Tufm	EFTU_RAT	*59257027*	*13*	*75190628*	*14*	0.013	1.27
Histone H4 [Cleaved into: Osteogenic growth peptide (OGP)]	Hist1h4b Hist4; Hist1h4m; Hist4h4 H4ft	H4_RAT	*173772828*	*3*	*222989162*	*15*	0.001	1.27
NADPH--cytochrome P450 reductase (CPR) (P450R) (EC 1.6.2.4)	Por	NCPR_RAT	*5943847*	*7*	*7647255*	*20*	0.007	1.27
Delta(3,5)-Delta(2,4)-dienoyl-CoA isomerase, mitochondrial (EC 5.3.3.-)	Ech1	ECH1_RAT	*111356860*	*6*	*142067004*	*12*	0.001	1.27
Long-chain-fatty-acid--CoA ligase 5 (EC 6.2.1.3) (Long-chain acyl-CoA synthetase 5) (LACS 5)	Acsl5 Acs5 Facl5	ACSL5_RAT	*74670106*	*5*	*95496211*	*14*	0.001	1.27
Plasminogen activator inhibitor 1 RNA-binding protein (PAI1 RNA-binding protein 1) (PAI-RBP1) (RDA288) (SERPINE1 mRNA-binding protein 1)	Serbp1 Pairbp1 Rda288	PAIRB_RAT	*2286908*	*16*	*2886708*	*18*	0.031	1.27
Sorbin and SH3 domain-containing protein 2 (Arg-binding protein 2) (ArgBP2) (Arg/Abl-interacting protein 2) (Neural ArgBP2) (nArgBP2) (Sorbin)	Sorbs2 Argbp2	SRBS2_RAT	*35875812*	*6*	*45791962*	*16*	0.002	1.27
Acyl-CoA-binding protein (ACBP) (Diazepam-binding inhibitor) (DBI) (Endozepine) (EP) [Cleaved into: Triakontatetraneuropeptide (TTN); Octadecaneuropeptide (ODN)]	Dbi	ACBP_RAT	*20762087*	*12*	*26368047*	*18*	0.012	1.26
Long-chain specific acyl-CoA dehydrogenase, mitochondrial (LCAD) (EC 1.3.8.8)	Acadl	ACADL_RAT	*107900036*	*8*	*134483394*	*4*	≤0.001	1.25
Neurofilament medium polypeptide (NF-M) (160 kDa neurofilament protein) (Neurofilament 3) (Neurofilament triplet M protein)	Nefm Nef3 Nfm	NFM_RAT	*305495779*	*12*	*379612936*	*11*	0.011	1.25
Sodium/potassium-transporting ATPase subunit alpha-1 (Na(+)/K(+) ATPase alpha-1 subunit) (EC 7.2.2.13) (Sodium pump subunit alpha-1)	Atp1a1	AT1A1_RAT	*57351481*	*6*	*72349439*	*19*	0.006	1.25

Very long-chain specific acyl-CoA dehydrogenase, mitochondrial (VLCAD) (EC 1.3.8.9)	Acadvl Vlcad	ACADV_RAT	*82019269*	*9*	*102295670*	*11*	0.002	1.25
NADH:ubiquinone oxidoreductase subunit A7 (Ndufa7 protein) (RCG37550, isoform CRA_a)	Ndufa7 Ndufa7l rCG_37550	A9UMV9_RAT	*39569667*	*16*	*48774386*	*13*	0.029	1.25
Long-chain fatty acid transport protein 1 (Solute carrier family 27 (Fatty acid transporter), member 1) (Solute carrier family 27 (Fatty acid transporter), member 1, isoform CRA_a)	Slc27a1 rCG_38707	Q6GMM8_RAT	*7027963*	*11*	*8724499*	*11*	0.005	1.24
Enoyl-CoA delta isomerase 1, mitochondrial (EC 5.3.3.8) (3,2-trans-enoyl-CoA isomerase) (Delta(3),Delta(2)-enoyl-CoA isomerase) (D3,D2-enoyl-CoA isomerase) (Dodecenoyl-CoA isomerase)	Eci1 Dci	ECI1_RAT	*173934445*	*9*	*217360265*	*15*	0.008	1.24
Fibrillin 1 (Fibrillin 1, isoform CRA_a)	Fbn1 rCG_27283	G3V9M6_RAT	*32474420*	*14*	*39844980*	*8*	0.011	1.24
Transitional endoplasmic reticulum ATPase (TER ATPase) (EC 3.6.4.6) (15S Mg(2+)-ATPase p97 subunit) (Valosin-containing protein) (VCP)	Vcp	TERA_RAT	*10563372*	*13*	*13075350*	*18*	0.034	1.23
Moesin (Moesin, isoform CRA_a)	Msn rCG_36474	A0A096MK30_RAT	*38065027*	*4*	*48344967*	*28*	0.041	1.23
Trifunctional enzyme subunit alpha, mitochondrial (TP-alpha) [Includes: Long-chain enoyl-CoA hydratase (EC 4.2.1.17); Long chain 3-hydroxyacyl-CoA dehydrogenase (EC 1.1.1.211)]	Hadha	ECHA_RAT	*108964806*	*10*	*134342233*	*13*	0.005	1.23
Heat shock protein HSP 90-beta (Heat shock 84 kDa) (HSP 84) (HSP84)	Hsp90ab1 Hsp84 Hspcb	HS90B_RAT	*47916531*	*7*	*59331315*	*16*	0.006	1.23
Citramalyl-CoA lyase, mitochondrial (EC 4.1.3.25) (Beta-methylmalate synthase) (EC 2.3.3.-) (Citrate lyase subunit beta-like protein, mitochondrial) (Citrate lyase beta-like) (Malate synthase) (EC 2.3.3.9)	Clybl	CLYBL_RAT	*5818855*	*8*	*7202042*	*18*	0.011	1.23
Basic leucine zipper and W2 domain-containing protein 2 (Brain development-related molecule 2)	Bzw2 Bdm2 Hfb2	BZW2_RAT	*27367163*	*9*	*33877630*	*19*	0.014	1.23
Leucine-rich PPR motif-containing protein, mitochondrial (130 kDa leucine-rich protein) (LRP 130) (Leucine rich protein 157) (rLRP157)	Lrpprc Lrp157	LPPRC_RAT	*8218170*	*7*	*10140289*	*14*	0.004	1.23
Creatine kinase S-type, mitochondrial (EC 2.7.3.2) (Basic-type mitochondrial creatine kinase) (Mib-CK) (Sarcomeric mitochondrial creatine kinase) (S-MtCK)	Ckmt2	KCRS_RAT	*93055532*	*16*	*112466628*	*10*	0.030	1.22
NADH-ubiquinone oxidoreductase 75 kDa	Ndufs1	NDUS1_RAT	*48333875*	*8*	*59196389*	*12*	0.006	1.22

subunit, mitochondrial (EC 1.6.99.3) (EC 7.1.1.2)								
AHNAK nucleoprotein	Ahnak	A0A0G2JUA5_RAT	*5538224*	*14*	*6690333*	*11*	0.025	1.22
Phospholemman (FXYD domain-containing ion transport regulator 1) (Sodium/potassium-transporting ATPase subunit FXYD1)	Fxyd1 Plm	PLM_RAT	*56096971*	*7*	*68014386*	*6*	≤0.001	1.21
NADH dehydrogenase [ubiquinone] 1 beta subcomplex subunit 8, mitochondrial (Complex I-ASHI) (NADH-ubiquinone oxidoreductase ASHI subunit)	Ndufb8 Ndufb8_predicted rCG_57427	B2RYS8_RAT	*116488876*	*12*	*141249810*	*14*	0.021	1.21
EH domain-containing protein 3	Ehd3 Ehd2	EHD3_RAT	*18543496*	*5*	*22774632*	*21*	0.018	1.21
NADH:ubiquinone oxidoreductase subunit B3 (RCG22355)	Ndufb3 rCG_22355	D4A4P3_RAT	*126171571*	*12*	*151684214*	*12*	0.021	1.21
Heterogeneous nuclear ribonucleoprotein D-like	Hnrnpdl	A0A0G2KAZ7_RAT	*8854228*	*15*	*10582278*	*10*	0.028	1.21
Lumican (Keratan sulfate proteoglycan lumican) (KSPG lumican)	Lum Lcn Ldc	LUM_RAT	*58608507*	*13*	*70318072*	*11*	0.022	1.21
Aconitate hydratase, mitochondrial (Aconitase) (EC 4.2.1.3) (Citrate hydro-lyase)	Aco2	ACON_RAT	*579367896*	*7*	*697040074*	*7*	≤0.001	1.20
NADH:ubiquinone oxidoreductase subunit B10 (RCG32945)	Ndufb10 rCG_32945	D4A0T0_RAT	*119647546*	*10*	*143490387*	*9*	0.008	1.20
Heat shock protein HSP 90-alpha (Heat shock 86 kDa) (HSP 86) (HSP86)	Hsp90aa1 Hsp86 Hspca	HS90A_RAT	*33041406*	*7*	*40242845*	*20*	0.034	1.20
Sarcoglycan, alpha (Sarcoglycan, alpha (Dystrophin-associated glycoprotein) (Predicted), isoform CRA_a)	Sgca Sgca_predicted rCG_34071	D3ZDQ9_RAT	*20796676*	*5*	*25086379*	*11*	0.001	1.20
Heat shock cognate 71 kDa protein (Heat shock 70 kDa protein 8)	Hspa8 Hsc70 Hsc73	HSP7C_RAT	*51329080*	*6*	*61736955*	*10*	0.001	1.20
Spectrin beta chain	Sptbn1	A0A0G2JZY6_RAT	*11766524*	*7*	*14182537*	*13*	0.007	1.20
Succinate dehydrogenase [ubiquinone] flavoprotein subunit, mitochondrial (EC 1.3.5.1) (Flavoprotein subunit of complex II) (Fp)	Sdha	SDHA_RAT	*119633020*	*4*	*143631747*	*8*	≤0.001	1.20
NADH dehydrogenase (Ubiquinone) 1 beta subcomplex, 9 (NADH dehydrogenase (Ubiquinone) 1 beta subcomplex, 9 (Predicted)) (NADH:ubiquinone oxidoreductase subunit B9) (Ndufb9 protein)	Ndufb9 Ndufb9_predicted rCG_60288	B2RYW3_RAT	*148687496*	*8*	*178052768*	*10*	0.003	1.20
Eukaryotic translation initiation factor 5A-1 (eIF-5A-1) (eIF-5A1) (Eukaryotic initiation factor 5A isoform 1) (eIF-5A) (eIF-4D)	Eif5a	IF5A1_RAT	*3909444*	*14*	*4628547*	*8*	0.027	1.20
Cytochrome b (Complex III subunit 3) (Complex III subunit III) (Cytochrome b-c1 complex subunit 3) (Ubiquinol-cytochrome-c reductase complex cytochrome b subunit)	Mt-Cyb Cob Cytb mt-Cytb Mtcyb	CYB_RAT	*108423775*	*8*	*129441386*	*9*	0.002	1.20
60S ribosomal protein L19	Rpl19	RL19_RAT	*9871351*	*3*	*11860718*	*14*	0.003	1.19

40S ribosomal protein S3a	Cyld LOC100362727 RPS3A	Q6TXJ6_RAT	*12180369*	*8*	*14572029*	*15*	0.017	1.19
Apoptosis-inducing factor 1, mitochondrial (Programmed cell death 8, isoform CRA_a)	Aifm1 Pdcd8 rCG_53222	A0A0G2K7K2_RAT	*10230420*	*7*	*12229880*	*14*	0.014	1.19
Uncharacterized protein		F1LTJ5_RAT	*22358172*	*8*	*26671204*	*17*	0.024	1.19
Medium-chain specific acyl-CoA dehydrogenase, mitochondrial (MCAD) (EC 1.3.8.7)	Acadm	ACADM_RAT	*159857755*	*8*	*189287044*	*8*	0.002	1.18
Palmdelphin	Palmd	PALMD_RAT	*13977611*	*10*	*16498440*	*10*	0.013	1.18
Cytochrome b-c1 complex subunit 2, mitochondrial (Complex III subunit 2) (Core protein II) (Ubiquinol-cytochrome-c reductase complex core protein 2)	Uqcrc2	QCR2_RAT	*158614412*	*12*	*185813688*	*5*	0.014	1.18
Isocitrate dehydrogenase [NAD] subunit alpha, mitochondrial (EC 1.1.1.41) (Isocitric dehydrogenase subunit alpha) (NAD(+)-specific ICDH subunit alpha)	Idh3a	IDH3A_RAT	*116744312*	*5*	*138935589*	*17*	0.017	1.18
Laminin subunit beta-2 (Laminin chain B3) (Laminin-11 subunit beta) (Laminin-14 subunit beta) (Laminin-15 subunit beta) (Laminin-3 subunit beta) (Laminin-4 subunit beta) (Laminin-7 subunit beta) (Laminin-9 subunit beta) (S-laminin subunit beta) (S-LAM beta)	Lamb2	LAMB2_RAT	*15453559*	*9*	*18095855*	*7*	0.004	1.17
Uncharacterized protein		D4A6W6_RAT	*11066533*	*6*	*12935397*	*8*	0.002	1.17
Pyruvate kinase PKM (EC 2.7.1.40) (Pyruvate kinase muscle isozyme)	Pkm Pkm2 Pykm	KPYM_RAT	*331632330*	*6*	*387978412*	*11*	0.006	1.17
Isocitrate dehydrogenase [NAD] subunit beta, mitochondrial (Isocitric dehydrogenase subunit beta) (NAD(+)-specific ICDH subunit beta)	Idh3B	IDH3B_RAT	*41704638*	*8*	*48630221*	*9*	0.007	1.17
Peroxiredoxin 3 (Thioredoxin-dependent peroxide reductase, mitochondrial)	Prdx3 rCG_57612	G3V7I0_RAT	*262272674*	*8*	*304330623*	*7*	0.005	1.16
Mitochondrial import receptor subunit TOM22 homolog (rTOM22) (Translocase of outer membrane 22 kDa subunit homolog)	Tomm22 Tom22	TOM22_RAT	*140791982*	*7*	*163990206*	*11*	0.010	1.16
NADH dehydrogenase [ubiquinone] 1 alpha subcomplex subunit 11 (Complex I-B14.7) (CI-B14.7) (NADH-ubiquinone oxidoreductase subunit B14.7)	Ndufa11	NDUAB_RAT	*117713138*	*6*	*134508318*	*7*	0.003	1.14
Glutamate dehydrogenase 1, mitochondrial (GDH 1) (EC 1.4.1.3) (Memory-related gene 2 protein) (MRG-2)	Glud1 Glud	DHE3_RAT	*27262527*	*4*	*23314448*	*11*	0.002	-1.18
Myosin-binding protein C, cardiac-type (Cardiac MyBP-C) (C-protein, cardiac muscle isoform)	Mybpc3	MYPC_RAT	*122240540*	*11*	*104428084*	*15*	0.021	-1.18
3-oxo-5-beta-steroid 4-dehydrogenase (EC 1.3.1.3)	Akr1d1	AK1D1_RAT	*74456095*	*17*	*62121341*	*12*	0.020	-1.19

(Aldo-keto reductase family 1 member D1) (Delta(4)-3-ketosteroid 5-beta-reductase) (Delta(4)-3-oxosteroid 5-beta-reductase)								
Aldose reductase-related protein 1 (EC 1.1.1.21) (Aldehyde reductase) (Aldo-keto reductase family 1 member B7) (Aldose reductase-like protein AKR1B14)	Akr1b7 Akr1b14	ALD1_RAT	*74456095*	*17*	*62121341*	*12*	0.020	-1.19
Voltage-dependent anion-selective channel protein 2 (VDAC-2) (B36-VDAC) (Outer mitochondrial membrane protein porin 2)	Vdac2	VDAC2_RAT	*359743034*	*14*	*302419463*	*17*	0.031	-1.20
Glypican-1 (HSPG M12) [Cleaved into: Secreted glypican-1]	Gpc1	GPC1_RAT	*16480007*	*17*	*13546811*	*11*	0.011	-1.21
Phosphoglycerate mutase 2 (EC 5.4.2.11) (EC 5.4.2.4) (BPG-dependent PGAM 2) (Muscle-specific phosphoglycerate mutase) (Phosphoglycerate mutase isozyme M) (PGAM-M)	Pgam2	PGAM2_RAT	*181191048*	*16*	*148579334*	*17*	0.019	-1.22
ES1 protein homolog, mitochondrial		ES1_RAT	*73924744*	*17*	*59412836*	*7*	0.003	-1.24
Enoyl-CoA hydratase, mitochondrial (EC 4.2.1.17) (Enoyl-CoA hydratase 1) (Short-chain enoyl-CoA hydratase) (SCEH)	Echs1	ECHM_RAT	*91350440*	*16*	*74761837*	*22*	0.046	-1.24
Myomesin 2	Myom2 rCG_43076	G3V7K1_RAT	*38061733*	*14*	*30824497*	*15*	0.014	-1.24
Cytochrome c oxidase subunit 5B, mitochondrial (Cytochrome c oxidase polypeptide Vb) (Cytochrome c oxidase subunit VIA*)	Cox5b	COX5B_RAT	*192194389*	*12*	*157063543*	*24*	0.041	-1.25
Propionyl coenzyme A carboxylase, beta polypeptide (Propionyl coenzyme A carboxylase, beta polypeptide, isoform CRA_b) (Propionyl-CoA carboxylase beta chain, mitochondrial)	Pccb rCG_25130	Q68FZ8_RAT	*12626766*	*18*	*10075119*	*14*	0.012	-1.25
2-oxoisovalerate dehydrogenase subunit beta, mitochondrial (EC 1.2.4.4) (Branched-chain alpha-keto acid dehydrogenase E1 component beta chain) (BCKDE1B) (BCKDH E1-beta)	Bckdhb	ODBB_RAT	*36314045*	*9*	*29690203*	*26*	0.031	-1.25
Atypical kinase COQ8A, mitochondrial (EC 2.7.-.-) (Chaperone activity of bc1 complex-like) (Chaperone-ABC1-like) (Coenzyme Q protein 8A) (aarF domain-containing protein kinase 3)	Coq8a Adck3 Cabc1	COQ8A_RAT	*56826673*	*17*	*45920797*	*21*	0.039	-1.26
D-dopachrome decarboxylase (EC 4.1.1.84) (D-dopachrome tautomerase)	Ddt	DOPD_RAT	*55850295*	*25*	*43643389*	*18*	0.025	-1.28
Solute carrier family 25 member 13	Slc25a13	F1LZW6_RAT	*39142675*	*10*	*30756744*	*14*	0.001	-1.28
Uncharacterized protein		D4A647_RAT	*2115265*	*19*	*1651398*	*20*	0.014	-1.29
Creatine kinase M-type (Creatine kinase, muscle)	Ckm rCG_54105	A0A0G2JSP8_RAT	*550454545*	*15*	*430406122*	*16*	0.005	-1.29

Propionyl-CoA carboxylase alpha chain, mitochondrial (PCCase subunit alpha) (EC 6.4.1.3) (Propanoyl-CoA:carbon dioxide ligase subunit alpha)	Pcca	PCCA_RAT	*11077243*	*18*	*8602750*	*18*	0.008	-1.29
Actin, cytoplasmic 1 (Beta-actin) [Cleaved into: Actin, cytoplasmic 1, N-terminally processed]	Actb	ACTB_RAT	*413643032*	*13*	*324093236*	*23*	0.014	-1.30
Glutathione S-transferase, mu 6-like	Gstm6l	A0A0G2JZ09_RAT	*12321808*	*24*	*9313511*	*15*	0.010	-1.31
4-trimethylaminobutyraldehyde dehydrogenase (TMABADH) (EC 1.2.1.47) (Aldehyde dehydrogenase family 9 member A1) (EC 1.2.1.3)	Aldh9a1	AL9A1_RAT	*22203216*	*21*	*16941450*	*21*	0.008	-1.32
NADH dehydrogenase (Ubiquinone) Fe-S protein 8 (Predicted), isoform CRA_a (NADH:ubiquinone oxidoreductase core subunit S8) (Ndufs8 protein)	Ndufs8 Ndufs8_predicted rCG_47133	B0BNE6_RAT	*43775792*	*37*	*32333873*	*16*	0.029	-1.33
Actin, aortic smooth muscle	Acta2	A0A0G2K4M6_RAT	*507545976*	*13*	*394499398*	*29*	0.028	-1.33
Prostaglandin reductase 1 (PRG-1) (EC 1.3.1.-) (15-oxoprostaglandin 13-reductase) (EC 1.3.1.48) (Dithiolethione-inducible gene 1 protein) (D3T-inducible gene 1 protein) (DIG-1) (NADP-dependent leukotriene B4 12-hydroxydehydrogenase) (EC 1.3.1.74)	Ptgr1 Dig1 Ltb4dh	PTGR1_RAT	*8128407*	*27*	*6064182*	*20*	0.020	-1.33
Dihydrolipoamide acetyltransferase component of pyruvate dehydrogenase complex (EC 2.3.1.-)	Pdhx	A0A0G2JZH8_RAT	*23681246*	*15*	*18250713*	*26*	0.025	-1.34
Transcription initiation factor TFIID subunit 6 (Transcription initiation factor TFIID 70 kDa subunit) (TAF(II)70) (TAFII70) (Transcription initiation factor TFIID 80 kDa subunit) (TAF(II)80) (TAFII-80) (TAFII80) (p80)	Taf6 Taf2e	TAF6_RAT	*8288069*	*17*	*6248834*	*19*	0.005	-1.34
Myozenin 2 (Myozenin 2 (Predicted), isoform CRA_b)	Myoz2 Myoz2_predicted rCG_28510	D3ZX18_RAT	*74894703*	*24*	*56640375*	*29*	0.021	-1.34
Heat shock protein beta-1 (HspB1) (Heat shock 27 kDa protein) (HSP 27)	Hspb1 Hsp27	HSPB1_RAT	*52367625*	*8*	*39662474*	*22*	0.005	-1.35
Aldehyde dehydrogenase, mitochondrial (EC 1.2.1.3) (ALDH class 2) (ALDH-E2) (ALDH1)	Aldh2	ALDH2_RAT	*45588834*	*15*	*33620414*	*10*	≤0.001	-1.35
Annexin A5 (Anchorin CII) (Annexin V) (Annexin-5) (Calphobindin I) (CBP-I) (Endonexin II) (Lipocortin V) (Placental anticoagulant protein 4) (PP4) (Placental anticoagulant protein I) (PAP-I) (Thromboplastin inhibitor) (Vascular anticoagulant-alpha) (VAC-alpha)	Anxa5 Anx5	ANXA5_RAT	*52708583*	*25*	*38890303*	*20*	0.013	-1.36
40S ribosomal protein S3 (EC 4.2.99.18)	Rps3	RS3_RAT	*18972696*	*9*	*14540543*	*32*	0.028	-1.37

Branched-chain-amino-acid aminotransferase, mitochondrial (BCAT(m)) (EC 2.6.1.42)	Bcat2 Bcatm Eca40	BCAT2_RAT	*21303576*	*21*	*15600033*	*20*	0.004	-1.38
Short-chain specific acyl-CoA dehydrogenase, mitochondrial (SCAD) (EC 1.3.8.1) (Butyryl-CoA dehydrogenase)	Acads	ACADS_RAT	*102637147*	*19*	*76078963*	*26*	0.015	-1.38
2-oxoglutarate dehydrogenase, mitochondrial (EC 1.2.4.2) (2-oxoglutarate dehydrogenase complex component E1) (OGDC-E1) (Alpha-ketoglutarate dehydrogenase)	Ogdh Ogdhl	ODO1_RAT	*39616976*	*13*	*28812119*	*15*	≤0.001	-1.38
Histidine triad nucleotide binding protein 2 (Predicted), isoform CRA_a (Histidine triad nucleotide-binding protein 2)	Hint2 Hint2_predicted rCG_54875	D4AB01_RAT	*10739362*	*21*	*8064611*	*42*	0.046	-1.42
Glucagon-like peptide 2 receptor (GLP-2 receptor) (GLP-2-R) (GLP-2R)	Glp2r	GLP2R_RAT	*25590216*	*30*	*17839643*	*22*	0.008	-1.43
Tripartite motif-containing protein 72 (Mitsugumin-53) (Mg53)	Trim72 Mg53	TRI72_RAT	*6183900*	*21*	*4310379*	*19*	0.001	-1.44
Isocitrate dehydrogenase [NADP], mitochondrial (IDH) (EC 1.1.1.42) (ICD-M) (IDP) (NADP(+)-specific ICDH) (Oxalosuccinate decarboxylase)	Idh2	IDHP_RAT	*260632596*	*13*	*187914432*	*36*	0.011	-1.46
ATP-dependent 6-phosphofructokinase (ATP-PFK) (Phosphofructokinase) (EC 2.7.1.11) (Phosphohexokinase)	Pfkm	A0A0G2KBC7_RAT	*5373693*	*28*	*3797132*	*34*	0.025	-1.47
LIM domain-binding 3	Ldb3	A0A096MJ01_RAT	*84387670*	*15*	*58727923*	*30*	0.006	-1.49
Myosin-4	Myh4 Myh2	F1LMU0_RAT	*1399408891*	*36*	*941403190*	*24*	0.007	-1.50
Myosin-7 (Myosin heavy chain 7) (Myosin heavy chain slow isoform) (MyHC-slow) (Myosin heavy chain, cardiac muscle beta isoform) (MyHC-beta)	Myh7	MYH7_RAT	*212881703*	*34*	*136971592*	*21*	0.002	-1.54
Enoyl-[acyl-carrier-protein] reductase, mitochondrial (EC 1.3.1.104) (2-enoyl thioester reductase) (Nuclear receptor-binding factor 1) (NRBF-1)	Mecr Nrbf1	MECR_RAT	*822847*	*41*	*547069*	*50*	0.029	-1.61
Peptidyl-prolyl cis-trans isomerase (PPIase) (EC 5.2.1.8)	LOC100911252	A0A0G2K2E4_RAT	*3140460*	*35*	*2076420*	*44*	0.040	-1.64
Prohibitin-2 (B-cell receptor-associated protein BAP37) (BAP-37)	Phb2 Bcap37	PHB2_RAT	*26250521*	*13*	*16637134*	*34*	0.001	-1.65
40S ribosomal protein S9	Rps9	RS9_RAT	*3780381*	*59*	*2263795*	*50*	0.032	-1.75
Erlin-2 (Endoplasmic reticulum lipid raft-associated protein 2) (Stomatin-prohibitin-flotillin-HflC/K domain-containing protein 2) (SPFH domain-containing protein 2)	Erlin2 Spfh2	ERLN2_RAT	*2363741*	*19*	*1535462*	*40*	0.045	-1.78
Superoxide dismutase [Mn], mitochondrial (EC 1.15.1.1)	Sod2	SODM_RAT	*31144681*	*35*	*19768666*	*56*	0.033	-1.82

Cytoplasmic dynein 1 heavy chain 1 (RCG27764, isoform CRA_a)	Dync1h1 rCG_27764	M0R9X8_RAT	*347572*	*57*	*186920*	*36*	0.003	-1.89
Glycogenin-1 (GN-1) (GN1) (EC 2.4.1.186)	Gyg1 Gyg	GLYG_RAT	*6994496*	*41*	*4440418*	*70*	0.040	-1.89
Isovaleryl-CoA dehydrogenase, mitochondrial (IVD) (EC 1.3.8.4)	Ivd	IVD_RAT	*56599875*	*22*	*32614222*	*42*	0.004	-1.90
Electron transfer flavoprotein subunit alpha, mitochondrial (Alpha-ETF)	Etfa	ETFA_RAT	*21862626*	*77*	*10758456*	*16*	0.001	-1.94
Cytochrome b-c1 complex subunit Rieske, mitochondrial (EC 7.1.1.8) (Complex III subunit 5) (Cytochrome b-c1 complex subunit 5) (Liver regeneration-related protein LRRGT00195) (Rieske iron-sulfur protein) (RISP) (Rieske protein UQCRFS1) (Ubiquinol-cytochrome c reductase iron-sulfur subunit) [Cleaved into: Cytochrome b-c1 complex subunit 9 (Su9) (Subunit 9) (8 kDa subunit 9) (Complex III subunit IX) (Cytochrome b-c1 complex subunit 11) (Ubiquinol-cytochrome c reductase 8 kDa protein)]	Uqcrfs1	UCRI_RAT	*40492785*	*40*	*21733079*	*36*	0.001	-1.95
Methylcrotonoyl-CoA carboxylase beta chain, mitochondrial (MCCase subunit beta) (EC 6.4.1.4) (3-methylcrotonyl-CoA carboxylase 2) (3-methylcrotonyl-CoA carboxylase non-biotin-containing subunit) (3-methylcrotonyl-CoA:carbon dioxide ligase subunit beta)	Mccc2	MCCB_RAT	*2609545*	*58*	*1460929*	*73*	0.046	-2.18
Vinculin (Metavinculin)	Vcl	VINC_RAT	*2696005*	*68*	*1287417*	*56*	0.034	-2.32
Interferon-inducible double-stranded RNA-dependent protein kinase activator A (Protein activator of the interferon-induced protein kinase) (Protein kinase, interferon-inducible double-stranded RNA-dependent activator)	Prkra	PRKRA_RAT	*72141*	*171*	*26215*	*45*	0.026	-2.64
Adenylosuccinate synthetase isozyme 1 (AMPSase 1) (AdSS 1) (EC 6.3.4.4) (Adenylosuccinate synthetase, basic isozyme) (Adenylosuccinate synthetase, muscle isozyme) (M-type adenylosuccinate synthetase) (IMP--aspartate ligase 1)	Adssl1 ADSS1 ADSSL1 rCG_27771	M0R629_RAT	*45531*	*29*	*16636*	*27*	0.017	-2.80
Keratin, type I cytoskeletal 10 (Cytokeratin-10) (CK-10) (Keratin-10) (K10) (Type I keratin Ka10)	Krt10 Ka10	K1C10_RAT	*939301*	*94*	*419557*	*101*	0.011	-2.90
NADH dehydrogenase [ubiquinone] 1 alpha subcomplex subunit 10, mitochondrial (Complex I-42kD) (CI-42kD) (NADH-	Ndufa10	NDUAA_RAT	*791094*	*114*	*265487*	*49*	0.038	-3.15

ubiquinone oxidoreductase 42 kDa subunit)								
Galectin-1 (Gal-1) (14 kDa lectin) (Beta-galactoside-binding lectin L-14-I) (Galaptin) (Lactose-binding lectin 1) (Lectin galactoside-binding soluble 1) (RL 14.5) (S-Lac lectin 1)	Lgals1	LEG1_RAT	*35144549*	*33*	*15042046*	*86*	0.005	-3.23
Complement C3	C3	M0RBJ7_RAT	*2061209*	*34*	*1003265*	*77*	0.031	-3.28
Protein disulfide-isomerase (PDI) (EC 5.3.4.1) (Cellular thyroid hormone-binding protein) (Prolyl 4-hydroxylase subunit beta)	P4hb Pdia1	PDIA1_RAT	*8136997*	*84*	*2874527*	*67*	0.001	-3.40
Dihydrolipoyllysine-residue acetyltransferase component of pyruvate dehydrogenase complex, mitochondrial (EC 2.3.1.12) (70 kDa mitochondrial autoantigen of primary biliary cirrhosis) (PBC) (Dihydrolipoamide acetyltransferase component of pyruvate dehydrogenase complex) (Pyruvate dehydrogenase complex component E2) (PDC-E2) (PDCE2)	Dlat	ODP2_RAT	*222948*	*252*	*55631*	*22*	0.022	-3.47
Pyridine nucleotide-disulfide oxidoreductase domain-containing protein 2 (EC 1.-.-.-)	Pyroxd2	PYRD2_RAT	*1405732*	*88*	*517493*	*98*	0.013	-5.32
Dynein light chain roadblock-type 1 (Bithoraxoid-like protein) (BLP) (robl/LC7-like protein) (Dynein light chain 2A, cytoplasmic) (Dynein-associated protein Km23)	Dynlrb1 Dncl2a Dnlc2a	DLRB1_RAT	*11777318*	*65*	*5063311*	*102*	0.024	-6.43
Dihydrolipoamide acetyltransferase component of pyruvate dehydrogenase complex (EC 2.3.1.-)	Dbt rCG_28617	B2GV15_RAT	*598046*	*61*	*262677*	*109*	0.039	-9.53

Reactome pathways

Pathway identifier	**Pathway name**	**#Entities found**	***#Interactors found***	**Submitted entities found**
R-HSA-1430728	**Metabolism**	**29**	***9***	**Mecr;Ndufa1;Ndufa2;Aldh7a1;Adssl1;Cs;Ndufa10;Acaa2;Uqcrfs1;Dbt;Pgk1;Prelp;Gyg1;Hibch;Rps9;Got1;Mtnd4;Bgn;Etfa;Hprt1;Ivd;Gpd1;Mccc2;Etfdh;Me3;Dlat;Cd36**
R-HSA-168256	**Immune System**	**14**	***23***	**Lama5;C3;Ctsd;Pafah1b2;P4hb;Cd36;Prdx6;Sod2;Dync1h1;Vcl;Gyg1**
R-HSA-1643685	**Disease**	**12**	***11***	**Rps9;Mpi;Mccc2;Bgn;Erlin2;Prelp;Slc25a4;Cd36;Sod2;Vcl;Gyg1**
R-HSA-162582	**Signal Transduction**	**12**	***13***	**C3;Lama5;Ctsd;Rdx;Cyct;Dbt;Lamc1;Dlat;P4hb;Dync1h1;Vcl**
R-HSA-1428517	**The citric acid (TCA) cycle and respiratory electron transport**	**10**	***3***	**Cs;Ndufa10;Uqcrfs1;Mtnd4;Ndufa1;Etfdh;Ndufa2;Me3;Dlat;Etfa**
R-HSA-392499	**Metabolism of proteins**	**9**	***11***	**C3;Ctsd;Rps9;Mpi;Vdac3;Lamc1;P4hb;Lgals1;Dync1h1**
R-HSA-397014	**Muscle contraction**	**8**	***1***	**Tmod1;Myl2;Tpm1;Myl3;Casq2;Tnni3;Vcl;Myh6;Myh7**
R-HSA-6798695	**Neutrophil degranulation**	**8**	***0***	**C3;Ctsd;Pafah1b2;Cd36;Prdx6;Dync1h1;Vcl;Gyg1**

R-HSA-71291	**Metabolism of amino acids and derivatives**	**8**	***0***	**Hibch;Rps9;Got1;Ivd;Dbt;Mccc2;Aldh7a1;Dlat**
R-HSA-449147	**Signaling by Interleukins**	**8**	***7***	**Lama5;P4hb;Cd36;Sod2;Vcl**
R-HSA-168249	**Innate Immune System**	**8**	***11***	**C3;Ctsd;Pafah1b2;Cd36;Prdx6;Dync1h1;Vcl;Gyg1**
R-HSA-1280215	**Cytokine Signaling in Immune system**	**8**	***11***	**Lama5;P4hb;Cd36;Sod2;Vcl**
R-HSA-611105	**Respiratory electron transport**	**7**	***1***	**Ndufa10;Uqcrfs1;Mtnd4;Ndufa1;Etfdh;Ndufa2;Etfa**
R-HSA-163200	**Respiratory electron transport, ATP synthesis by chemiosmotic coupling, and heat production by uncoupling proteins.**	**7**	***1***	**Ndufa10;Uqcrfs1;Mtnd4;Ndufa1;Etfdh;Ndufa2;Etfa**
R-HSA-382551	**Transport of small molecules**	**7**	***0***	**Mb;Vdac3;Erlin2;Casq2;Slc25a4;P4hb;Phb2**
R-HSA-597592	**Post-translational protein modification**	**7**	***7***	**C3;Mpi;Vdac3;Lamc1;P4hb;Lgals1;Dync1h1**
R-HSA-390522	**Striated Muscle Contraction**	**6**	***0***	**Tmod1;Myl2;Tpm1;Myl3;Tnni3;Myh6;Myh7**
R-HSA-71387	**Metabolism of carbohydrates**	**6**	***1***	**Got1;Bgn;Pgk1;Prelp;Gyg1**
R-HSA-2262752	**Cellular responses to stress**	**6**	***2***	**Hist1h1b;P4hb;Prdx6;Sod2;Dync1h1;Cryab**
R-HSA-8953897	**Cellular responses to external stimuli**	**6**	***4***	**Hist1h1b;P4hb;Prdx6;Sod2;Dync1h1;Cryab**
R-HSA-1266738	**Developmental Biology**	**6**	***9***	**Rps9;Rdx;Lamc1;Krt10;Cd36**
R-HSA-1474244	**Extracellular matrix organization**	**5**	***9***	**Lama5;Ctsd;Bgn;Lamc1;P4hb**
R-HSA-1852241	**Organelle biogenesis and maintenance**	**5**	***4***	**Dbt;Immt;Sod2;Dynlrb1;Dync1h1**
R-HSA-109582	**Hemostasis**	**5**	***5***	**Wdr1;Cyct;Tagln2;Cd36;Vcl**
R-HSA-556833	**Metabolism of lipids**	**5**	***3***	**Acaa2;Mecr;Gpd1;Cd36**
R-HSA-74160	**Gene expression (Transcription)**	**5**	***12***	**Prkra;Sod2;Hspd1**
R-HSA-70895	**Branched-chain amino acid catabolism**	**4**	***0***	**Hibch;Ivd;Dbt;Mccc2**
R-HSA-8957275	**Post-translational protein phosphorylation**	**4**	***0***	**C3;Lamc1;P4hb;Lgals1**
R-HSA-6799198	**Complex I biogenesis**	**4**	***1***	**Ndufa10;Mtnd4;Ndufa1;Ndufa2**
R-HSA-114608	**Platelet degranulation**	**4**	***0***	**Wdr1;Tagln2;Cd36;Vcl**
R-HSA-76005	**Response to elevated platelet cytosolic Ca2+**	**4**	***0***	**Wdr1;Tagln2;Cd36;Vcl**
R-HSA-381426	**Regulation of Insulin-like Growth Factor (IGF) transport and uptake by Insulin-like Growth Factor Binding Proteins (IGFBPs)**	**4**	***0***	**C3;Lamc1;P4hb;Lgals1**
R-HSA-6785807	**Interleukin-4 and Interleukin-13 signaling**	**4**	***0***	**Lama5;Cd36**
R-HSA-76002	**Platelet activation, signaling and aggregation**	**4**	***3***	**Wdr1;Tagln2;Cd36;Vcl**
R-HSA-5653656	**Vesicle-mediated transport**	**4**	***5***	**Hpx;Pafah1b2;Cd36;Dync1h1**
R-HSA-1280218	**Adaptive Immune System**	**4**	***12***	**C3;Ctsd;Cd36;Dync1h1**
R-HSA-212436	**Generic Transcription Pathway**	**4**	***10***	**Sod2;Hspd1**
R-HSA-73857	**RNA Polymerase II Transcription**	**4**	***10***	**Sod2;Hspd1**
R-HSA-1268020	**Mitochondrial protein import**	**3**	***0***	**Cs;Slc25a4;Hspd1**
R-HSA-3000178	**ECM proteoglycans**	**3**	***0***	**Lama5;Bgn;Lamc1**
R-HSA-70263	**Gluconeogenesis**	**3**	***1***	**Got1;Pgk1**
R-HSA-71406	**Pyruvate metabolism and Citric Acid (TCA) cycle**	**3**	***2***	**Cs;Me3;Dlat**
R-HSA-1474228	**Degradation of the extracellular matrix**	**3**	***0***	**Lama5;Ctsd;Lamc1**
R-HSA-70326	**Glucose metabolism**	**3**	***1***	**Got1;Pgk1**
R-HSA-3781865	**Diseases of glycosylation**	**3**	***0***	**Mpi;Bgn;Prelp**
R-HSA-9609507	**Protein localization**	**3**	***0***	**Cs;Slc25a4;Hspd1**
R-HSA-3299685	**Detoxification of Reactive Oxygen Species**	**3**	***0***	**P4hb;Prdx6;Sod2**
R-HSA-9020591	**Interleukin-12 signaling**	**3**	***3***	**P4hb;Sod2**
R-HSA-5617833	**Cilium Assembly**	**3**	***1***	**Dbt;Dynlrb1;Dync1h1**
R-HSA-447115	**Interleukin-12 family signaling**	**3**	***5***	**P4hb;Sod2**
R-HSA-9006931	**Signaling by Nuclear Receptors**	**3**	***2***	**Ctsd;Dlat**
R-HSA-422475	**Axon guidance**	**3**	***5***	**Rps9;Rdx;Lamc1**
R-HSA-8949215	**Mitochondrial calcium ion transport**	**2**	***0***	**Vdac3;Phb2**
R-HSA-3000157	**Laminin interactions**	**2**	***0***	**Lama5;Lamc1**
R-HSA-8869496	**TFAP2A acts as a transcriptional repressor during retinoic acid induced cell differentiation**	**2**	***0***	**Hspd1**

R-HSA-3560782	**Diseases associated with glycosaminoglycan metabolism**	**2**	***0***	**Bgn;Prelp**
R-HSA-380320	**Recruitment of NuMA to mitotic centrosomes**	**2**	***0***	**Dbt;Dync1h1**
R-HSA-8854518	**AURKA Activation by TPX2**	**2**	***0***	**Dbt;Dync1h1**
R-HSA-71403	**Citric acid cycle (TCA cycle)**	**2**	***0***	**Cs;Me3**
R-HSA-389661	**Glyoxylate metabolism and glycine degradation**	**2**	***0***	**Dbt;Dlat**
R-HSA-8874081	**MET activates PTK2 signaling**	**2**	***0***	**Lama5;Lamc1**
R-HSA-380270	**Recruitment of mitotic centrosome proteins and complexes**	**2**	***0***	**Dbt;Dync1h1**
R-HSA-9615017	**FOXO-mediated transcription of oxidative stress, metabolic and neuronal genes**	**2**	***2***	**Sod2**
R-HSA-5578775	**Ion homeostasis**	**2**	***0***	**Casq2;Tnni3**
R-HSA-6811436	**COPI-independent Golgi-to-ER retrograde traffic**	**2**	***2***	**Pafah1b2;Dync1h1**
R-HSA-70171	**Glycolysis**	**2**	***0***	**Pgk1**
R-HSA-445355	**Smooth Muscle Contraction**	**2**	***1***	**Tpm1;Vcl**
R-HSA-380259	**Loss of Nlp from mitotic centrosomes**	**2**	***0***	**Dbt;Dync1h1**
R-HSA-380284	**Loss of proteins required for interphase microtubule organization from the centrosome**	**2**	***0***	**Dbt;Dync1h1**
R-HSA-8875878	**MET promotes cell motility**	**2**	***0***	**Lama5;Lamc1**
R-HSA-380287	**Centrosome maturation**	**2**	***0***	**Dbt;Dync1h1**
R-HSA-446353	**Cell-extracellular matrix interactions**	**2**	***1***	**Flnc;Fermt2**
R-HSA-5620912	**Anchoring of the basal body to the plasma membrane**	**2**	***0***	**Dbt;Dync1h1**
R-HSA-77289	**Mitochondrial Fatty Acid Beta-Oxidation**	**2**	***0***	**Acaa2;Mecr**
R-HSA-2173782	**Binding and Uptake of Ligands by Scavenger Receptors**	**2**	***0***	**Hpx;Cd36**
R-HSA-2565942	**Regulation of PLK1 Activity at G2/M Transition**	**2**	***1***	**Dbt;Dync1h1**
R-HSA-1630316	**Glycosaminoglycan metabolism**	**2**	***0***	**Bgn;Prelp**
R-HSA-3000171	**Non-integrin membrane-ECM interactions**	**2**	***2***	**Lama5;Lamc1**
R-HSA-5576891	**Cardiac conduction**	**2**	***0***	**Casq2;Tnni3**
R-HSA-2132295	**MHC class II antigen presentation**	**2**	***1***	**Ctsd;Dync1h1**
R-HSA-68877	**Mitotic Prometaphase**	**2**	***0***	**Dbt;Dync1h1**
R-HSA-8856688	**Golgi-to-ER retrograde transport**	**2**	***2***	**Pafah1b2;Dync1h1**
R-HSA-5668914	**Diseases of metabolism**	**2**	***0***	**Mccc2;Gyg1**
R-HSA-15869	**Metabolism of nucleotides**	**2**	***0***	**Adssl1;Hprt1**
R-HSA-446728	**Cell junction organization**	**2**	***2***	**Flnc;Fermt2**
R-HSA-8950505	**Gene and protein expression by JAK-STAT signaling after Interleukin-12 stimulation**	**2**	***3***	**Sod2**
R-HSA-1592230	**Mitochondrial biogenesis**	**2**	***3***	**Immt;Sod2**
R-HSA-6806834	**Signaling by MET**	**2**	***2***	**Lama5;Lamc1**
R-HSA-381340	**Transcriptional regulation of white adipocyte differentiation**	**2**	***3***	**Cd36**
R-HSA-1989781	**PPARA activates gene expression**	**2**	***0***	**Cd36**
R-HSA-6811442	**Intra-Golgi and retrograde Golgi-to-ER traffic**	**2**	***2***	**Pafah1b2;Dync1h1**
R-HSA-6791226	**Major pathway of rRNA processing in the nucleolus and cytosol**	**2**	***0***	**Rps9;Dbt**
R-HSA-400206	**Regulation of lipid metabolism by Peroxisome proliferator-activated receptor alpha (PPARalpha)**	**2**	***0***	**Cd36**
R-HSA-5358351	**Signaling by Hedgehog**	**2**	***0***	**Rdx;P4hb**
R-HSA-1500931	**Cell-Cell communication**	**2**	***2***	**Flnc;Fermt2**
R-HSA-8868773	**rRNA processing in the nucleus and cytosol**	**2**	***0***	**Rps9;Dbt**
R-HSA-68886	**M Phase**	**2**	***0***	**Dbt;Dync1h1**
R-HSA-69275	**G2/M Transition**	**2**	***1***	**Dbt;Dync1h1**
R-HSA-453274	**Mitotic G2-G2/M phases**	**2**	***1***	**Dbt;Dync1h1**
R-HSA-9614085	**FOXO-mediated transcription**	**2**	***2***	**Sod2**
R-HSA-72312	**rRNA processing**	**2**	***0***	**Rps9;Dbt**
R-HSA-8978868	**Fatty acid metabolism**	**2**	***1***	**Acaa2;Mecr**
R-HSA-446203	**Asparagine N-linked glycosylation**	**2**	***0***	**Mpi;Dync1h1**

R-HSA-373760	**L1CAM interactions**	**2**	***2***	**Rdx;Lamc1**
R-HSA-8864260	**Transcriptional regulation by the AP-2 (TFAP2) family of transcription factors**	**2**	***4***	**Hspd1**
R-HSA-8863678	**Neurodegenerative Diseases**	**2**	***5***	**Sod2**
R-HSA-8862803	**Deregulated CDK5 triggers multiple neurodegenerative pathways in Alzheimer's disease models**	**2**	***5***	**Sod2**
R-HSA-9018519	**Estrogen-dependent gene expression**	**2**	***1***	**Ctsd**
R-HSA-8939211	**ESR-mediated signaling**	**2**	***2***	**Ctsd**
R-HSA-5663202	**Diseases of signal transduction**	**2**	***1***	**Erlin2;Vcl**
R-HSA-69278	**Cell Cycle, Mitotic**	**2**	***1***	**Dbt;Dync1h1**
R-HSA-5663205	**Infectious disease**	**2**	***5***	**Rps9;Slc25a4**
R-HSA-199991	**Membrane Trafficking**	**2**	***5***	**Pafah1b2;Dync1h1**
R-HSA-1640170	**Cell Cycle**	**2**	***7***	**Dbt;Dync1h1**
R-HSA-9006934	**Signaling by Receptor Tyrosine Kinases**	**2**	***6***	**Lama5;Lamc1**
R-HSA-388396	**GPCR downstream signalling**	**2**	***1***	**C3;Cyct**
R-HSA-8953854	**Metabolism of RNA**	**2**	***1***	**Rps9;Dbt**
R-HSA-372790	**Signaling by GPCR**	**2**	***1***	**C3;Cyct**
R-HSA-4043916	**Defective MPI causes MPI-CDG (CDG-1b)**	**1**	***0***	**Mpi**
R-HSA-3814836	**Glycogen storage disease type XV (GYG1)**	**1**	***0***	**Gyg1**
R-HSA-5357609	**Glycogen storage disease type II (GAA)**	**1**	***0***	**Gyg1**
R-HSA-3828062	**Glycogen storage disease type 0 (muscle GYS1)**	**1**	***0***	**Gyg1**
R-HSA-180897	**Vpr-mediated induction of apoptosis by mitochondrial outer membrane permeabilization**	**1**	***0***	**Slc25a4**
R-HSA-8981607	**Intracellular oxygen transport**	**1**	***0***	**Mb**
R-HSA-3656244	**Defective B4GALT1 causes B4GALT1-CDG (CDG-2d)**	**1**	***0***	**Prelp**
R-HSA-3595174	**Defective CHST14 causes EDS, musculocontractural type**	**1**	***0***	**Bgn**
R-HSA-3656243	**Defective ST3GAL3 causes MCT12 and EIEE15**	**1**	***0***	**Prelp**
R-HSA-3656225	**Defective CHST6 causes MCDC1**	**1**	***0***	**Prelp**
R-HSA-3595172	**Defective CHST3 causes SEDCJD**	**1**	***0***	**Bgn**
R-HSA-426486	**Small interfering RNA (siRNA) biogenesis**	**1**	***0***	**Prkra**
R-HSA-3595177	**Defective CHSY1 causes TPBS**	**1**	***0***	**Bgn**
R-HSA-3785653	**Myoclonic epilepsy of Lafora**	**1**	***0***	**Gyg1**
R-HSA-2022923	**Dermatan sulfate biosynthesis**	**1**	***0***	**Bgn**
R-HSA-1236973	**Cross-presentation of particulate exogenous antigens (phagosomes)**	**1**	***0***	**Cd36**
R-HSA-173736	**Alternative complement activation**	**1**	***0***	**C3**
R-HSA-140342	**Apoptosis induced DNA fragmentation**	**1**	***0***	**Hist1h1b**
R-HSA-8949664	**Processing of SMDT1**	**1**	***0***	**Phb2**
R-HSA-4420332	**Defective B3GALT6 causes EDSP2 and SEMDJL1**	**1**	***0***	**Bgn**
R-HSA-3560783	**Defective B4GALT7 causes EDS, progeroid type**	**1**	***0***	**Bgn**
R-HSA-418457	**cGMP effects**	**1**	***0***	**Cyct**
R-HSA-3560801	**Defective B3GAT3 causes JDSSDHD**	**1**	***0***	**Bgn**
R-HSA-3229121	**Glycogen storage diseases**	**1**	***0***	**Gyg1**
R-HSA-2022857	**Keratan sulfate degradation**	**1**	***0***	**Prelp**
R-HSA-77346	**Beta oxidation of decanoyl-CoA to octanoyl-CoA-CoA**	**1**	***0***	**Mecr**
R-HSA-6798163	**Choline catabolism**	**1**	***0***	**Aldh7a1**
R-HSA-2022870	**Chondroitin sulfate biosynthesis**	**1**	***0***	**Bgn**
R-HSA-174577	**Activation of C3 and C5**	**1**	***0***	**C3**
R-HSA-1237112	**Methionine salvage pathway**	**1**	***0***	**Got1**
R-HSA-5603041	**IRAK4 deficiency (TLR2/4)**	**1**	***0***	**Cd36**
R-HSA-2024101	**CS/DS degradation**	**1**	***0***	**Bgn**
R-HSA-8853336	**Signaling by plasma membrane FGFR1 fusions**	**1**	***0***	**Erlin2**
R-HSA-3322077	**Glycogen synthesis**	**1**	***0***	**Gyg1**

R-HSA-3371599	**Defective HLCS causes multiple carboxylase deficiency**	**1**	***0***	**Mccc2**
R-HSA-203927	**MicroRNA (miRNA) biogenesis**	**1**	***0***	**Prkra**
R-HSA-3323169	**Defects in biotin (Btn) metabolism**	**1**	***0***	**Mccc2**
R-HSA-434313	**Intracellular metabolism of fatty acids regulates insulin secretion**	**1**	***0***	**Cd36**
R-HSA-446205	**Synthesis of GDP-mannose**	**1**	***0***	**Mpi**
R-HSA-1971475	**A tetrasaccharide linker sequence is required for GAG synthesis**	**1**	***0***	**Bgn**
R-HSA-5602498	**MyD88 deficiency (TLR2/4)**	**1**	***0***	**Cd36**
R-HSA-2022854	**Keratan sulfate biosynthesis**	**1**	***0***	**Prelp**
R-HSA-5686938	**Regulation of TLR by endogenous ligand**	**1**	***0***	**Cd36**
R-HSA-3000471	**Scavenging by Class B Receptors**	**1**	***0***	**Cd36**
R-HSA-8963888	**Chylomicron assembly**	**1**	***0***	**P4hb**
R-HSA-196780	**Biotin transport and metabolism**	**1**	***0***	**Mccc2**
R-HSA-8866423	**VLDL assembly**	**1**	***0***	**P4hb**
R-HSA-1638074	**Keratan sulfate/keratin metabolism**	**1**	***0***	**Prelp**
R-HSA-70221	**Glycogen breakdown (glycogenolysis)**	**1**	***0***	**Gyg1**
R-HSA-204174	**Regulation of pyruvate dehydrogenase (PDH) complex**	**1**	***0***	**Dlat**
R-HSA-176033	**Interactions of Vpr with host cellular proteins**	**1**	***0***	**Slc25a4**
R-HSA-5609975	**Diseases associated with glycosylation precursor biosynthesis**	**1**	***0***	**Mpi**
R-HSA-83936	**Transport of nucleosides and free purine and pyrimidine bases across the plasma membrane**	**1**	***0***	**Slc25a4**
R-HSA-392154	**Nitric oxide stimulates guanylate cyclase**	**1**	***0***	**Cyct**
R-HSA-74217	**Purine salvage**	**1**	***0***	**Hprt1**
R-HSA-3296482	**Defects in vitamin and cofactor metabolism**	**1**	***0***	**Mccc2**
R-HSA-2022377	**Metabolism of Angiotensinogen to Angiotensins**	**1**	***0***	**Ctsd**
R-HSA-6802948	**Signaling by high-kinase activity BRAF mutants**	**1**	***0***	**Vcl**
R-HSA-5674135	**MAP2K and MAPK activation**	**1**	***0***	**Vcl**
R-HSA-1483166	**Synthesis of PA**	**1**	***0***	**Gpd1**
R-HSA-8963693	**Aspartate and asparagine metabolism**	**1**	***0***	**Got1**
R-HSA-71064	**Lysine catabolism**	**1**	***0***	**Aldh7a1**
R-HSA-1442490	**Collagen degradation**	**1**	***0***	**Ctsd**
R-HSA-8982491	**Glycogen metabolism**	**1**	***0***	**Gyg1**
R-HSA-72695	**Formation of the ternary complex, and subsequently, the 43S complex**	**1**	***0***	**Rps9**
R-HSA-77286	**mitochondrial fatty acid beta-oxidation of saturated fatty acids**	**1**	***0***	**Mecr**
R-HSA-1793185	**Chondroitin sulfate/dermatan sulfate metabolism**	**1**	***0***	**Bgn**
R-HSA-3371571	**HSF1-dependent transactivation**	**1**	***0***	**Cryab**
R-HSA-72649	**Translation initiation complex formation**	**1**	***0***	**Rps9**
R-HSA-6802946	**Signaling by moderate kinase activity BRAF mutants**	**1**	***0***	**Vcl**
R-HSA-6802955	**Paradoxical activation of RAF signaling by kinase inactive BRAF**	**1**	***0***	**Vcl**
R-HSA-72702	**Ribosomal scanning and start codon recognition**	**1**	***0***	**Rps9**
R-HSA-1638091	**Heparan sulfate/heparin (HS-GAG) metabolism**	**1**	***0***	**Bgn**
R-HSA-8956321	**Nucleotide salvage**	**1**	***0***	**Hprt1**
R-HSA-6802952	**Signaling by BRAF and RAF fusions**	**1**	***0***	**Vcl**
R-HSA-73817	**Purine ribonucleoside monophosphate biosynthesis**	**1**	***0***	**Adssl1**
R-HSA-6802949	**Signaling by RAS mutants**	**1**	***0***	**Vcl**
R-HSA-70268	**Pyruvate metabolism**	**1**	***2***	**Dlat**
R-HSA-1650814	**Collagen biosynthesis and modifying enzymes**	**1**	***0***	**P4hb**
R-HSA-6790901	**rRNA modification in the nucleus and cytosol**	**1**	***0***	**Rps9**
R-HSA-5362517	**Signaling by Retinoic Acid**	**1**	***0***	**Dlat**

R-HSA-72689	**Formation of a pool of free 40S subunits**	**1**	***0***	**Rps9**
R-HSA-5678895	**Defective CFTR causes cystic fibrosis**	**1**	***0***	**Erlin2**
R-HSA-72764	**Eukaryotic Translation Termination**	**1**	***0***	**Rps9**
R-HSA-2559584	**Formation of Senescence-Associated Heterochromatin Foci (SAHF)**	**1**	***0***	**Hist1h1b**
R-HSA-2168880	**Scavenging of heme from plasma**	**1**	***0***	**Hpx**
R-HSA-8956320	**Nucleobase biosynthesis**	**1**	***0***	**Adssl1**
R-HSA-1799339	**SRP-dependent cotranslational protein targeting to membrane**	**1**	***0***	**Rps9**
R-HSA-975956	**Nonsense Mediated Decay (NMD) independent of the Exon Junction Complex (EJC)**	**1**	***0***	**Rps9**
R-HSA-3371497	**HSP90 chaperone cycle for steroid hormone receptors (SHR)**	**1**	***0***	**Dync1h1**
R-HSA-156902	**Peptide chain elongation**	**1**	***0***	**Rps9**
R-HSA-5663084	**Diseases of carbohydrate metabolism**	**1**	***0***	**Gyg1**
R-HSA-8963898	**Plasma lipoprotein assembly**	**1**	***0***	**P4hb**
R-HSA-201688	**WNT mediated activation of DVL**	**1**	***1***	**Dbt**
R-HSA-6802957	**Oncogenic MAPK signaling**	**1**	***0***	**Vcl**
R-HSA-192823	**Viral mRNA Translation**	**1**	***1***	**Rps9**
R-HSA-211000	**Gene Silencing by RNA**	**1**	***0***	**Prkra**
R-HSA-5620924	**Intraflagellar transport**	**1**	***1***	**Dynlrb1**
R-HSA-5358346	**Hedgehog ligand biogenesis**	**1**	***0***	**P4hb**
R-HSA-2408557	**Selenocysteine synthesis**	**1**	***0***	**Rps9**
R-HSA-166663	**Initial triggering of complement**	**1**	***0***	**C3**
R-HSA-1474290	**Collagen formation**	**1**	***0***	**P4hb**
R-HSA-400451	**Free fatty acids regulate insulin secretion**	**1**	***1***	**Cd36**
R-HSA-5619084	**ABC transporter disorders**	**1**	***0***	**Erlin2**
R-HSA-8949613	**Cristae formation**	**1**	***2***	**Immt**
R-HSA-156827	**L13a-mediated translational silencing of Ceruloplasmin expression**	**1**	***0***	**Rps9**
R-HSA-72706	**GTP hydrolysis and joining of the 60S ribosomal subunit**	**1**	***0***	**Rps9**
R-HSA-72662	**Activation of the mRNA upon binding of the cap-binding complex and eIFs, and subsequent binding to 43S**	**1**	***0***	**Rps9**
R-HSA-156842	**Eukaryotic Translation Elongation**	**1**	***0***	**Rps9**
R-HSA-2672351	**Stimuli-sensing channels**	**1**	***0***	**Casq2**
R-HSA-9020933	**Interleukin-23 signaling**	**1**	***1***	**P4hb**
R-HSA-141444	**Amplification of signal from unattached kinetochores via a MAD2 inhibitory signal**	**1**	***2***	**Dync1h1**
R-HSA-141424	**Amplification of signal from the kinetochores**	**1**	***2***	**Dync1h1**
R-HSA-437239	**Recycling pathway of L1**	**1**	***0***	**Rdx**
R-HSA-382556	**ABC-family proteins mediated transport**	**1**	***0***	**Erlin2**
R-HSA-977606	**Regulation of Complement cascade**	**1**	***2***	**C3**
R-HSA-425397	**Transport of vitamins, nucleosides, and related molecules**	**1**	***0***	**Slc25a4**
R-HSA-69618	**Mitotic Spindle Checkpoint**	**1**	***2***	**Dync1h1**
R-HSA-5602358	**Diseases associated with the TLR signaling cascade**	**1**	***0***	**Cd36**
R-HSA-5260271	**Diseases of Immune System**	**1**	***0***	**Cd36**
R-HSA-2467813	**Separation of Sister Chromatids**	**1**	***0***	**Dync1h1**
R-HSA-1614635	**Sulfur amino acid metabolism**	**1**	***0***	**Got1**
R-HSA-927802	**Nonsense-Mediated Decay (NMD)**	**1**	***0***	**Rps9**
R-HSA-975957	**Nonsense Mediated Decay (NMD) enhanced by the Exon Junction Complex (EJC)**	**1**	***0***	**Rps9**
R-HSA-166658	**Complement cascade**	**1**	***2***	**C3**
R-HSA-5663220	**RHO GTPases Activate Formins**	**1**	***0***	**Dync1h1**
R-HSA-2500257	**Resolution of Sister Chromatid Cohesion**	**1**	***0***	**Dync1h1**
R-HSA-6809371	**Formation of the cornified envelope**	**1**	***0***	**Krt10**
R-HSA-2408522	**Selenoamino acid metabolism**	**1**	***0***	**Rps9**
R-HSA-68882	**Mitotic Anaphase**	**1**	***0***	**Dync1h1**

R-HSA-6807878	**COPI-mediated anterograde transport**	**1**	***0***	**Dync1h1**
R-HSA-1839124	**FGFR1 mutant receptor activation**	**1**	***1***	**Erlin2**
R-HSA-446219	**Synthesis of substrates in N-glycan biosythesis**	**1**	***0***	**Mpi**
R-HSA-72737	**Cap-dependent Translation Initiation**	**1**	***0***	**Rps9**
R-HSA-72613	**Eukaryotic Translation Initiation**	**1**	***0***	**Rps9**
R-HSA-168273	**Influenza Viral RNA Transcription and Replication**	**1**	***1***	**Rps9**
R-HSA-9010553	**Regulation of expression of SLITs and ROBOs**	**1**	***0***	**Rps9**
R-HSA-983712	**Ion channel transport**	**1**	***0***	**Casq2**
R-HSA-418346	**Platelet homeostasis**	**1**	***0***	**Cyct**
R-HSA-2151201	**Transcriptional activation of mitochondrial biogenesis**	**1**	***1***	**Sod2**
R-HSA-422356	**Regulation of insulin secretion**	**1**	***1***	**Cd36**
R-HSA-446193	**Biosynthesis of the N-glycan precursor (dolichol lipid-linked oligosaccharide, LLO) and transfer to a nascent protein**	**1**	***0***	**Mpi**
R-HSA-174824	**Plasma lipoprotein assembly, remodeling, and clearance**	**1**	***0***	**P4hb**
R-HSA-5655302	**Signaling by FGFR1 in disease**	**1**	***1***	**Erlin2**
R-HSA-2559586	**DNA Damage/Telomere Stress Induced Senescence**	**1**	***2***	**Hist1h1b**
R-HSA-1236974	**ER-Phagosome pathway**	**1**	***0***	**Cd36**
R-HSA-6805567	**Keratinization**	**1**	***0***	**Krt10**
R-HSA-3371556	**Cellular response to heat stress**	**1**	***0***	**Cryab**
R-HSA-2555396	**Mitotic Metaphase and Anaphase**	**1**	***0***	**Dync1h1**
R-HSA-1236975	**Antigen processing-Cross presentation**	**1**	***0***	**Cd36**
R-HSA-1226099	**Signaling by FGFR in disease**	**1**	***1***	**Erlin2**
R-HSA-163685	**Integration of energy metabolism**	**1**	***1***	**Cd36**
R-HSA-5689880	**Ub-specific processing proteases**	**1**	***1***	**Vdac3**
R-HSA-5632684	**Hedgehog 'on' state**	**1**	***0***	**Rdx**
R-HSA-5619115	**Disorders of transmembrane transporters**	**1**	***0***	**Erlin2**
R-HSA-196849	**Metabolism of water-soluble vitamins and cofactors**	**1**	***0***	**Mccc2**
R-HSA-168255	**Influenza Life Cycle**	**1**	***2***	**Rps9**
R-HSA-5673001	**RAF/MAP kinase cascade**	**1**	***0***	**Vcl**
R-HSA-375276	**Peptide ligand-binding receptors**	**1**	***0***	**C3**
R-HSA-199977	**ER to Golgi Anterograde Transport**	**1**	***0***	**Dync1h1**
R-HSA-1483206	**Glycerophospholipid biosynthesis**	**1**	***0***	**Gpd1**
R-HSA-168254	**Influenza Infection**	**1**	***3***	**Rps9**
R-HSA-948021	**Transport to the Golgi and subsequent modification**	**1**	***0***	**Dync1h1**
R-HSA-5684996	**MAPK1/MAPK3 signaling**	**1**	***0***	**Vcl**
R-HSA-75153	**Apoptotic execution phase**	**1**	***4***	**Hist1h1b**
R-HSA-2980736	**Peptide hormone metabolism**	**1**	***1***	**Ctsd**
R-HSA-69620	**Cell Cycle Checkpoints**	**1**	***2***	**Dync1h1**
R-HSA-400253	**Circadian Clock**	**1**	***2***	**Dbt**
R-HSA-166058	**MyD88:MAL(TIRAP) cascade initiated on plasma membrane**	**1**	***1***	**Cd36**
R-HSA-168188	**Toll Like Receptor TLR6:TLR2 Cascade**	**1**	***1***	**Cd36**
R-HSA-168179	**Toll Like Receptor TLR1:TLR2 Cascade**	**1**	***1***	**Cd36**
R-HSA-181438	**Toll Like Receptor 2 (TLR2) Cascade**	**1**	***1***	**Cd36**
R-HSA-376176	**Signaling by ROBO receptors**	**1**	***0***	**Rps9**
R-HSA-196854	**Metabolism of vitamins and cofactors**	**1**	***0***	**Mccc2**
R-HSA-198933	**Immunoregulatory interactions between a Lymphoid and a non-Lymphoid cell**	**1**	***7***	**C3**
R-HSA-9607240	**FLT3 Signaling**	**1**	***1***	**Vcl**
R-HSA-983169	**Class I MHC mediated antigen processing & presentation**	**1**	***0***	**Cd36**
R-HSA-166016	**Toll Like Receptor 4 (TLR4) Cascade**	**1**	***2***	**Cd36**
R-HSA-72766	**Translation**	**1**	***0***	**Rps9**
R-HSA-2559583	**Cellular Senescence**	**1**	***2***	**Hist1h1b**
R-HSA-162909	**Host Interactions of HIV factors**	**1**	***0***	**Slc25a4**

R-HSA-1483257	**Phospholipid metabolism**	**1**	***2***	**Gpd1**
R-HSA-418555	**G alpha (s) signalling events**	**1**	***0***	**Cyct**
R-HSA-168898	**Toll-like Receptor Cascades**	**1**	***2***	**Cd36**
R-HSA-5683057	**MAPK family signaling cascades**	**1**	***1***	**Vcl**
R-HSA-5688426	**Deubiquitination**	**1**	***1***	**Vdac3**
R-HSA-373076	**Class A/1 (Rhodopsin-like receptors)**	**1**	***0***	**C3**
R-HSA-425407	**SLC-mediated transmembrane transport**	**1**	***0***	**Slc25a4**
R-HSA-109581	**Apoptosis**	**1**	***5***	**Hist1h1b**
R-HSA-5357801	**Programmed Cell Death**	**1**	***5***	**Hist1h1b**
R-HSA-449836	**Other interleukin signaling**	**1**	***1***	**Vcl**
R-HSA-162906	**HIV Infection**	**1**	***2***	**Slc25a4**
R-HSA-195258	**RHO GTPase Effectors**	**1**	***3***	**Dync1h1**
R-HSA-500792	**GPCR ligand binding**	**1**	***0***	**C3**
R-HSA-418594	**G alpha (i) signalling events**	**1**	***1***	**C3**
R-HSA-194315	**Signaling by Rho GTPases**	**1**	***3***	**Dync1h1**
R-HSA-201681	**TCF dependent signaling in response to WNT**	**1**	***3***	**Dbt**
R-HSA-195721	**Signaling by WNT**	**1**	***3***	**Dbt**
R-HSA-169131	**Inhibition of PKR**	**0**	***2***	
R-HSA-194306	**Neurophilin interactions with VEGF and VEGFR**	**0**	***1***	
R-HSA-195399	**VEGF binds to VEGFR leading to receptor dimerization**	**0**	***1***	
R-HSA-194313	**VEGF ligand-receptor interactions**	**0**	***1***	
R-HSA-193634	**Axonal growth inhibition (RHOA activation)**	**0**	***1***	
R-HSA-193697	**p75NTR regulates axonogenesis**	**0**	***1***	
R-HSA-111457	**Release of apoptotic factors from the mitochondria**	**0**	***1***	
R-HSA-416700	**Other semaphorin interactions**	**0**	***1***	
R-HSA-189451	**Heme biosynthesis**	**0**	***1***	
R-HSA-1368071	**NR1D1 (REV-ERBA) represses gene expression**	**0**	***2***	
R-HSA-1362409	**Mitochondrial iron-sulfur cluster biogenesis**	**0**	***2***	
R-HSA-1660502	**PIPs transport between early and late endosome membranes**	**0**	***2***	
R-HSA-1660537	**PIPs transport between early endosome and Golgi membranes**	**0**	***2***	
R-HSA-1660508	**PIPs transport between late endosome and Golgi membranes**	**0**	***2***	
R-HSA-189445	**Metabolism of porphyrins**	**0**	***1***	
R-HSA-171306	**Packaging Of Telomere Ends**	**0**	***2***	
R-HSA-75892	**Platelet Adhesion to exposed collagen**	**0**	***2***	
R-HSA-399954	**Sema3A PAK dependent Axon repulsion**	**0**	***1***	
R-HSA-446343	**Localization of the PINCH-ILK-PARVIN complex to focal adhesions**	**0**	***1***	
R-HSA-168276	**NS1 Mediated Effects on Host Pathways**	**0**	***2***	
R-HSA-390450	**Folding of actin by CCT/TriC**	**0**	***2***	
R-HSA-168316	**Assembly of Viral Components at the Budding Site**	**0**	***1***	
R-HSA-446388	**Regulation of cytoskeletal remodeling and cell spreading by IPP complex components**	**0**	***1***	
R-HSA-5627117	**RHO GTPases Activate ROCKs**	**0**	***1***	
R-HSA-418217	**G beta:gamma signalling through PLC beta**	**0**	***1***	
R-HSA-8983711	**OAS antiviral response**	**0**	***1***	
R-HSA-8866907	**Activation of the TFAP2 (AP-2) family of transcription factors**	**0**	***1***	
R-HSA-168253	**Host Interactions with Influenza Factors**	**0**	***2***	
R-HSA-168268	**Virus Assembly and Release**	**0**	***1***	
R-HSA-171286	**Synthesis and processing of ENV and VPU**	**0**	***2***	
R-HSA-918233	**TRAF3-dependent IRF activation pathway**	**0**	***1***	
R-HSA-9027277	**Erythropoietin activates Phospholipase C gamma (PLCG)**	**0**	***1***	
R-HSA-434316	**Fatty Acids bound to GPR40 (FFAR1) regulate insulin secretion**	**0**	***1***	

R-HSA-198745	**Signalling to STAT3**	**0**	*1*	
R-HSA-8875791	**MET activates STAT3**	**0**	*1*	
R-HSA-8847453	**Synthesis of PIPs in the nucleus**	**0**	*1*	
R-HSA-418990	**Adherens junctions interactions**	**0**	*1*	
R-HSA-5205685	**Pink/Parkin Mediated Mitophagy**	**0**	*2*	
R-HSA-264870	**Caspase-mediated cleavage of cytoskeletal proteins**	**0**	*1*	
R-HSA-933541	**TRAF6 mediated IRF7 activation**	**0**	*1*	
R-HSA-3134973	**LRR FLII-interacting protein 1 (LRRFIP1) activates type I IFN production**	**0**	*2*	
R-HSA-351906	**Apoptotic cleavage of cell adhesion proteins**	**0**	*2*	
R-HSA-9028335	**Activated NTRK2 signals through PI3K**	**0**	*1*	
R-HSA-389958	**Cooperation of Prefoldin and TriC/CCT in actin and tubulin folding**	**0**	*2*	
R-HSA-372708	**p130Cas linkage to MAPK signaling for integrins**	**0**	*1*	
R-HSA-1660516	**Synthesis of PIPs at the early endosome membrane**	**0**	*2*	
R-HSA-1250342	**PI3K events in ERBB4 signaling**	**0**	*1*	
R-HSA-1483248	**Synthesis of PIPs at the ER membrane**	**0**	*2*	
R-HSA-1660517	**Synthesis of PIPs at the late endosome membrane**	**0**	*2*	
R-HSA-1855204	**Synthesis of IP3 and IP4 in the cytosol**	**0**	*1*	
R-HSA-390247	**Beta-oxidation of very long chain fatty acids**	**0**	*1*	
R-HSA-1963642	**PI3K events in ERBB2 signaling**	**0**	*1*	
R-HSA-8851907	**MET activates PI3K/AKT signaling**	**0**	*1*	
R-HSA-399997	**Acetylcholine regulates insulin secretion**	**0**	*1*	
R-HSA-9027276	**Erythropoietin activates Phosphoinositide-3-kinase (PI3K)**	**0**	*1*	
R-HSA-8849471	**PTK6 Regulates RHO GTPases, RAS GTPase and MAP kinases**	**0**	*2*	
R-HSA-2892247	**POU5F1 (OCT4), SOX2, NANOG activate genes related to proliferation**	**0**	*1*	
R-HSA-982772	**Growth hormone receptor signaling**	**0**	*2*	
R-HSA-8849474	**PTK6 Activates STAT3**	**0**	*1*	
R-HSA-5690714	**CD22 mediated BCR regulation**	**0**	*2*	
R-HSA-5621480	**Dectin-2 family**	**0**	*1*	
R-HSA-418890	**Role of second messengers in netrin-1 signaling**	**0**	*1*	
R-HSA-111471	**Apoptotic factor-mediated response**	**0**	*1*	
R-HSA-175474	**Assembly Of The HIV Virion**	**0**	*2*	
R-HSA-9022692	**Regulation of MECP2 expression and activity**	**0**	*1*	
R-HSA-198203	**PI3K/AKT activation**	**0**	*1*	
R-HSA-2586552	**Signaling by Leptin**	**0**	*1*	
R-HSA-8853334	**Signaling by FGFR3 fusions in cancer**	**0**	*1*	
R-HSA-8985947	**Interleukin-9 signaling**	**0**	*1*	
R-HSA-389357	**CD28 dependent PI3K/Akt signaling**	**0**	*1*	
R-HSA-8876725	**Protein methylation**	**0**	*2*	
R-HSA-5205647	**Mitophagy**	**0**	*2*	
R-HSA-1660514	**Synthesis of PIPs at the Golgi membrane**	**0**	*2*	
R-HSA-392451	**G beta:gamma signalling through PI3Kgamma**	**0**	*1*	
R-HSA-5218921	**VEGFR2 mediated cell proliferation**	**0**	*1*	
R-HSA-421270	**Cell-cell junction organization**	**0**	*1*	
R-HSA-9603381	**Activated NTRK3 signals through PI3K**	**0**	*1*	
R-HSA-450408	**AUF1 (hnRNP D0) binds and destabilizes mRNA**	**0**	*1*	
R-HSA-3000170	**Syndecan interactions**	**0**	*2*	
R-HSA-180292	**GAB1 signalosome**	**0**	*1*	
R-HSA-1266695	**Interleukin-7 signaling**	**0**	*1*	
R-HSA-390466	**Chaperonin-mediated protein folding**	**0**	*2*	
R-HSA-109704	**PI3K Cascade**	**0**	*1*	
R-HSA-6783783	**Interleukin-10 signaling**	**0**	*1*	

R-HSA-5655291	**Signaling by FGFR4 in disease**	**0**	***1***	
R-HSA-4641262	**Disassembly of the destruction complex and recruitment of AXIN to the membrane**	**0**	***2***	
R-HSA-5663213	**RHO GTPases Activate WASPs and WAVEs**	**0**	***1***	
R-HSA-157579	**Telomere Maintenance**	**0**	***2***	
R-HSA-1483249	**Inositol phosphate metabolism**	**0**	***1***	
R-HSA-8984722	**Interleukin-35 Signalling**	**0**	***2***	
R-HSA-9006335	**Signaling by Erythropoietin**	**0**	***1***	
R-HSA-5654710	**PI-3K cascade:FGFR3**	**0**	***1***	
R-HSA-5654720	**PI-3K cascade:FGFR4**	**0**	***1***	
R-HSA-391251	**Protein folding**	**0**	***2***	
R-HSA-1839117	**Signaling by cytosolic FGFR1 fusion mutants**	**0**	***1***	
R-HSA-5654689	**PI-3K cascade:FGFR1**	**0**	***1***	
R-HSA-390918	**Peroxisomal lipid metabolism**	**0**	***1***	
R-HSA-5654695	**PI-3K cascade:FGFR2**	**0**	***1***	
R-HSA-9020956	**Interleukin-27 signaling**	**0**	***2***	
R-HSA-373755	**Semaphorin interactions**	**0**	***2***	
R-HSA-2219530	**Constitutive Signaling by Aberrant PI3K in Cancer**	**0**	***1***	
R-HSA-8854691	**Interleukin-20 family signaling**	**0**	***1***	
R-HSA-418885	**DCC mediated attractive signaling**	**0**	***1***	
R-HSA-112399	**IRS-mediated signalling**	**0**	***1***	
R-HSA-5655332	**Signaling by FGFR3 in disease**	**0**	***1***	
R-HSA-8853338	**Signaling by FGFR3 point mutants in cancer**	**0**	***1***	
R-HSA-5625886	**Activated PKN1 stimulates transcription of AR (androgen receptor) regulated genes KLK2 and KLK3**	**0**	***1***	
R-HSA-2028269	**Signaling by Hippo**	**0**	***1***	
R-HSA-73886	**Chromosome Maintenance**	**0**	***2***	
R-HSA-9008059	**Interleukin-37 signaling**	**0**	***1***	
R-HSA-2428928	**IRS-related events triggered by IGF1R**	**0**	***1***	
R-HSA-2730905	**Role of LAT2/NTAL/LAB on calcium mobilization**	**0**	***1***	
R-HSA-5250924	**B-WICH complex positively regulates rRNA expression**	**0**	***2***	
R-HSA-2428924	**IGF1R signaling cascade**	**0**	***1***	
R-HSA-4086398	**Ca2+ pathway**	**0**	***1***	
R-HSA-2404192	**Signaling by Type 1 Insulin-like Growth Factor 1 Receptor (IGF1R)**	**0**	***1***	
R-HSA-2871809	**FCERI mediated Ca+2 mobilization**	**0**	***1***	
R-HSA-5655253	**Signaling by FGFR2 in disease**	**0**	***1***	
R-HSA-1660499	**Synthesis of PIPs at the plasma membrane**	**0**	***2***	
R-HSA-3928662	**EPHB-mediated forward signaling**	**0**	***2***	
R-HSA-8940973	**RUNX2 regulates osteoblast differentiation**	**0**	***2***	
R-HSA-8874211	**CREB3 factors activate genes**	**0**	***1***	
R-HSA-264876	**Insulin processing**	**0**	***1***	
R-HSA-2424491	**DAP12 signaling**	**0**	***1***	
R-HSA-114604	**GPVI-mediated activation cascade**	**0**	***2***	
R-HSA-452723	**Transcriptional regulation of pluripotent stem cells**	**0**	***1***	
R-HSA-1632852	**Macroautophagy**	**0**	***2***	
R-HSA-1489509	**DAG and IP3 signaling**	**0**	***1***	
R-HSA-397795	**G-protein beta:gamma signalling**	**0**	***1***	
R-HSA-1236382	**Constitutive Signaling by Ligand-Responsive EGFR Cancer Variants**	**0**	***1***	
R-HSA-5637815	**Signaling by Ligand-Responsive EGFR Variants in Cancer**	**0**	***1***	
R-HSA-74751	**Insulin receptor signalling cascade**	**0**	***1***	
R-HSA-432722	**Golgi Associated Vesicle Biogenesis**	**0**	***1***	
R-HSA-912446	**Meiotic recombination**	**0**	***1***	
R-HSA-2172127	**DAP12 interactions**	**0**	***1***	
R-HSA-5625740	**RHO GTPases activate PKNs**	**0**	***1***	

R-HSA-8856828	**Clathrin-mediated endocytosis**	**0**	*1*	
R-HSA-202433	**Generation of second messenger molecules**	**0**	*1*	
R-HSA-182971	**EGFR downregulation**	**0**	*1*	
R-HSA-9034015	**Signaling by NTRK3 (TRKC)**	**0**	*1*	
R-HSA-112043	**PLC beta mediated events**	**0**	*1*	
R-HSA-936440	**Negative regulators of DDX58/IFIH1 signaling**	**0**	*1*	
R-HSA-389356	**CD28 co-stimulation**	**0**	*1*	
R-HSA-109606	**Intrinsic Pathway for Apoptosis**	**0**	*1*	
R-HSA-112040	**G-protein mediated events**	**0**	*1*	
R-HSA-2029485	**Role of phospholipids in phagocytosis**	**0**	*1*	
R-HSA-8941326	**RUNX2 regulates bone development**	**0**	*2*	
R-HSA-983695	**Antigen activates B Cell Receptor (BCR) leading to generation of second messengers**	**0**	*2*	
R-HSA-6811558	**PI5P, PP2A and IER3 Regulate PI3K/AKT Signaling**	**0**	*1*	
R-HSA-74752	**Signaling by Insulin receptor**	**0**	*1*	
R-HSA-1433557	**Signaling by SCF-KIT**	**0**	*1*	
R-HSA-8857538	**PTK6 promotes HIF1A stabilization**	**0**	*1*	
R-HSA-5250913	**Positive epigenetic regulation of rRNA expression**	**0**	*2*	
R-HSA-5674400	**Constitutive Signaling by AKT1 E17K in Cancer**	**0**	*1*	
R-HSA-8983432	**Interleukin-15 signaling**	**0**	*2*	
R-HSA-186763	**Downstream signal transduction**	**0**	*2*	
R-HSA-199418	**Negative regulation of the PI3K/AKT network**	**0**	*1*	
R-HSA-6811555	**PI5P Regulates TP53 Acetylation**	**0**	*1*	
R-HSA-354192	**Integrin alphaIIb beta3 signaling**	**0**	*2*	
R-HSA-9006921	**Integrin signaling**	**0**	*2*	
R-HSA-1483255	**PI Metabolism**	**0**	*2*	
R-HSA-202424	**Downstream TCR signaling**	**0**	*1*	
R-HSA-9006115	**Signaling by NTRK2 (TRKB)**	**0**	*1*	
R-HSA-76009	**Platelet Aggregation (Plug Formation)**	**0**	*2*	
R-HSA-216083	**Integrin cell surface interactions**	**0**	*7*	
R-HSA-8953750	**Transcriptional Regulation by E2F6**	**0**	*1*	
R-HSA-447043	**Neurofascin interactions**	**0**	*1*	
R-HSA-186797	**Signaling by PDGF**	**0**	*2*	
R-HSA-3108214	**SUMOylation of DNA damage response and repair proteins**	**0**	*1*	
R-HSA-1221632	**Meiotic synapsis**	**0**	*4*	
R-HSA-6804758	**Regulation of TP53 Activity through Acetylation**	**0**	*1*	
R-HSA-5637810	**Constitutive Signaling by EGFRvIII**	**0**	*1*	
R-HSA-5637812	**Signaling by EGFRvIII in Cancer**	**0**	*1*	
R-HSA-388841	**Costimulation by the CD28 family**	**0**	*1*	
R-HSA-2029482	**Regulation of actin dynamics for phagocytic cup formation**	**0**	*2*	
R-HSA-909733	**Interferon alpha/beta signaling**	**0**	*1*	
R-HSA-5654708	**Downstream signaling of activated FGFR3**	**0**	*1*	
R-HSA-1169410	**Antiviral mechanism by IFN-stimulated genes**	**0**	*1*	
R-HSA-5654716	**Downstream signaling of activated FGFR4**	**0**	*1*	
R-HSA-1834949	**Cytosolic sensors of pathogen-associated DNA**	**0**	*2*	
R-HSA-5693565	**Recruitment and ATM-mediated phosphorylation of repair and signaling proteins at DNA double strand breaks**	**0**	*1*	
R-HSA-1643713	**Signaling by EGFR in Cancer**	**0**	*1*	
R-HSA-5654696	**Downstream signaling of activated FGFR2**	**0**	*1*	
R-HSA-5654687	**Downstream signaling of activated FGFR1**	**0**	*1*	
R-HSA-1227986	**Signaling by ERBB2**	**0**	*1*	
R-HSA-451927	**Interleukin-2 family signaling**	**0**	*2*	
R-HSA-5607764	**CLEC7A (Dectin-1) signaling**	**0**	*2*	
R-HSA-111465	**Apoptotic cleavage of cellular proteins**	**0**	*4*	
R-HSA-9009391	**Extra-nuclear estrogen signaling**	**0**	*1*	

R-HSA-8866910	**TFAP2 (AP-2) family regulates transcription of growth factors and their receptors**	**0**	*3*	
R-HSA-381038	**XBP1(S) activates chaperone genes**	**0**	*1*	
R-HSA-162599	**Late Phase of HIV Life Cycle**	**0**	*2*	
R-HSA-2219528	**PI3K/AKT Signaling in Cancer**	**0**	*1*	
R-HSA-3769402	**Deactivation of the beta-catenin transactivating complex**	**0**	*2*	
R-HSA-5687128	**MAPK6/MAPK4 signaling**	**0**	*1*	
R-HSA-5693606	**DNA Double Strand Break Response**	**0**	*1*	
R-HSA-8986944	**Transcriptional Regulation by MECP2**	**0**	*1*	
R-HSA-983705	**Signaling by the B Cell Receptor (BCR)**	**0**	*4*	
R-HSA-381070	**IRE1alpha activates chaperones**	**0**	*1*	
R-HSA-162587	**HIV Life Cycle**	**0**	*2*	
R-HSA-187037	**Signaling by NTRK1 (TRKA)**	**0**	*2*	
R-HSA-168928	**DDX58/IFIH1-mediated induction of interferon-alpha/beta**	**0**	*2*	
R-HSA-2029480	**Fcgamma receptor (FCGR) dependent phagocytosis**	**0**	*2*	
R-HSA-1236394	**Signaling by ERBB4**	**0**	*1*	
R-HSA-373752	**Netrin-1 signaling**	**0**	*2*	
R-HSA-195253	**Degradation of beta-catenin by the destruction complex**	**0**	*2*	
R-HSA-3858494	**Beta-catenin independent WNT signaling**	**0**	*1*	
R-HSA-111885	**Opioid Signalling**	**0**	*1*	
R-HSA-4090294	**SUMOylation of intracellular receptors**	**0**	*3*	
R-HSA-193704	**p75 NTR receptor-mediated signalling**	**0**	*1*	
R-HSA-5654743	**Signaling by FGFR4**	**0**	*1*	
R-HSA-202403	**TCR signaling**	**0**	*1*	
R-HSA-5654741	**Signaling by FGFR3**	**0**	*1*	
R-HSA-432720	**Lysosome Vesicle Biogenesis**	**0**	*2*	
R-HSA-5654736	**Signaling by FGFR1**	**0**	*1*	
R-HSA-4551638	**SUMOylation of chromatin organization proteins**	**0**	*2*	
R-HSA-450531	**Regulation of mRNA stability by proteins that bind AU-rich elements**	**0**	*1*	
R-HSA-9612973	**Autophagy**	**0**	*2*	
R-HSA-212165	**Epigenetic regulation of gene expression**	**0**	*2*	
R-HSA-3928664	**Ephrin signaling**	**0**	*1*	
R-HSA-1500620	**Meiosis**	**0**	*4*	
R-HSA-177929	**Signaling by EGFR**	**0**	*1*	
R-HSA-201722	**Formation of the beta-catenin:TCF transactivating complex**	**0**	*2*	
R-HSA-5654738	**Signaling by FGFR2**	**0**	*1*	
R-HSA-1474165	**Reproduction**	**0**	*4*	
R-HSA-446652	**Interleukin-1 family signaling**	**0**	*1*	
R-HSA-4420097	**VEGFA-VEGFR2 Pathway**	**0**	*4*	
R-HSA-5357905	**Regulation of TNFR1 signaling**	**0**	*1*	
R-HSA-190236	**Signaling by FGFR**	**0**	*1*	
R-HSA-8878166	**Transcriptional regulation by RUNX2**	**0**	*2*	
R-HSA-194138	**Signaling by VEGF**	**0**	*5*	
R-HSA-2454202	**Fc epsilon receptor (FCERI) signaling**	**0**	*3*	
R-HSA-5621481	**C-type lectin receptors (CLRs)**	**0**	*2*	
R-HSA-199992	**trans-Golgi Network Vesicle Budding**	**0**	*3*	
R-HSA-75893	**TNF signaling**	**0**	*1*	
R-HSA-166520	**Signaling by NTRKs**	**0**	*2*	
R-HSA-5693532	**DNA Double-Strand Break Repair**	**0**	*1*	
R-HSA-5619507	**Activation of HOX genes during differentiation**	**0**	*2*	
R-HSA-5617472	**Activation of anterior HOX genes in hindbrain development during early embryogenesis**	**0**	*2*	
R-HSA-8848021	**Signaling by PTK6**	**0**	*4*	
R-HSA-9006927	**Signaling by Non-Receptor Tyrosine Kinases**	**0**	*4*	

R-HSA-2682334	**EPH-Ephrin signaling**	**0**	***3***	
R-HSA-381119	**Unfolded Protein Response (UPR)**	**0**	***2***	
R-HSA-913531	**Interferon Signaling**	**0**	***2***	
R-HSA-1257604	**PIP3 activates AKT signaling**	**0**	***1***	
R-HSA-5633007	**Regulation of TP53 Activity**	**0**	***1***	
R-HSA-73887	**Death Receptor Signalling**	**0**	***2***	
R-HSA-73894	**DNA Repair**	**0**	***1***	
R-HSA-9006925	**Intracellular signaling by second messengers**	**0**	***1***	
R-HSA-3108232	**SUMO E3 ligases SUMOylate target proteins**	**0**	***5***	
R-HSA-2990846	**SUMOylation**	**0**	***5***	
R-HSA-3700989	**Transcriptional Regulation by TP53**	**0**	***1***	
